# On the Structure of Self-Compassion: A Meta-Analytic Confirmatory Factor Analysis of the Self-Compassion Scale

**DOI:** 10.1177/10731911251347463

**Published:** 2025-06-25

**Authors:** Matthew Bourke

**Affiliations:** 1 School of Human Movement and Nutrition Sciences, The University of Queensland, Brisbane, Australia

**Keywords:** self-compassion, self-criticism, self-kindness, meta-analysis, factor analysis

## Abstract

There has been much debate relating to the factor structure of the Self-Compassion Scale (SCS), and failure to reach a consensus has the potential to hold the field back from moving forward. Therefore, the aim of this study was to synthesize the factor structure of the SCS from diverse samples using meta-analytic structural equation modeling. Original research studies reporting on the factor structure of the SCS were identified by searching three online databases. The individual item correlation coefficient matrix was extracted from each of the included studies. Two-stage meta-analytic structural equation modeling was used to examine the dimensionality of the SCS by aggregating data across the original research studies. A total of 27 unique studies were included in the meta-analytic structural equation model. Results demonstrated that, after accounting for participant acquiescent response style, a bifactor structure with six specific factors and a single global general factor fit the data the best. These results suggest that self-compassion is a bipolar construct ranging from being entirely uncompassionate to oneself to entirely compassionate to oneself. Implications of these findings on assessment and reporting are discussed.

## Introduction

Compassion is a feeling that arises when witnessing another person suffering which stimulates a desire to help (Goetz et al., 2010). Compassion is distinctive from other emotional responses such as sadness, love, or distress, and motivates specific patterns of behaviors compared to these emotions (Goetz et al., 2010). Drawing on Buddhist philosophies, and the recognition that compassion is omni-directional, meaning that those who have the capacity feel compassion for others have the ability to feel compassion for themselves, Neff (2003a) introduced the concept of self-compassion. Broadly defined, self-compassion is how an individual relates to themselves in instances of perceived failure, inadequacy, and personal suffering (Neff, 2023). Although alternative models of self-compassion have been proposed (e.g., Gilbert, 2005), the most regularly applied conceptualization of self-compassion is comprised of three main elements: self-kindness (extending kindness to oneself rather than being harsh), common humanity (recognizing that suffering is part of the human condition and not feeling isolated in ones suffering), and mindfulness (being able to not overidentify with negative feelings yet not also ignoring them altogether; Neff, 2003a).

Research publications on the topic of self-compassion have grown exponentially since 2003 (Neff, 2023). Several lines of inquiry have persisted relating to self-compassion, and the extant literature has demonstrated that self-compassion is related to a reduction in negative thinking and improved emotional regulation (Inwood & Ferrari, 2018), and positively related to adaptive coping and inversely associated with maladaptive coping strategies (Ewert et al., 2021). Consequently, research has demonstrated that higher levels of self-compassion are related to lower levels of depression and anxiety (Lou et al., 2022; MacBeth & Gumley, 2012) and may be a protective factor against body image concerns and disordered eating behaviors (Braun et al., 2016). Building on observational research, meta-analyses of self-compassion-focused interventions have demonstrated that improving self-compassion may be a highly effective approach to improving symptoms of depression, anxiety, stress, eating psychopathology, and negative body image (Ferrari et al., 2019; Turk & Waller, 2020).

Very nearly all existing research has used Neff’s Self-Compassion Scale (SCS; Neff, 2003b), or one of its derivatives such as the SCS Short-Form (Raes et al., 2011) or the SCS for Youth (Neff et al., 2021), contributing to a unified body of literature. The SCS comprises of six psychometrically sound subscales—three positively worded which assess self-kindness, common-humanity, and mindfulness, and three negatively worded which assess self-judgment, isolation, and over-identification. The factor structure of these six specific factors is well supported. Additionally, there is growing agreement that the SCS is represented by a bifactor structure (Marsh et al., 2023). However, there is much debate about how many general factors there are. There exist two main camps, those who argue that self-compassion can be understood on a single bipolar continuum from uncompassionate self-responding at one end to compassionate self-responding at the other, and those who argue that compassionate and uncompassionate self-responding are distinct correlated general factors (Ferrari et al., 2022). The argument that compassionate self-responding and uncompassionate self-responding are separate general factors comes largely from the observation that the association between uncompassionate self-responding with psychopathology is substantially larger in magnitude compared to the association between compassionate self-responding with psychopathology (Muris & Petrocchi, 2017; Muris et al., 2016). However, some academics have brought into question the validity of this argument, stating that there is no cogent argument that variations at either end of a bipolar continuum need to have the same strength of association with outcomes of interest (Neff, 2022). On the other hand, arguments that compassionate and uncompassionate self-responding exist along a bipolar continuum come largely from bifactor exploratory structural equation models, the logic of which has recently been brought into question (Marsh et al., 2023).

Failing to achieve a consensus on the factor structure of self-compassion has several practical implications. If self-compassion is a bipolar construct, it implies that people cannot display simultaneously high (or simultaneously low) levels of compassionate self-responding and uncompassionate self-responding traits. On the other hand, if compassionate self-responding and uncompassionate self-responding are distinct factors, this implies that, to some extent, an individual can simultaneously display (or not display) both compassionate and uncompassionate traits. This will have implications to how self-compassion can be incorporated into psychotherapy (Neff & Germer, 2022). Assuming self-compassion is a bipolar construct, all self-compassion-focused therapy should have common goal of moving people along the self-compassion continuum away from uncompassionate self-responding towards compassionate self-responding. Alternatively, assuming that compassionate self-responding and uncompassionate self-responding are distinct but correlated traits would mean that different and targeted approaches may be needed to increase compassionate self-responding compared to approaches that would be most effective at decreasing uncompassionate self-responding. Another important implication that is yet to be resolved is determining how the SCS should be scored, either when applied to research (e.g., used as a dependent or independent variable in regression modes) or as a tool to establish clinical cut-off values. Moreover, something that has not been considered in the extant research, is whether there is psychometric evidence to support the reporting and interpretation of subscale scores in addition to scores on general factor(s).

To address the debate regarding the factor structure of the SCS, the current study used meta-analytic structural equation modeling to identify the optimal factor structure of the SCS from data collected from diverse samples and examine the psychometric properties of the identified factors. Applying meta-analysis to examine the factor structure of the SCS means that is possible to make more generalized statements about the structure of the assessment beyond the results from a specific sample or setting.

## Methods

### Included Studies

Studies were identified by searching MEDLINE via OVID, PsychInfo, and Scopus using keyword search terms for the SCS (“self compassion scale” OR “self-compassion scale” OR SCS) and factor analysis (“factor analysis” OR valid* OR structu* OR “principal component analysis” OR psychometric*) in September 2024. The search identified 3,821 unique potentially relevant titles and abstracts. Only studies that conducted a factor analysis of the SCS were included in this meta-analysis. From these 88 full texts were retrieved for review, and 27 studies were included in the meta-analysis (Table 1). Full texts were excluded for not reporting on the 26-item version of the SCS (*k* = 16), not reporting the information necessary to calculate the correlation matrix among individual items (*k* = 13), not being written in English (*k* = 11), not conducting a factor analysis (*k* = 8), being an unpublished thesis (*k* = 7), reporting on a duplicate dataset to another study included in the meta-analysis (*k* = 3), and for being inaccessible (*k* = 2). A full list of excluded studies at each stage of the review is available in Appendix A.

**Table 1:** Characteristics of Studies Included in the Meta-Analysis.

Study	Nation	Sample	Sample size	Sample characteristic	Language	Model used to estimate correlation matrix
Alabdulaziz et al. (2020)	Saudi Arabia	Undergraduate nursing students	*n* = 322	Age = 21.27 (range = 18–27) Female = 79%	Arabic	Six factor EFA with Varimax rotation
(Alzamil et al., 2023)	Saudi Arabia/Egypt	Students	*n* = 1,039	Age = *nr* (range = 18–30 + years) Female = 100%	Arabic	Six lower order one higher order CFA
Buz et al. (2022)	Multiple countries (n = 15)	General population	*n* = 1,508	Age = 34.94 (range = 18–70) Women = 72%	Spanish	Two general six specific pure exploratory bifactor model
Chistopolskaya et al. (2020)	Russia	Student	*n* = 498	Age = 19.3 (range = 17–28) Female = 69%	Russian	One general two nested six specific CFA
Cleare et al. (2018)	United Kingdom	General population	*n* = 526	Age = 23 (range = 16–64) Female = 76%	English	Individual item correlation matrix provided on request
Coroiu et al. (2018)	Germany	General population	*n* = 2,510	Age = 50.23 (range = 18–70+) Female = 54%	German	Two correlated factor CFA
Costa et al., (2016)	Portugal	Clinical (borderline personality disorder, anxiety disorder, eating disorder) and general populations	*n* = 361 (validation sample *n* = 220)	Age = 25.19 (range = 13–56) Women = 84%	Portuguese	Two correlated factor CFA
Cunha et al., (2016)	Portugal	Secondary school students	*n* = 3,165	Age = 15.49 (range = 12–19) Female = 54%	Portuguese	Six correlated factor CFA
de Souza and Hutz (2016)	Brazil	General population	*n* = 759 (validation sample *n* = 432)	Age = 32.5 (range = 18–66) Female = 72%	Portuguese	Six correlated factor CFA
Gruber et al. (2023)	Germany	General population	*n* = 255	Age = 14.90 (range = 10–19 years) Female = 59%	German	Six correlated factor CFA
Holas et al., (2023)	Poland	General population	*n* = 604	Age = 47.93 (range = 18–85) Female = 46%	Polish	Two general three specific bifactor ESEM
Kocur et al., (2022)	Poland	General population	*n* = 645	Age = 29.26 (range = 14–19) Female = 62%	Polish	One general six specific bifactor ESEM
Kotsou and Leys (2016)	Belgium	General population	*n* = 1,554	Age = 42.92 (range = 15–83) Female = 88%	French	Individual item correlation matrix reported
Kumlander et al.,(2018)	Finland	Secondary school students	*n* = 1,725	Age = 16.56 Female = 50%	Finnish	Six correlated factor CFA
Martínez-Ramos et al.(2022)	Colombia	General population	*n* = 751	Age = 32.8 (range = 18–76) Female = 66%	Spanish	One general six specific bifactor ESEM
Montero-Marín et al. (2016)	Brazil, Spain	Primary care professionals	*n* = 820	Age = 45.48 Women = 78%	Spanish	Individual item correlation matrix reported
Neff (2003b)	USA	Undergraduate university students	*n* = 391	Age = 20.91 Women = 58%	English	Six correlated factor CFA
Neff et al. (2019)	Multiple countries (n = 16)	General community, students, and clinical populations	*n* = 11,685	Age = 32.29 Female = 72%	Multiple languages (*n* = 13)	One general six specific bifactor ESEM
Neff et al.(2017)	USA	General community, students, and clinical populations	*n* = 2,006	Age = 37.09 Female = 61%	English	One general six specific bifactor CFA
Pfattheicher et al. (2017)	Germany	General population	*n* = 576	Age = 37.21 Female = 58%	German	Six lower order two correlated higher order CFA
Rakhimov et al. (2023)	UK	General population	*n* = 486	Age = 39.1 (range = 18–85) Women = 59%	English	One general six specific bifactor CFA
Strickland et al. (2022)	Canada	Undergraduate university students	*n* = 1,158	Age = 19.0 Women = 73%	English	Six lower order two correlated higher order CFA
Tóth-Király et al. (2017)	Hungary	General population	*n* = 505	Age = 44.37 (range = 15–75) Female = 52%	Hungarian	One general six specific bifactor CFA
Uršič et al. (2019)	Slovenia	General population	*n* = 442	Age = 31.5 Female = 72%	Slovenian	One general six specific bifactor CFA
Veneziani et al. (2017)	Italy	General population	*n* = 522	Age = 30.1 (range = 18–72) Female = 63%	Italian	Individual item correlation matrix provided on request
Zhang et al. (2019)	USA	Clinical samples	*n* = 248	Age = 37.26 (range = 18–64) Women = 56%	English	Two correlated general six specific bifactor CFA
Zhao et al. (2022)	China	University students	*n* = 465	Age = 20.26 Women = 70%	Chinese	One general four specific bifactor CFA

*Note.* CFA = confirmatory factor analysis; EFA = exploratory factor analysis; ESEM = exploratory structural equation modeling.

### Estimation of Item Level Correlation Coefficients

Meta-analytic structural equation modeling requires that the individual item level correlation matrix from each of the included studies is known (Becker, 1996). Therefore, the item level correlation coefficients among each of the 26 items in the SCS were either recorded from studies that reported a correlation matrix with the correlation between each of the items in the SCS, or it was calculated based on the reported factor loading matrix (Gnambs & Staufenbiel, 2016). The correlations among a set of observed variables (*y*) can be estimated based on the assumption that the correlations can be explained by a smaller set of latent variables (*x*) such that the item level correlation matrix **ρ** can be expressed as:

$$L\Phi L^{T}+U$$

where L is a *y* * *x* factor loading matrix, Φ is a *x* * *x* correlation matrix of latent variables, and U is a *y* * *y* diagonal matrix of unique variance of observed variables. Where studies reported on the factor loading matrix from multiple factor models, the correlation matrix was calculated from the model that had the best overall fit statistics (assuming that all the information necessary to calculate the correlation matrix from this model was presented). Studies were excluded if they did not report on all the information necessary to calculate the item level correlation matrix, for examples studies which did not report the correlation between each factor from correlated factor models (e.g., exploratory factor analysis with oblique rotation, six factor correlated confirmatory factor analysis model). Given that some included studies reverse scored negatively worded items, and others did not, it was necessary to reverse code some of the factor loadings from individual studies that reverse scored negatively worded items to ensure that the correlation coefficients were consistently interpretable across all included studies. Specifically, negatively worded items were reverse-coded when loading onto a general factor which included both positively and negatively worded items or cross-loaded onto a positive factor (e.g., the cross-loading of self-judgment items on the self-kindness factor) and positively worded items were reverse-coded when they cross-loaded onto a negative factor (e.g., mindfulness items cross-loading on the over-identification factor) in models which allowed cross-loading (e.g., exploratory structural equation models). Additionally, the correlation between positive and negative factors was reverse-coded for studies which reverse scored negative items. Additionally, some studies did not report cross-loadings despite estimating models which allowed cross-loadings. Consistent with the methods used in a previous study (Schroeders et al., 2021), zero cross-lodgings were imputed in these instances, which has been shown to lead to unbiased estimates in the meta-analytic model (Gnambs & Staufenbiel, 2016).

### Statistical Analysis

**Figure 1.**
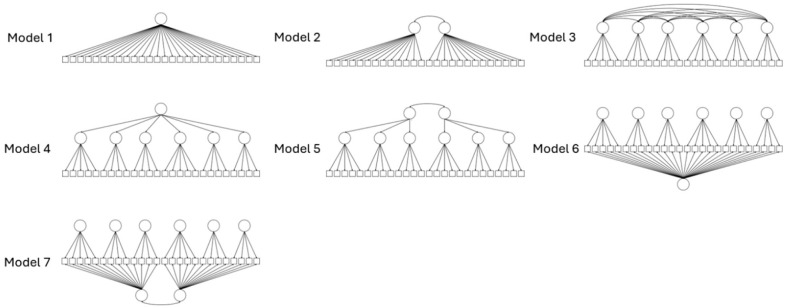
Overview of the Structural Models Assessed in Stage Two Models.

The factor structure of the SCS was examined using two-stage meta-analysis structural equation modeling (Cheung & Chan, 2005). The first of two stages involves estimating a pooled correlation matrix across multiple studies, and the second step involves fitting a structural model to the pooled correlation matrix from the first step. In the first stage, a fixed-effects meta-analysis was run to estimate the pooled correlation matrix. The chi-square test, root mean squared error of approximation (RMSEA), standardized root mean square residual (SRMR), and comparative fit index (CFI) were examined to determine if there was approximate homogeneity in the correlation matrices across included studies. A non-significant chi-square test, an RMSEA ≤ 0.06, an SRMR ≤ 0.08, and a CFI ≥ 0.95 were used to determine if there was homogeneity between the correlation matrices (Hu & Bentler, 1999). An attempt was also made to estimate a random-effects meta-analysis, however, due to the complexity of the model, the random-effects model was not able to be estimated. Assuming no further model misspecification, fixed-effects models do not lead to significant bias of factor loadings or correlations between factors from the stage two model, therefore, using a fixed-effects model in the first stage of the two-stage structural equation modeling meta-analysis provides a practical alternative even in the face of heterogeneity between correlation matrices when it is not possible to estimate a random effects model (Bloszies & Koch, 2024). In the second stage, a confirmatory factor analysis was estimated using the pooled correlation matrix estimated in stage one. Several different models that have been proposed for the SCS in the past were systematically evaluated to determine the optimal factor structure of the SCS. A series of first-order factor models (models 1–3), higher order factor models (models 4 and 5), and bifactor models (models 6 and 7) were assessed. The difference between higher order factor models and bifactor models is that higher order factors explain the correlation between lower order factors, and therefore are only related to individual items through lower order factors, whereas in bifactor models, the general factors directly account for correlations between individual items whereas the specific factors account for the shared residual variance in individual items after accounting for the variance explained by the general factor(s). Each of the estimated models can be seen in Figure 1. Acceptable model fit was assessed using a range of model fit statistics including RMSEA (≤0.06), SRMR (≤0.08), CFI (≥0.95), and Tucker-Lewis Index (TLI; ≥0.95).

Because the SCS assesses compassionate self-responding with positively worded items and uncompassionate self-responding with negatively worded items, an individual’s response style (i.e., their tendency to agree or disagree more strongly with a question regardless of the content of the question) may impact the results of the factor analysis. Specifically, the correlation between factors assessed using positively worded items and factors assessed using negatively worded items may be artificially reduced. Therefore, an acquiescence factor was added to some models to determine if the results reflect a true relationship between factors or are more likely a result of method bias. Specifically, an acquiescence factor for which all items are constrained to load equally (meaning that positive and negatively worded items load in the same direction) was added to the bifactor model with one general factor. The model fit for this model (Model 6a) was compared to the bifactor model with two general factors (Model 7). If the fit of the model with two correlated general factors is not substantially better than the model with one general factor and an acquiescence factor, it indicates that the scale is essentially bipolar and the divergence between the positively and negatively worded items is a consequence of participants response styles rather than true differences between compassionate self-responding and uncompassionate self-responding (Soto & John, 2017).

### Transparency and Openness

Screening of studies for inclusion in the meta-analysis was conducted using Covidence (Veritas Health Innovation, Melbourne, Australia, https://www.covidence.org). It is reported above how studies were identified and included in the meta-analysis. A full list of articles that were excluded are available in Appendix A. The dataset and code used to run the analyses have been made publicly available on the OSF and can be accessed at https://osf.io/k6e25/. Data analysis was conducted using R v. 4.1.3 (R Core Team, Vienna, Austria) in R Studio v. 1.3 (R Studio Team, Boston, MA) using the metaSEM (Cheung, 2015) and OpenMX packages (Neale et al., 2016). This study was not preregistered.

## Results

### Description of Included Studies

An overview of the studies included in the meta-analysis is displayed in Table 1. The median sample size of included studies was 513 (range = 222–11,685). The median average age of participants in included studies was 32.29 years (range = 14.90–50.23), and the median proportion of females/women in included studies was 66% (range = 46–100%). Three studies were conducted in the United States and Germany, two studies were conducted in Poland, Portugal, and the United Kingdom, and a single study was conducted in Belgium, Brazil, Canada, China, Columbia, Finland, Hungary, Italy, Slovenia, Russia, and Saudi Arabia. Additionally, four studies reported on combined results from samples recruited from multiple countries. The most used version of the SCS was the English version (*k* = 5), followed by the German (*k* = 3), Portuguese (*k* = 3), Spanish (*k* = 3), Arabic (*k* = 2), and the Polish (*k* = 2) versions. A single study each used the Chinese, Finnish, French, Hungarian, Italian, Slovenian, and Russian versions of the SCS. A single study also reported on combined results from multiple studies that used a range of different language versions of the SCS. Most studies recruited participants from the general population (*k* = 14), while many studies also recruited university (*k* = 6) or secondary school (*k* = 2) students. Only one study recruited exclusively from a clinical population, one study recruited primary care professions, and three studies recruited across the general population, students, and clinical populations.

### Meta-Analysis Structural Equation Model

#### Stage One

The pooled correlation matrix from the stage one meta-analysis is displayed in Table 2. The results from the stage one analysis indicated that there was significant heterogeneity in the correlation matrices among the included studies (χ^2^(9,425) = 91,996, *p* < .001, RMSEA = 0.086, SRMR = 0.165, CFI = 0.834).

**Table 2:** Estimated Correlation Matrix From the Stage One Analysis.

SCS Item	sk1	sk2	sk3	sk4	sk5	sj1	sj2	sj2	sj4	sj5	ch1	ch2	ch3	ch4	is1	is2	is3	is4	mi1	mi2	mi3	mi4	oi1	oi2	oi3
sk2	0.58																								
sk3	0.56	0.62																							
sk4	0.55	0.61	0.63																						
sk5	0.49	0.52	0.55	0.58																					
sj1	−0.31	−0.33	−0.35	−0.37	−0.38																				
sj2	−0.32	−0.33	−0.35	−0.37	−0.38	0.50																			
sj3	−0.32	−0.34	−0.34	−0.37	−0.38	0.48	0.47																		
sj4	−0.34	−0.37	−0.39	−0.42	−0.42	0.54	0.52	0.54																	
sj5	−0.33	−0.35	−0.37	−0.41	−0.40	0.48	0.50	0.49	0.53																
ch1	0.36	0.36	0.38	0.36	0.38	−0.24	−0.24	−0.24	−0.27	−0.24															
ch2	0.32	0.30	0.33	0.30	0.32	−0.20	−0.20	−0.20	−0.22	−0.19	0.42														
ch3	0.38	0.36	0.38	0.34	0.37	−0.23	−0.24	−0.23	−0.26	−0.23	0.44	0.51													
ch4	0.38	0.39	0.40	0.37	0.40	−0.27	−0.29	−0.28	−0.31	−0.30	0.41	0.39	0.45												
is1	−0.31	−0.34	−0.36	−0.37	−0.37	0.44	0.41	0.41	0.46	0.40	−0.24	−0.21	−0.24	−0.23											
is2	−0.31	−0.33	−0.35	−0.39	−0.39	0.39	0.40	0.39	0.44	0.39	−0.25	−0.21	−0.24	−0.24	0.53										
is3	−0.28	−0.30	−0.32	−0.36	−0.36	0.36	0.36	0.36	0.41	0.37	−0.23	−0.19	−0.22	−0.21	0.48	0.58									
is4	−0.32	−0.35	−0.36	−0.36	−0.37	0.42	0.41	0.41	0.46	0.40	−0.25	−0.20	−0.24	−0.25	0.51	0.53	0.50								
mi1	0.43	0.46	0.47	0.49	0.51	−0.39	−0.40	−0.40	−0.45	−0.43	0.39	0.31	0.37	0.46	−0.32	−0.33	−0.31	−0.35							
mi2	0.40	0.43	0.44	0.42	0.44	−0.32	−0.35	−0.33	−0.36	−0.37	0.37	0.30	0.36	0.46	−0.27	−0.29	−0.26	−0.31	0.59						
mi3	0.38	0.40	0.43	0.42	0.44	−0.32	−0.33	−0.33	−0.37	−0.36	0.35	0.32	0.35	0.42	−0.28	−0.29	−0.27	−0.30	0.56	0.55					
mi4	0.40	0.42	0.43	0.44	0.43	−0.29	−0.31	−0.32	−0.35	−0.34	0.34	0.28	0.33	0.41	−0.25	−0.26	−0.24	−0.27	0.51	0.50	0.47				
oi1	−0.34	−0.36	−0.38	−0.43	−0.44	0.50	0.47	0.46	0.53	0.48	−0.28	−0.23	−0.26	−0.28	0.51	0.48	0.44	0.48	−0.40	−0.31	−0.32	−0.30			
oi2	−0.33	−0.36	−0.38	−0.42	−0.42	0.48	0.47	0.45	0.51	0.47	−0.28	−0.23	−0.26	−0.27	0.50	0.47	0.43	0.47	−0.38	−0.31	−0.32	−0.29	0.58		
oi3	−0.29	−0.32	−0.33	−0.39	−0.39	0.37	0.37	0.39	0.44	0.43	−0.24	−0.19	−0.22	−0.25	0.37	0.37	0.35	0.37	−0.36	−0.27	−0.28	−-0.28	0.51	0.48	
oi4	−0.30	−0.33	−0.35	−0.39	−0.38	0.38	0.39	0.39	0.43	0.42	−0.24	−0.19	−0.22	−0.25	0.41	0.40	0.36	0.39	−0.35	−0.27	−0.27	−0.26	0.52	0.50	0.49

*Note.* ch = common humanity, is = isolation, mi = mindfulness, oi = overidentification, SCS = Self-Compassion Scale, sj = self-judgement, sk = self-kindness

#### Stage Two

The model fit statistics from each of the tested models are displayed in Table 3. Only three models demonstrated acceptable model fit, the model with six correlated specific factors (Model 3) the model with six uncorrelated specific factors and two correlated global factors (Model 7), and the model with six uncorrelated specific factors, one general factor and an acquiescence factor (Model 6a). Direct comparison between Model 3 and Model 7 showed that the model with six uncorrelated specific factors and two correlated global factors fit the data significantly better (χ^2^(12) = 2,198, *p* < .001). However, the model fit statistics for Model 7 and Model 6a were essentially the same. Therefore, the results demonstrate that, after accounting for response styles, there is a single bipolar general factor for self-compassion and six specific factors. The standardized factor loadings for individual items are displayed in Table 4.

**Table 3:** Results From the CFA Models of the SCS.

Number	Model	χ^2^	df	CFI	TLI	RMSEA	SRMR
1	Single factor	107,000	299	0.420	0.370	0.101	0.388
2	Two correlated factors	10,344	298	0.891	0.880	0.043	0.133
3	Six correlated factors	7,694	284	0.959	0.953	0.027	0.051
4	Six lower order plus one higher order	14,783	293	0.921	0.913	0.037	0.114
5	Six lower order plus two higher order	9,548	292	0.950	0.944	0.030	0.065
6	Six specific plus one global	10,838	273	0.943	0.932	0.033	0.096
6a	Six specific plus one global with acquiescence factor	5,496	272	0.972	0.966	0.023	0.043
7	Six specific plus two global	5,748	272	0.970	0.965	0.024	0.046

*Note.* CFA = confirmatory factor analysis; SCS = Self-Compassion Sale.

**Table 4:** Factor Loadings From Model 6a.

Specific factor (item number)	General self-compassion factor	Specific factor
Self-kindness (SCS 5)	0.62 (0.61, 0.62)	0.33 (0.32, 0.34)
Self-kindness (SCS 12)	0.65 (0.64, 0.65)	0.43 (0.42, 0.44)
Self-kindness (SCS 19)	0.68 (0.67, 0.68)	0.38 (0.37, 0.39)
Self-kindness (SCS 23)	0.70 (0.69, 0.71)	0.35 (0.34, 0.36)
Self-kindness (SCS 26)	0.71 (0.71, 0.72)	0.13 (0.12, 0.15)
Self-judgment (SCS 1)	−0.65 (−0.65, −0.64)	0.24 (0.22, 0.26)
Self-judgment (SCS 8)	−0.64 (−0.65, −0.64)	0.24 (0.22, 0.26)
Self-judgment (SCS 11)	−0.64 (−0.64, −0.63)	0.22 (0.20, 0.24)
Self-judgment (SCS 16)	−0.71 (−0.72, −0.70)	0.24 (0.22, 0.25)
Self-judgment (SCS 21)	−0.66 (−0.67, −0.66)	0.20 (0.18, 0.22)
Common humanity (SCS 3)	0.52 (0.52, 0.53)	0.29 (0.28, 0.30)
Common humanity (SCS 7)	0.44 (0.43, 0.45)	0.51 (0.49, 0.52)
Common humanity (SCS 10)	0.51 (0.50, 0.52)	0.48 (0.47, 0.50)
Common humanity (SCS 15)	0.58 (0.58, 0.59)	0.23 (0.22, 0.24)
Isolation (SCS 4)	−0.64 (−0.64, −0.63)	0.24 (0.23, 0.26)
Isolation (SCS 13)	−0.62 (−0.62, −0.61)	0.45 (0.44, 0.47)
Isolation (SCS 18)	−0.57 (−0.57, −0.56)	0.43 (0.42, 0.44)
Isolation (SCS 25)	−0.63 (−0.63, −0.62)	0.28 (0.27, 0.30)
Mindfulness (SCS 9)	0.72 (0.71, 0.72)	0.27 (0.25, 0.28)
Mindfulness (SCS 14)	0.65 (0.64, 0.65)	0.36 (0.34, 0.38)
Mindfulness (SCS 17)	0.63 (0.62, 0.63)	0.31 (0.29, 0.32)
Mindfulness (SCS 22)	0.61 (0.60, 0.61)	0.20 (0.18, 0.21)
Overidentified (SCS 2)	−0.72 (−0.73, −0.72)	0.20 (0.19, 0.22)
Overidentified (SCS 6)	−0.71 (−0.71, −0.70)	0.15 (0.14, 0.17)
Overidentified (SCS 20)	−0.59 (−0.60, −0.59)	0.33 (0.31, 0.35)
Overidentified (SCS 24)	−0.61 (−0.61, −0.60)	0.31 (0.29, 0.33)

*Note.* SCS = Self-Compassion Sale.

### Scoring and Interpreting the Bifactor Model of the SCS

Given that a bifactor model of SCS fits the data best, the value-added ratio (VAR) was estimated using the equation specified by Dueber and Toland (2023). A VAR >1.1 indicates that a subscale explains a meaningful amount of true variance above and beyond the global factor score for that given subscale and therefore should be reported and interpreted, a VAR between 1.0 and 1.1 indicates that although potentially redundant, there is no harm in reporting or interpreting a subscale score, and a VAR < 1 indicates that the global score is a better predictor of the true subscale score than the observed score on the subscale, and therefore subscale scores should not be reported or interpreted. The VAR for each subscale is displayed in Table 5. Apart from overidentification which was marginally below a VAR of 1.0, all other subscales had a VAR >1 which indicates that there is no harm in reporting and interpreting subscale scores in addition to the global score for self-compassion.

**Table 5:** VAR for Each Subscale.

Subscale	VAR
Self-kindness	1.11
Self-judgement	1.01
Common humanity	1.17
Isolation	1.10
Mindfulness	1.02
Overidentification	0.99

*Note.* VAR = value-added ratio.

## Discussion

Given the ongoing debate about the factor structure of the SCS, the aim of this study was to conduct a confirmatory factor analysis using meta-analysis structural equation modeling to elucidate the structure of self-compassion across diverse samples and contexts. The results demonstrated, after accounting for participant acquiescent response styles, a bifactor model with the six specific factors in addition to single global general factor for self-compassion fit the data best. These results suggest that self-compassion is a bipolar construct where people fall somewhere between two poles from being entirely uncompassionate to oneself to being entirely compassionate to oneself.

The results from this study provide an important addition to the extant literature by synthesizing results from a wide range of diverse samples and contexts and has several practical implications for the study of self-compassion. Specifically, the results from the present study suggest that a single score on a bipolar scale for self-compassion should be reported and interpreted for studies that utilize the SCS, rather than two separate scores for compassionate self-responding and uncompassionate self-responding. However, it is crucial to account for the effect of participant acquiescent response styles when scoring and interpreting the global score on the SCS. Conveniently, the self-compassionate scale is perfectly balanced with regard to the number of positively worded and negatively worded items. Therefore, taking the aggregate of positively worded and (reverse scored) negatively worded items controls for participant acquiescent responding (Soto & John, 2017). If using the SCS in structural equation modeling, researchers should model the scale with an acquiescence factor to account for participant response biases. The impact of participant response style may explain the results of previous research which has demonstrated that negative subscales from the SCS are more strongly related with indicators of psychopathology (Muris & Petrocchi, 2017), given that scales to assess the symptoms of mental health conditions include mainly negatively valenced questions. Participant acquiescence responding artificially inflates positive correlations (i.e., between two negatively worded scales) and reduces the negative correlations (i.e., between a positively worded and negatively worded scale). In addition to reporting on the general SCS, there may be some added benefit, and certainly no harm, in reporting and interpreting the scores on the specific subscales. From a practical perspective, there is a growing interest in self-compassion interventions, such as compassion-focused therapy (Ferrari et al., 2019; Kirby et al., 2017), which aim to improve self-compassion. Interventions could be assessed in terms of how well they improve overall self-compassion, but also how they affect specific subscales of the SCS which may point to the specific mechanism through which the intervention is having an effect. From a clinical perspective, Marsh et al. (2023) suggest that scores on the global factor could be examined to determine the presence of a “red flag” and scores on specific factors could be explored to determine where specific deficits may exist. From a research perspective, including specific subscale scores in models in addition to the score on the general factor may explain additional variance in outcomes being examined. It is also defensible to simply model the correlated specific factors without the inclusion of the general factors and develop latent profiles or clusters of participants based on differences in scores on the specific subscales (Eid et al., 2017). Several researchers have already taken this approach to examine the SCS (Ferrari et al., 2023; Ullrich-French & Cox, 2020). However, again it is essential to account for the impact of participant acquiescence responding, which may mean responses on positively worded items are artificially inflated in comparison to response to negatively worded items, or vice-versa depending on the response style of a participant. To account for this, each individual participant’s responses to individual items should be centered around their individual participant mean calculated from all items on the SCS without reverse scoring negative items (Soto & John, 2017).

An important caveat to the findings of this study is that the findings only relate to self-compassion as a trait. However, self-compassion is a state of mind, meaning that it is malleable and fluctuates within individuals over time (Neff, 2023). Results from analyses at the inter-individual level cannot be translated to the intra-individual level (Molenaar & Campbell, 2009). Studies employing intensive longitudinal data analysis approaches and multilevel structural equation modeling to simultaneously model the within-person and between-person structure of self-compassion may move the field forward. Preliminary evidence from such a research design has shown that compassionate self-responding and uncompassionate self-responding are distinct factors at both the within-person level of analysis (Büchner et al., 2024). Further studies that aim to replicate or dispute these findings are necessary to elucidate how self-compassion operates both as a state of mind and as an individual trait.

### Limitations

There are some limitations that must be considered when interpreting the results of the present study. First, this study was a meta-analysis without systematic review. Although a systematic process was taken to identify studies to include in the meta-analysis, the review was conducted by a single author and therefore cannot be considered a true systematic review. Additionally, several of the suggestions of the PRISMA guidelines for systematic reviews were not adhered to (e.g., assessing risk of bias of individual studies, examining reporting bias, determine certainty of evidence). Second, only studies that reported the information necessary to compute individual item correlation coefficient matrices were included in the meta-analysis. Additionally, only peer-reviewed publications which were published in academic journals were included in the meta-analysis. Although a total of 27 studies reporting on diverse samples were included in the meta-analysis, there were more studies that would have been included in the meta-analysis had researchers reported more complete results for the factor analyses they conducted, or if results published in medium other than academic journals were considered, which may have provided a more representative body of science relating to the SCS. Another limitation of the current study is that very few studies recruited clinical samples. Given that self-compassion refers to how an individual relates to themselves in instances of personal suffering, understanding how self-compassion is experienced and practiced in clinical populations may be of particular interest. Additionally, there was a significant gender imbalance in the majority of studies included in the meta-analysis, meaning that the generalizability of the results to men/males is unclear. Finally, although several included studies recruited large samples, there were several studies that recruited relatively small samples (i.e., <500 participants). Although the minimum recommended sample size for conducting factor analyses is contingent on a range of factors, with moderate communality among variables, many factors, and relatively few items loading onto each factor, such is the case with the SCS, a relatively large sample size is suggested for conducting factor analysis (Mundfrom et al., 2005). Therefore, there may have been biases in the data extracted from studies with smaller sample sizes. Although the distortion in individual studies can be compensated for in meta-analyses with a large number of studies (Gnambs & Staufenbiel, 2016), it does mean that it is less clear whether the heterogeneity observed between individual studies was the result of true differences in the factor structure between studies, or a result of substantial random error in smaller studies.

## Conclusion

The results from the present meta-analysis demonstrated that across a range of diverse samples, after controlling for participant acquiescence response style, self-compassion can be measured on a single bipolar continuum. Therefore, it is suggested that when using the SCS scale, researchers should score a single global factor for completely uncompassionate to oneself to completely compassionate to oneself. Given the bifactor structure of the SCS, there may also be added benefits to reporting on and interpreting the scores of individual subscales for specific factors in addition to the general factor. Special care needs to be taken into account for acquiescence response style when scoring subscale scores.

## Supplemental Material

sj-docx-1-asm-10.1177_10731911251347463 – Supplemental material for On the Structure of Self-Compassion: A Meta-Analytic Confirmatory Factor Analysis of the Self-Compassion Scale Supplemental material, sj-docx-1-asm-10.1177_10731911251347463 for On the Structure of Self-Compassion: A Meta-Analytic Confirmatory Factor Analysis of the Self-Compassion Scale by Matthew Bourke in Assessment
