# Supplementary material for: On the Structure of Self-Compassion: A Meta-Analytic Confirmatory Factor Analysis of the Self-Compassion Scale
Source: Assessment. 2025 Jun 25;33(4):533–45. doi: 10.1177/10731911251347463 (PMC13153440; doi:10.1177/10731911251347463)
Supplement: sj-docx-1-asm-10.1177_10731911251347463 – Supplemental material for On the Structure of Self-Compassion: A Meta-Analytic Confirmatory Factor Analysis of the Self-Compassion Scale [file sj-docx-1-asm-10.1177_10731911251347463.docx]

**List of articles excluded in title and abstract screening**

1. Thermal analysis of dry storage and transportation casks castor using COBRA-SFS
2. What else do they want? Development of a new scale for measuring contemporary forms of sexism
3. The influence of effective rainfall on modeled runoff hydrograph
4. Extensions of the S66 data set: More accurate interaction energies and angular-displaced nonequilibrium geometries
5. The Trauma Related Shame Inventory: Measuring trauma-related shame among patients with PTSD
6. Towards accurate calculation of supercapacitor electrical variables in constant power applications using new analytical closed-form expressions
7. A novel interactive fuzzy programming approach for optimization of allied closed-loop supply chains
8. Development of the school climate scale (SCS)
9. Efficient and high-bifacial CdTe-based solar cell enabled by functional designed dielectric/metal/dielectric transparent top contact via light management engineering
10. Biliary Metabolome Profiling for Evaluation of Liver Metabolism and Biliary Tract Function Related to Organ Preservation Method and Degree of Ischemia in a Porcine Model
11. Dialogues between distanced and suffering I-positions: Emotional consequences and self-compassion
12. Determination of inorganic anions in papermaking waters by ion chromatography
13. Development and validation of the Relational Needs Satisfaction Scale
14. PGAM1 regulates the glycolytic metabolism of SCs in tibetan sheep and its influence on the development of SCs
15. Diabetic foot self-care practice in women with diabetes in Iran
16. Relationships between nicotine craving, orexin-leptin levels and temperament character traits among non-treatment seeking health professionals
17. Solution combustion-based synthesis of NiO-GDC and NiO-SDC nanocomposites for low-temperature SOFC
18. Design and analysis of a highâ€gain stepâ€up/down modular dcâ€“dc converter with continuous input current and decreased voltage stress on power switches and switchedâ€capacitors
19. Managing supply chain disruptions in pharmaceutical distribution: theÂ roles of disruption orientation and mitigation strategies
20. Information technology drivers of supply chain agility: Implications for market performance
21. Simulation and optimization of dynamic waste collection routes
22. Implantation of multiple suprachoroidal electrode arrays in rabbits
23. Short communication: validation of somatic cell score-associated loci identified in a genome-wide association study in German Holstein cattle
24. The impact of rainfall distribution patterns on hydrological and hydraulic response in arid regions: case study Medina, Saudi Arabia
25. Metal Oxide Heterojunction for Photocatalytic Activities
26. Psychometric validation of gender nonspecific sexual confidence and sexual relationship scales in men and women
27. 1H chemical shifts in NMR. Part 20--anisotropic and steric effects in halogen substituent chemical shifts (SCS), a modelling and ab initio investigation
28. Principal component and cluster analyses to evaluate production and milk quality traits
29. Deep Earth rotational seismology
30. Optimization of the heterologous expression of banana glucanase in Escherichia coli
31. Evaluating a short-form Five Facet Mindfulness Questionnaire in adolescents: Evidence for a four-factor structure and invariance by time, age, and gender
32. Flash flood simulation for Tabuk City catchment, Saudi Arabia
33. Modelling Rainfall Runoff Relations Using HEC-HMS and IHACRES for a Single Rain Event in an Arid Region of Jordan
34. Impact of a vertical geothermal heat exchanger on the solar fraction of a solar cooling system
35. Self-compassion as a moderator between clinical perfectionism and psychological distress
36. 2D DIGE/MS to investigate the impact of slaughtering techniques on postmortem integrity of fish filet proteins
37. Development of a novel nomogram to predict the risk of severe compensatory sweating following endoscopic thoracic sympathectomy
38. Design and validation of a chart-based measure of the limits of spatial contrast sensitivity
39. Machine Learning to Predict Successful Opioid Dose Reduction or Stabilization After Spinal Cord Stimulation
40. Estimating flow rate in gauged and ungauged stations in Kuantan river basin using Clark method in Hec-HMS
41. A quantitative structure-activity relationship study on histamine receptor antagonists using the genetic algorithm-multi-parameter linear regression method
42. Synchronous compensators: Models verified by tests of automatic voltage regulator, reactive power control, and voltage joint control
43. Nature-based solutions for floodâ€“drought mitigation using a composite framework: a case-based approach
44. A Self-Balanced Five-Level Buck PFC EV Rectifier
45. INVESTIGATING THE INFLUENCE OF SCS AND PCS ON EXISTENCE OF HUMAN CAPITAL OF RURAL ENTREPRENEURIAL ACTIVITIES AND SMALL BUSINESSES: MANAGERIAL IMPLICATIONS
46. The impact of COVIDâ€19 pandemicâ€related stress experienced by Australian nurses
47. Dickkopf1 regulates fate decision and drives breast cancer stem cells to differentiation: an experimentally supported mathematical model
48. Hydrologic calibration and validation of SWAT in a snow-dominated Rocky Mountain watershed, Montana, U.S.A
49. Evolution-based performance prediction of star cricketers
50. Bar adsorptive microextraction and liquid chromatography-diode array detection of synthetic cannabinoids in oral fluid
51. Structural validity of the Persian version of the Suicide Capacity Scale among Iranian college students
52. Determination using synchrotron radiation-based Fourier transform infrared microspectroscopy of putative stem cells in human adenocarcinoma of the intestine: corresponding benign tissue as a template
53. The sustainable configuration of a circular economy in the agri-food supply chain: a case study of the sugar cane supply chain
54. Comparison of SWAT streamflow and water quality in an agricultural watershed using KOMPSAT-2 and Landsat land use information
55. Validation of resonance Raman spectroscopy-measured skin carotenoid status as a biomarker for fruit and vegetable intake in Korean adults
56. Examination of the utility of skin carotenoid status in estimating dietary intakes of carotenoids and fruits and vegetables: A randomized, parallel-group, controlled feeding trial
57. 3D Hierarchically Mesoporous Zinc-Nickel-Cobalt Ternary Oxide (Zn0.6Ni0.8Co1.6O4) Nanowires for High-Performance Asymmetric Supercapacitors
58. Numerical investigation of the effect of laser beam oscillation mode on the grain growth process during laser welding of aluminum alloy
59. The analysis of microstructure evolution process considering the dynamic solidification conditions and flow field in the oscillating laser welding of aluminum alloy
60. Electrophysiologic monitoring for placement of laminectomy leads for spinal cord stimulation under general anesthesia
61. Investigating the Efficacy of Stress Reduction Interventions Based on Mindfulness Principles in Improving Life Quality for Individuals with Chronic Obstructive Pulmonary Disease (COPD)
62. Assimilation of satellite-based data for hydrological mapping of precipitation and direct runoff coefficient for the Lake Urmia basin in Iran
63. Insight scale for nonclinical university students: Validity and reliability analysis
64. Self-compassion and achievement goals: A structural equation modeling approach
65. The Scales of Psychological Well-Being: A study of validity and reliability
66. Investigation of Bending Effect on Steelâ€“Concreteâ€“Steel (SCS) Sandwich Composite Sections Considering Widthâ€“Thickness Ratio and Global Provisional Codes Validation
67. Shear Resistance Behavior of Partially Sandwich Composite Structures Considering Elements Varying Dimension and Comparison Using Global Provisional Codes
68. Pattern-based transformation of SysML models into fault tree models
69. Supply chain digitalization and performance: A moderated mediation of supply chain visibility and supply chain survivability
70. Spinal cord stimulation for complex pain: Initial experience with a dual electrode, programmable, internal pulse generator
71. Measuring the Usability and Quality of Explanations of a Machine Learning Web-Based Tool for Oral Tongue Cancer Prognostication
72. Automatic calibration of arid rainfall-runoff model for wadi thara western Kingdom of Saudi Arabia
73. A Novel Diagnosis Method for Interturn Short-Circuits in SRMs by Tracking Post Turn-Off Phase Currents Under Current Chopping Control
74. Rainwater harvesting and artificial groundwater recharge in arid areas: Case study in Wadi Al-Alb, Saudi Arabia
75. Frequency analysis of rainfall events in Karbala city, Iraq, by creating a proposed formula with eight probability distribution theories
76. Verifying trace inclusion between an experimental frame and a model
77. The Mediterranean Adequacy Index: further confirming results of validity
78. Self-compassion and body dissatisfaction in women: A randomized controlled trial of a brief meditation intervention
79. Bone Marrow Macrophages Contribute to Diabetic Stem Cell Mobilopathy by Producing Oncostatin M
80. Symptom Clusters Change Over Time in Women Receiving Adjuvant Chemotherapy for Breast Cancer
81. Assessment of water harvesting potential sites using GIS-based MCA and a hydrological model: case of Werie catchment, northern Ethiopia
82. Developing a diagnosis-based severity classification system for use in emergency medical services for children
83. Identification of patient groups at highest risk from traditional approach to ovarian cancer treatment
84. Flood simulation to determine flood hazard susceptibility of downstream Singkil watershed in Aceh Province
85. The Self-Compassion Scale-Short Form: Psychometric evaluation in one non-clinical and two clinical Swedish samples
86. Secure Service Provisioning Scheme for Lightweight IoT Devices with a Fair Payment System and an Incentive Mechanism Based on Blockchain
87. The Perception of Secondary School Teachers on the Availability of Counseling Services for Students With Learning Disabilities
88. Unnecessary handover minimization in two-tier heterogeneous networks
89. The co-learning in the design, simulation and optimization of a solar concentrating system
90. Assessment of the SCS-CN initial abstraction ratio for predicting runoff treatment volumes during rain-on-snow events
91. Predicting Solar Proton Events of Solar Cycles 22-24 Using GOES Proton and Soft-X-Ray Flux Features
92. Secure Service Provisioning Scheme for Lightweight Clients with Incentive Mechanism Based on Blockchain
93. Association of genetic variants in STAT5B gene with milk performance and mastitis related traits in dairy cattle
94. Understanding how career identity as a moderator shapes the relationship between career commitment and success: A career construction theory perspective
95. Sustainable service supply chain practices (SSSCPs): a framework development
96. Do any types of double-hybrid models render the correct order of excited state energies in inverted singlet-triplet emitters?
97. Multicentre, clinical trial of burst spinal cord stimulation for neck and upper limb pain NU-BURST: a trial protocol
98. Exploring the relationship between self-compassion and body dysmorphic symptoms in adolescents
99. Enhancing complex system performance using discrete-event simulation
100. Web-Based Health Coaching for Spinal Cord Injury: Results From a Mixed Methods Feasibility Evaluation
101. Image encryption with leveraging blockchain-based optimal deep learning for Secure Disease Detection and Classification in a smart healthcare environment
102. Impact of agronomic practices on physical surface crusts and some soil technical attributes of two winter wheat fields in southern Iraq
103. A use-case approach to the validation of social modeling and simulation
104. Age Inclusive Compassion-Focused Therapy: a Pilot Group Evaluation
105. The Body Compassion Scale: Development and initial validation
106. Targeting body compassion in the treatment of body dissatisfaction: A case study
107. Carbon sequestration in pastures, silvo-pastoral systems and forests in four regions of the Latin American Tropics
108. Abstracts From the First Annual Research Day Hosted by the Michigan State University College of Osteopathic Medicine, Novi, Michigan, May 15, 2023
109. Spinal cord stimulation (SCS) in the treatment of peripheral arterial occlusive disease
110. Evaluating the SWAT model for a low-gradient forested watershed in coastal South Carolina
111. Storm event analysis of four forested catchments on the Atlantic coastal plain using a modified SCS-CN rainfall-runoff model
112. Comparison of mammosphere formation from stem-like cells of normal breast, malignant primary breast tumors, and MCF-7 cell line
113. Simple screening procedure for 72 synthetic cannabinoids in whole blood by liquid chromatographyâ€“tandem mass spectrometry
114. Mechanical testing via dominant shear deformation of small-sized specimen
115. Management of supply chains with attribute-sensitive products: a comprehensive literature review and future research agenda
116. Mindfulness-Based Compassion-Focused Therapy and a Comparison of Effectiveness of Stress Reduction Program on Self-Compassion of HIV Positive Patients
117. Leg symptoms of somatic, psychic, and unexplained origin in the population-based Bonn vein study
118. Multi-classification for EEG motor imagery signals using data evaluation-based auto-selected regularized FBCSP and convolutional neural network
119. Effects of mindful practices on terror of mortality: A randomized controlled trial
120. Assessing dynamic models for high priority waste collection in smart cities
121. Preclinical Efficacy Failure of Human CNS-Derived Stem Cells for Use in the Pathway Study of Cervical Spinal Cord Injury
122. A measure of machismo designed to capture individual differences in Mexican American males
123. Development of Internet Addiction Scale Based on DSM-5 Diagnostic Criteria: An Evaluation in Terms of Internet Gaming Disorder
124. Psychophysiological stress response after a 6-week Mindful Self-Compassion training in psychiatric rehabilitation inpatients: a randomized post-test only study
125. Generating neurons from stem cells
126. Self-compassion and professional quality of life among midwives and nurse assistants: A cross-sectional study
127. A Predictive Score for Secondary Cytoreductive Surgery in Recurrent Ovarian Cancer (SeC-Score): A Single-Centre, Controlled Study for Preoperative Patient Selection
128. Efficient Bidirectional Power Flow Management in EV Powertrain: A Partial Power DC-DC Configuration for HESS Integration
129. Predicting mastitis in dairy cows using neural networks and generalized additive models: A comparison
130. Cost effectiveness of a novel 10 khz high-frequency spinal cord stimulation system in patients with failed back surgery syndrome (FBSS)
131. Hydrological modeling of Hasdeo River Basin using HEC-HMS
132. Reliability of Runoff Hydrograph Model for Extreme Rainfall Events using HEC-RAS 2D Flow Hydrodynamics Rain-On-Grid
133. Behavior of Hollow-Core Steel-Concrete-Steel Columns Subjected to Torsion Loading
134. A literature review: machine learning-based stem cell investigation
135. Preliminary validation study of the intrinsic religious motivation scale and the centrality of religiosity scale for the portuguese population
136. Clarification of issues and long-duration hydrologic simulation SCS-CN-based proxy modelling
137. A Step-Up 5-Level Transformer-Less Switched Capacitor Inverter Without Leakage Current for PV System Application
138. The Compassionate Engagement and Action Scales for Self and Others: Turkish Adaptation, Validity, and Reliability Study
139. Self-compassion and compassion fatigue among licensed mental health clinicians who work with trauma clients
140. Paddle lead placement in the cervical spine region
141. Paddle lead trial in the case of a prior fusion
142. Positive psychology and hope: A cross-cultural test and broadening of Snyder's hope theory in the United States and Mexico
143. A telephone-based version of the spinal cord injury-secondary conditions scale: a reliability and validity study
144. Estimation of runoff for agricultural utilization using geoinformatics: A model study from telangana state
145. The development of Fears of Compassion Scale Japanese version
146. A procedure to estimate the origins and the insertions of the knee ligaments from computed tomography images
147. Relation of the psychological constructs of resilience, mindfulness, and self-compassion on the perception of physical and mental health
148. Effects of compassion meditation on a psychological model of charitable donation
149. Comparative Study of Essential Oils from Different Organs of Syzygium cumini (Pamposia) Based on GC/MS Chemical Profiling and In Vitro Antiaging Activity
150. Changes in the testicular histomorphometry and their association with genes expression pattern of testes from birth to puberty in Beetal goat kids
151. Evaluation of the single compressive strength test in estimating uniaxial compressive and Brazilian tensile strengths and elastic modulus of marlstone
152. Screening borderline personality disorder: The psychometric properties of the Persian version of the McLean screening instrument for borderline personality disorder
153. Beliefs about binge eating: The psychometric properties of the Persian version of the eating beliefs questionnaire
154. The assessment of alexithymia across positive and negative emotions: The psychometric properties of the Iranian version of the perth alexithymia questionnaire
155. Assessment of eating disorder psychopathology: The psychometric properties of the Persian version of the Eating Disorder Examination Questionnaire Short Form
156. Assessment of the emotional reactivity through the positive and negative emotions: The psychometric properties of the persian version of the perth emotional reactivity scale
157. Eating disorders screening tools: The psychometric properties of the persian version of eating attitude test
158. Disordered eating: The psychometric properties of the Persian version of the Eating Attitudes Test-8
159. Self-oriented and Socially Prescribed Perfectionism: The Psychometric Properties of the Persian Version of the Eating Disorder Inventory-Perfectionism Scale
160. RAINFALL-RUNOFF MODELING USING THE HEC-HMS MODEL FOR THE MEKERRA WADI WATERSHED (N-W ALGERIA)
161. Investigating the relationship between self-compassion and occupational stress of nurses working in hospitals affiliated to Tehran university of medical sciences in 2017
162. Engineering the visible light absorption of one-dimensional photonic crystals based on multilayers of Al-doped ZnO (AZO) thin films
163. Narcissism and sensitivity to criticism: A preliminary investigation
164. Distribution and abundance of Heliopora coerulea (Cnidaria: Coenothecalia) and notes on its aggressive behavior against scleractinian corals: Temperature mediated?
165. QSAR-CoMSIA applied to antipsychotic drugs with their dopamine D 2 and serotonine 5HT2A membrane receptors
166. Validation of the Slovene version of The Self-Consciousness Scale
167. Evaluating the impact of preparation conditions and formulation on the accelerated stability of tretinoin loaded liposomes prepared by heating method
168. Interactive effects of market orientation, innovation orientation and sales control systems on firm performance in B2B markets
169. Psychometric Properties of the Mindfulness in Teaching Scale in a Sample of Iranian Teachers: Insight from a Network Analysis Approach
170. Fonksiyonel Olmayan Tutumlar Ã–lÃ§eÄŸi KÄ±sa Formu (FOTÃ–-17) Uyarlama Ã‡alÄ±ÅŸmasÄ± = Turkish adaptation of Dysfunctional Attitude Scaleâ€”Short form (DAS-17)
171. Chemical composition of the essential oils of Citrus sinensis cv. Valencia and a quantitative structure-retention relationship study for the prediction of retention indices by multiple linear regression
172. LARG index: A benchmarking tool for improving the leanness, agility, resilience and greenness of the automotive supply chain
173. Optimal Placement and Sizing of Synchronous Condenser to Enhance System Strength of a Renewable Energy Integrated Weak Grids
174. A new strategy for battery and supercapacitor energy management for an urban electric vehicle
175. Determination of Flash Floods Hazards and Risks for Irbid Governorates Using Hydrological and Hydraulic Modelling
176. [Construction and identification of rhesus monkey Schwann cells modified with human glial cell derived neurotrophic factor gene]
177. Construction and identification of human glial cell-derived neurotrophic factor gene-modified Schwann cells from rhesus monkeys
178. Reward Function Design for Reinforcement Learning based Energy Management Strategy for a Fuel Cell/Supercapacitor Hybrid Electric Vehicle
179. Analytic Performance of a Point-of Care Instrument for Measurement of Human Chorionic Gonadotropin Hormone
180. Mindfulness, cognitive fusion, and self-compassion in patients with schizophrenia spectrum disordersâ€”A cross-sectional study
181. Three-Dimensional Bioprinting in Soft Tissue Engineering for Plastic and Reconstructive Surgery
182. Self-Compassion As A Mediator of The Relationship Between Psychological Inflexibility and Resilience
183. Evaluating Blockchain requirements for effective digital supply chain management
184. Assessment of hydropower potential in Nethravathi river basin using SWAT model
185. A hybrid ANN-MILP model for agile recovery production planning for PPE products under sharp demands
186. Measuring Self-Compassion in Medical Students: Factorial Validation of the Self-Compassion Scale-Short Form (SCS-SF)
187. Meditation and Compassion Therapy in Psychiatric Disorders: A Pilot Study
188. The influence of psychological separation and attachment on the career development of Filipino Americans
189. A reliability generalization meta-analysis of the Self-Compassion Scale original version and its translations and adaptations
190. Does strategic supply chain information system leads to efficient supply chain and improved performance in Indonesian firms
191. Quantitative modeling and analysis of supply chain risks using Bayesian theory
192. Unraveling the Antioxidant Capacity of Spatholobi caulis in Nonalcoholic Fatty Liver Disease: A Multiscale Network Approach Integrated with Experimental Validation
193. Using Self-Report Assessment Methods to Explore Facets of Mindfulness
194. Predictive validity and symptom configuration of proposed diagnostic criteria for the Suicide Crisis Syndrome: A replication study
195. Resilience, self-compassion, and indices of psychological wellbeing: A not so simple set of relationships
196. Navigating circular economy: Unleashing the potential of political and supply chain analytics skills among top supply chain executives for environmental orientation, regenerative supply chain practices, and supply chain viability
197. Measuring self-compassion in people living with dementia: investigating the validity of the Self-Compassion Scale-Short form (SCS-SF)
198. Molecular characterization of complete coding sequence of the MBL1 gene in the Indian Buffalo (Bubalus bubalis) breed
199. Single nucleotide polymorphisms in MBL1 gene of cattle and their association with milk production traits and somatic cell score
200. Radiomics characterization of tissues in an animal brain tumor model imaged using dynamic contrast enhanced (DCE) MRI
201. High energy storage quasi-solid-state supercapacitor enabled by metal chalcogenide nanowires and iron-based nitrogen-doped graphene nanostructures
202. Proteomic analysis of epithelium-denuded human amniotic membrane as a limbal stem cell niche
203. Analytical modeling of oscillatory heat transfer in coated sorption beds
204. Intermittent Generation of Internal Solitary-Like Waves on the Northern Shelf of the South China Sea
205. A UV cross-linked gel polymer electrolyte enabling high-rate and high voltage window for quasi-solid-state supercapacitors
206. Adaptation and validation of a scale for measuring self-compassion in early childhood
207. Evaluating the Big Five as an organizing framework for commonly used psychological trait scales
208. Spinal cord stimulation for treatment of chronic neuropathic pain in adolescent patients: a single-institution series, systematic review, and individual participant data meta-analysis
209. Clinical utility of structural connectomics in predicting memory in temporal lobe epilepsy
210. Novel neural network architecture using sharpened cosine similarity for robust classification of Covid-19, pneumonia and tuberculosis diseases from X-rays
211. Multi step structural health monitoring approaches in debonding assessment in a sandwich honeycomb composite structure using ultrasonic guided waves
212. Construct validity of the Social Styles Scale: A self-report measure of counterdependency
213. Psychometric evaluation of the Spiritual Coping Strategies scale in English, Maltese, back-translation and bilingual versions
214. A new and alternative leads positioning for complex regional pain syndrome treatment: paraforaminal stimulation
215. Assessing the Impact of Land-Use Dynamics to Predict the Changes in Hydrological Variables Using Effective Impervious Area (EIA)
216. Psychological Well-Being of School Counsellors Model
217. A comparative study on different stemness gene expression between dental pulp stem cells vs. dental bud stem cells
218. The elusive S2 state, the S1/S2 splitting, and the excimer states of the benzene dimer
219. Extendable Switched-Capacitor Multilevel Inverter with Reduced Number of Components and Self-Balancing Capacitors
220. Required number of small-cells in heterogenous networks with non-uniform traffic distribution
221. Structural Classes of Dimethylsulfate and Benzonitrile
222. Sedimental pollen records in the northern South China Sea and their paleoenvironmental significance
223. Three-Dimensional Neurite Characterization of Small Incision Lenticule Extraction Derived Lenticules
224. New control approaches for trajectory tracking and motion planning of unmanned tracked robot
225. Distinguishing between privacy and security concerns: An empirical examination and scale validation
226. [Effects of topographic correction on remote sensing estimation of forest biomass]
227. Effects of Harvest Time and Silage on Forage Quality of Sweet Corn Straw Based on Principal Component Analysis and Membership Function Analysis
228. Supercapacitors Fast Ageing Control in Residential Microgrid Based Photovoltaic/Fuel Cell/Electric Vehicle Charging Station
229. Symptom clusters and survival in Portuguese patients with advanced cancer
230. Psychometric properties of the Mindfulness in Teaching Scale in a sample of Portuguese teachers
231. Modeling and parameter identification of lithium-ion capacitor modules
232. Streamflow simulation using Soil and Water Assessment Tool (SWAT): application to Periyar River basin in India
233. A three-factor model of personality predicts changes in depression and subjective well-being following positive psychology interventions
234. Refractory Chronic Pain Screening Tool (RCPST): a feasibility study to assess practicality and validity of identifying potential neurostimulation candidates
235. Utilizing microarray spot characteristics to improve cross-species hybridization results
236. Spanish version of the Interpersonal Mindfulness in Parenting Scale (IM-P)
237. Assessment of near-term risk for suicide attempts using the suicide crisis inventory
238. Body Imageâ€“Acceptance and Action Questionnaireâ€“5: An abbreviation using genetic algorithms
239. Developing the Implicit Relational Assessment Procedure as a measure of self-forgiveness
240. Developing an individualized Implicit Relational Assessment Procedure (IRAP) as a potential measure of self-forgiveness related to negative and positive behavior
241. The Implicit Relational Assessment Procedure (IRAP) as a measure of self-forgiveness: The impact of a training history in clinical behavior analysis
242. The Influence of Protein Stability on Sequence Evolution: Applications to Phylogenetic Inference
243. Potential Effects of Forest Fires on Streamflow in the Enipeas River Basin, Thessaly, Greece
244. Selfâ€compassion and emotional regulation as predictors of social anxiety
245. Molecularly imprinted polymer based quartz crystal microbalance sensor system for sensitive and label-free detection of synthetic cannabinoids in urine
246. Estimation of the 10-year risk of cardiovascular diseases: Using the SCORE, WHO/ISH, and framingham models in the shahrekord cohort study in southwestern Iran
247. Simulation of flood event in basin scale using HECHMS and GIS
248. Presenting a Method to Improve Bone Quality Through Stimulation of Osteoporotic Mesenchymal Stem Cells by Low-Level Laser Therapy
249. Development and validation of the Body Compassion Questionnaire
250. The role of self-compassion in the well-being of self-identifying gay men
251. Measuring relationships between selfâ€compassion, compassion fatigue, burnout and wellâ€being in student counsellors and student cognitive behavioural psychotherapists: A quantitative survey
252. Relationships of masculinity and ethnicity as at-risk markers for online sexual addiction in men
253. Efficient startâ€“up energy management via nonlinear control for ecoâ€“traction systems
254. A Blockchain Framework for Containerized Food Supply Chains
255. Validity and reliability: The psychometric properties of the Persian version of Short Form of the Impulsiveness Questionnaire UPPS-P in Iran
256. Parallel nine-phase generator control in a medium-voltage DC wind system
257. Reliability and validity of the Suicide Cognitions Scale-Revised (SCS-R) in emerging adulthood in Turkey
258. Comparison of HEC-HMS hydrologic model for estimation of runoff computation techniques as a design input: case of Middle Awash multi-purpose dam, Ethiopia
259. Corrigendum: Eating disorder symptoms and proneness in gay men, lesbian women, and transgender and gender non-conforming adults: Comparative levels and a proposed mediational model [Front. Psychol. 9, (2019) (2692)] doi: 10.3389/fpsyg.2018.02692
260. Eating disorder symptoms and proneness in gay men, lesbian women, and transgender and non-conforming adults: Comparative levels and a proposed mediational model
261. 'Eating disorder symptoms and proneness in gay men, lesbian women, and transgender and non-conforming adults: Comparative levels and a proposed mediational model': Corrigendum
262. Unpaired Image Captioning with semantic-Constrained Self-Learning
263. Differences in self-compassion and shame in patients with anxiety disorders, patients with depressive disorders and healthy controls
264. Physicochemical and sensory properties of grape juices produced from different cultivars and extraction systems
265. Resolution of lateral acoustic space assessed by electroencephalography and psychoacoustics
266. Embankment dams in earth fissure risk zones - A regulator's dilemma
267. The mediating effects of mindfulness and self-compassion on trait anxiety
268. Evaluation of Changes in Quality Improvement Knowledge Following a Formal Educational Curriculum Within a Statewide Learning Collaborative
269. Assessing psychological inflexibility in text: An examination of the inflexitext program
270. Measuring dispositional self-control capacity. A German adaptation of the short form of the Self-Control Scale (SCS-K-D)
271. Messung dispositioneller selbstkontroll-kapazitÃ¤t Eine deutsche adaptation der Kurzform der Self-Control Scale (SCS-K-D) = Measuring dispositional self-control capacity A German adaptation of the short form of the Self-Control Scale (SCS-K-D)
272. Building blocks of emotional flexibility: Trait mindfulness and self-compassion are associated with positive and negative mood shifts
273. Stem cell dynamics and cellular heterogeneity across lineage subtypes of castrate-resistant prostate cancer
274. Understanding gender differences in traditional and cyberbullying: An evaluation of construct validity of the 2013 school crime supplement to the national crime victimization survey
275. A lesionâ€aware automated processing framework for clinical stroke magnetic resonance imaging
276. Rainfall-runoff modeling: Comparison of two approaches with different data requirements
277. Single Phase Five Level Step-Up Switched Capacitor Based Grid Connected Inverter with LCL Filter
278. Development of a diffusive wave flood routing model for an ungauged basin: a case study in Kulsi River Basin, India
279. Numerical Approach for Channel Flood Routing in an Ungauged Basin: a Case Study in Kulsi River Basin, India
280. An approach for prediction of flood hydrograph at outlet of an ungauged basin using modified dynamic wave model
281. Synthesis and experimental investigation of zinc oxide and praseodymium oxide fused metal oxide nanostructures
282. The role of hox genes in regulating stem cell population in normal and malignant colon tissue
283. Identification of a developmental gene expression signature, including HOX genes, for the normal human colonic crypt stem cell niche: overexpression of the signature parallels stem cell overpopulation during colon tumorigenesis
284. Switched capacitors 9-level module (SC9LM) with reduced device count for multilevel DC to AC power conversion
285. Using fuzzy systems for optimal network reconfiguration of a distribution system with electric vehicle charging stations and renewable generation
286. Blockchain-Driven andÂ IoT-Assisted Chemical Supply-Chain Management
287. Proteasomal pathway inhibition as a potential therapy for NF2-associated meningioma and schwannoma
288. Event-based rainfallâ€“run-off modeling and uncertainty analysis for lower Tapi Basin, India
289. An integrated grey-based multi-criteria optimisation approach for sustainable supplier selection and procurement-distribution planning
290. Curve-folded form-work for cast, compressive skeletons
291. Suitability of Gamma, Chi-square, Weibull, and Beta distributions as synthetic unit hydrographs
292. Common grounded H-type bidirectional DC-DC converter with a wide voltage conversion ratio for a hybrid energy storage system
293. Development and validation of mechanical properties models for DP steels
294. Protection versus risk? The relative roles of compassionate and uncompassionate self-responding for eating disorder behaviors
295. Clarifying the Relationship Between Self-Compassion and Mindfulness: An Ecological Momentary Assessment Study
296. Psychometric properties of the German version of the Forms of Self-Criticizing/Attacking and Self-Reassuring Scale (FSCRS)
297. Psychometric properties of the German version of the fears of compassion scales
298. Basic dimensions of resilient coping in paramedics and dispatchers
299. Modelling changing catchment under the climate variability: A case study from a semi-arid catchment in the upper basin of the Goulburn River
300. A Global map of rainfed cropland areas at the end of last millennium using remote sensing and geospatial techniques
301. Metabolomic Applications in Stem Cell Research: a Review
302. Impact of somatic cell count combined with differential somatic cell count on milk protein fractions in Holstein cattle
303. A High Performance Switched-Capacitor Based Single-Phase Transformerless Inverter
304. Dynamic Changes in Nociception and Pain Perception After Spinal Cord Stimulation in Chronic Neuropathic Pain Patients
305. Invited Commentary: 30-Year Perspective on the Seven Countries Study
306. The revised suicide crisis inventory (SCI-2): Validation and assessment of prospective suicidal outcomes at one month follow-up
307. The suicide crisis syndrome: A network analysis
308. Associations Between Pre-Implant Psychosocial Factors and Spinal Cord Stimulation Outcome: Evaluation Using the MMPI-2-RF
309. Mindfulness and self-compassion: Exploring pathways to adolescent emotional well-being
310. Erratum to: 'Mindfulness and self-compassion: Exploring pathways to adolescent emotional well-being'
311. Investigating the Impact of Mindfulness Meditation on Stress Reduction and Self-Compassion of Nursing Health Care Professionals in a Small Community Hospital in the Midwest: A Pilot Study
312. Microstructural changes and in-situ observation of localization in OFHC copper under dynamic loading
313. The role of mindfulness and loving-kindness meditation in cultivating self-compassion and other-focused concern in health care professionals
314. Prediction accuracies and genetic parameters for test-day traits from genomic and pedigree-based random regression models with or without heat stress interactions
315. Modelling the event-based hydrological response of olive groves on steep slopes and clayey soils under mulching and tillage management using the SCS-CN, Horton and USLE-family models
316. Appraisal of applicants for ministry careers
317. Testing direct and indirect ties of selfâ€‘compassion with subjective wellâ€‘being
318. The influence of calf rearing methods and milking methods on performance traits of crossbred dairy cattle in Thailand - 1. Milk yield and udder health
319. Coal-based graphitized activated carbon for solar energy powered supercapacitor IoT applications
320. Eliminating Senescent Cells Can Promote Pulmonary Hypertension Development and Progression
321. Recruitment mechanisms and effectiveness of the federal administrative elite
322. Relationship of total bacterial and somatic cell counts with milk production and composition â€“ Multivariate analysis
323. Toward component-based self-adaptive multi-strategic pedagogical agents
324. How integrated are behavioral and endocrine stress response traits? A repeated measures approach to testing the stress-coping style model
325. Post-interdisciplinary frames of reference: exploring permeability and perceptions of disciplinarity in the social sciences
326. Improvements in emotion regulation following mindfulness meditation: Effects on depressive symptoms and perceived stress in younger breast cancer survivors
327. Self-criticism, self-compassion, and perceived health: Moderating effect of ethnicity
328. Defining and evaluating heat stress thresholds in different dairy cow production systems
329. Human platelet lysate stimulates neurotrophic properties of human adipose-derived stem cells better than Schwann cell-like cells
330. Data analysis software package for radionuclide standardization with a digital coincidence counting system
331. CD4+ immune response as a potential biomarker of patient reported inflammatory bowel disease (IBD) activity
332. Self-compassion in old age: confirmatory factor analysis of the 6-factor model and the internal consistency of the Self-compassion scale-short form
333. Internalized weight stigma and intuitive eating among stressed adults during a mindful yoga intervention: Associations with changes in mindfulness and self-compassion
334. Self-compassion, body image, and self-reported disordered eating
335. Do self-compassion and self-coldness distinctly relate to distress and well-being? A theoretical model of self-relating
336. Self-evaluation questionnaires: Rapid user-friendly screening tools the neurologist can use for sexual disorders
337. Acceptability, feasibility and preliminary efficacy of a compassionâ€based cognitive behavioural intervention for low selfâ€esteem in sexual minority young adults
338. Comparing perceptions about collaborative culture from certified and non-certified staff members through the adaptation of the school culture survey-teacher form
339. Development and preliminary evaluation of the Peace Attitudes Scale
340. The relationship of self-compassion with perfectionistic self-presentation, perceived forgiveness, and perceived social support in an undergraduate Christian community
341. Social support and subjective health in fibromyalgia: Self-compassion as a mediator
342. Validation of the Compassionate Engagement and Action Scales, Compassion Scale, and Sussex-Oxford Compassion Scales in a French-Canadian sample
343. Self-compassion as a moderating factor in the relation of body-image to depression and self-esteem
344. A novel objective chute score interacts with monensin to affect growth of receiving cattle
345. Improving the detection and prediction of suicidal behavior among military personnel by measuring suicidal beliefs: an evaluation of the Suicide Cognitions Scale
346. The structure of suicidal beliefs: A Bifactor analysis of the Suicide Cognitions Scale
347. A Shortened Version of the Suicide Cognitions Scale for Identifying Chronic Pain Patients at Risk for Suicide
348. Psychometric evaluation of the Suicide Cognitions Scale-Revised (SCS-R)
349. Prospective Validity of the Suicide Cognitions Scale Among Acutely Suicidal Military Personnel Seeking Unscheduled Psychiatric Intervention
350. Analysis of substructural variation in families of enzymatic proteins with applications to protein function prediction
351. A low-cost â€œwater-in-saltâ€ electrolyte for a 2.3 V high-rate carbon-based supercapacitor
352. All-climate aqueous supercapacitor enabled by a deep eutectic solvent electrolyte based on salt hydrate
353. Genome-wide association study on milk production and somatic cell score for Thai dairy cattle using weighted single-step approach with random regression test-day model
354. Genomic prediction of milk-production traits and somatic cell score using single-step genomic best linear unbiased predictor with random regression test-day model in Thai dairy cattle
355. Mental health stigma and help seeking among college students
356. Item Wording Effects in Psychological Measures: Do Early Literacy Skills Matter?
357. Rationale, methods, feasibility, and preliminary outcomes of a transdiagnostic prevention program for at-risk college students
358. Initial Psychometric Validation of the Non-Suicidal Self-Injury Scar Cognition Scale
359. Skin Carotenoid Status Over Time and Differences by Age and Sex Among Head Start Children (3-5 Years)
360. Optimization of Ex Vivo Machine Perfusion and Transplantation of Vascularized Composite Allografts
361. Sexual Sensation Seeking, Sexual Compulsivity, and Gender Identity and Its Relationship With Sexual Functioning in a Population Sample of Men and Women
362. Preliminary Validation of a German Version of the Sexual Complaints Screener for Women in a Female Population Sample
363. Validation of the food craving Acceptance and action questionnaire (FAAQ) in a weight loss-seeking sample
364. The role of body compassion in the risk of eating disorders: Mediational effects of body appreciation and body shame
365. Uniqueness and status consumption in Generation Y consumers: Does moderation exist?
366. Using Transfer Learning to Train a Binary Classifier for Lorrca Ektacytometery Microscopic Images of Sickle Cells and Healthy Red Blood Cells
367. Validity evidence of the Spanish version of the Mindful Attention Awareness Scale using the Rasch measurement model
368. Uncertainties and errors flow estimate using hydrological modelling and precipitation by RADAR
369. Design and simulation studies of battery-supercapacitor hybrid energy storage system for improved performances of traction system of solar vehicle
370. Battery and supercapacitor for photovoltaic energy storage: A fuzzy logic management
371. Extreme Event-based Rainfall-runoff Simulation Utilizing GIS Techniques in Irawan Watershed, Palawan, Philippines
372. Off-Chain Execution of IoT Smart Contracts
373. Lactobacillus by-products inhibit the growth and virulence of uropathogenic Escherichia coli
374. Lactobacillus by-products inhibit the growth and virulence of uropathogenic Escherichia coli
375. The Impact of Near Sourcing on Global Dynamic Supply Chains: A Case Study
376. The role of performance measurement systems to support quality improvement initiatives at supply chain level
377. Supply chain coordination with strategic customers: Yield uncertainty and replenishment tactic
378. Simulation and Optimization Strategy of Storm Flood Safety Pattern Based on SCS-CN Model
379. Model test and simulation comparison for an inclined-leg TLP dedicated to floating wind
380. Improving direct runoff estimations through modifying SCS-CN initial abstraction ratio in a catchment prone to flash floods
381. Improvement of SCS-CN initial abstraction coefficient in the Czech Republic: A study of five catchments
382. Partial to Full Composite Action in Steelâ€“Concrete Sandwich Beams: Development of a Modeling Strategy and Comparison to Standards
383. Refined and Simplified Simulations for Steelâ€“Concreteâ€“Steel Structures
384. The Self-Acceptance of Sexuality Inventory (SASI): Development and validation
385. Canonical Notch signaling plays an instructive role in auditory supporting cell development
386. Meditation and happiness: Mindfulness and self-compassion may mediate the meditationâ€“happiness relationship
387. Myogenic exosome miR-140-5p modulates skeletal muscle regeneration and injury repair by regulating muscle satellite cells
388. Äá»i Cha Ä‚n Máº·n, Äá»i Con KhÃ¡t NÆ°á»›c: Perceptions of intergenerational trauma and parenting styles on self-compassion in adult children of Vietnamese refugees
389. Social connectedness as resource of resilience: Italian validation of the Social Connectedness Scaleâ€”Revised
390. The Early Memories of Warmth and Safeness Scale: Dimensionality and measurement invariance
391. Use of Accelerometry as an Educational Tool for Spinal Cord Stimulation: A Pilot Study
392. Self-change strategies in smokers and former smokers: Spanish adaptation of the SCS-CS and SCS-FS
393. Behavioral and physiological responses to stocking density in sea bream (Sparus aurata): Do coping styles matter?
394. Cross-cultural adaptation and validation of the Swallowing Disturbance Questionnaire and the Sialorrhea Clinical Scale in Portuguese patients with Parkinson's disease
395. COVID-19 Impacts and Mitigation Strategies on Food Supply Chains: A Survey to the Brazilian Context
396. Skeletal muscle tissue engineering: Biomaterials-based strategies for the treatment of volumetric muscle loss
397. Modelling the event-based hydrological response of mediterranean forests to prescribed fire and soil mulching with fern using the curve number, horton and USLE-Family (universal soil loss equation) models
398. Measuring self-disgust in adolescence: Adaptation and validation of a new instrument for the Portuguese adolescent population
399. Validation of the Borderline Personality Features Scale for Children (BPFS-C) and for Parents (BPFS-P) for the Portuguese population
400. Construct validity of the Smoker Complaint Scale: A clinimetric analysis using Item Response Theory (IRT) models
401. The relationship between self-efficacy, self-compassion, and anger among evangelical men ages thirty to forty
402. Flood modelling in a high country mountain catchment, New Zealand: comparing statistical and deterministic model estimates for ecological flows
403. The utility of the Valuing Questionnaire in Chronic Pain
404. Time-Dependent Long-Range-Corrected Double-Hybrid Density Functionals with Spin-Component and Spin-Opposite Scaling: A Comprehensive Analysis of Singlet-Singlet and Singlet-Triplet Excitation Energies
405. Performance model for two-tier mobile wireless networks with macrocells and small cells
406. Bowel scintigraphy identifies segmental dysmotility prior to stoma closure
407. Academic expectations and well-being in school children
408. Effect of packaging on supercapacitors strings modeling: Proposal of functional unit defined around balancing circuit
409. Modelling, Simulation and Characterization of a Supercapacitor in Automotive Applications
410. Modeling, Simulation, and Characterization of a Supercapacitor in Automotive Applications
411. NMR Metabolomics for Stem Cell type discrimination
412. Exploring selfâ€criticism: Confirmatory factor analysis of the FSCRS in clinical and nonclinical samples
413. Interaction of seed treatment and herbicide tank mixture in paddy rice selectivity
414. Candidate gene association analysis for milk yield, composition, urea nitrogen and somatic cell scores in Brown Swiss cows
415. Minimally-Invasive Secondary Cytoreduction in Recurrent Ovarian Cancer
416. A metamodel and a DEVS implementation for component based hierarchical simulation modeling
417. What do (and donâ€™t) we know about self-compassion? Trends and issues in theory, mechanisms, and outcomes
418. Role of superconducting energy gap in extended BCS-Bose crossover theory
419. Vibration Analysis of Composite Laminated and Sandwich Conical Shell Structures: Numerical and Experimental Investigation
420. Comparison of infiltration models to simulate flood events at the field scale
421. [Evaluation of taste changes of Scutellariae Radix before and after wine-frying based on electronic ongue technology and its application in identification of Scutellariae Radix pieces]
422. Study on the Role and Mechanism of Exosomes Derived from Dental Pulp Stem Cells in Promoting Regeneration of Myelin Sheath in Rats with Sciatic Nerve Injury
423. The Rain-on-Grid Modeling Approach in Hydrological and Hydraulic Processes over a River Basin
424. Construction and Statistical Validation of a Path Model of the Factors Influencing Non-suicidal Self Injury (NSSI) among Female Adolescents
425. Simulation of flow at an ungauged river site based on HEC-HMS model for a mountainous river basin
426. Burst Spinal Cord Stimulation: A Systematic Review and Pooled Analysis of Real-World Evidence and Outcomes Data
427. Transfection of Trypanosoma cruzi with host CD40 ligand results in improved control of parasite infection
428. Guided implant surgery with R2GateÂ®: A multicenter retrospective clinical study with 1 year of follow-up
429. Guided implant surgery with R2Gate R: A multicenter retrospective clinical study with 1 year of follow-up
430. Ultrafine nanoparticles of tin-cobalt-sulfide decorated over 2D MXene sheets as a cathode material for high-performance asymmetric supercapacitor
431. Predictors of posttraumatic growth of intensive care unit nurses in Korea
432. Aminosalicylic acid reduces ER stress and Schwann cell death induced by MPZ mutations
433. Cross-cultural comparison of compassion: An in-depth analysis of cultural differences in compassion using the Compassion of Othersâ€™ Lives (COOL) Scale
434. Offshore wind resources assessment from multiple satellite data and WRF modeling over South China Sea
435. Seed selection and social coupon allocation for redemption maximization in online social networks
436. Lowest-vINCMOS Single-Inductor Boost Charger: Design, Limits, and Validation
437. Adaptive Cost-Reward Scheduling for Optimizing Radio Utilization and NR Numerology Efficiency in B5G New Radio Networking
438. Effects of switching costs on customer loyalty in the liner shipping industry
439. The mediating role of emotion regulation difficulties in the relationship between self-compassion and OCD severity in a non-referred sample
440. Supply chain sustainability during an uncertain environment: examining the moderating role of leadership support
441. Emotional recovery following divorce: Will the real self-compassion please stand up?
442. A Malmquist fuzzy data envelopment analysis model for performance evaluation of rural healthcare systems
443. PCA driven watershed prioritization based on runoff modeling and drought severity assessment in parts of Koel river basin, Jharkhand (India)
444. Myelin-Associated Glycoprotein Inhibits Schwann Cell Migration and Induces Their Death
445. Novel Bidirectional DC-DC Converter with Battery Temperature Modulation Capability using High Frequency Resonant Network
446. Geospatial modelling of watershed peak flood discharge in Selangor, Malaysia
447. Optimal Size and Location of Batteries for Frequency Support for SÃ£o Vicente Island's System
448. The tumor suppressor effect of the glucocorticoid receptor in skin is mediated via its effect on follicular epithelial stem cells
449. An improved microgrid energy management system based on hybrid energy storage system using ANN NARMA-L2 controller
450. The validation of the significant wave height product of HY-2 altimeter-primary results
451. A mathematical model for determining the pose of a SLDV
452. Implanted synthetic cells trigger tissue angiogenesis through de novo production of recombinant growth factors
453. Predicting social cues during online discussions: Effects of evaluations and knowledge content
454. Perceived parenting styles and body appreciation among Chinese adolescents: Exploring the mediating roles of dispositional mindfulness and self-compassion
455. An elastoplastic energy model for predicting the deformation behaviors of various structural components
456. Constructing a Flood-Adaptive Ecological Security Pattern from the Perspective of Ecological Resilience: A Case Study of the Main Urban Area in Wuhan
457. Simulating the hydrologic impacts of land-cover and climate changes in a semi-arid watershed
458. Comparison of nonlinear and linear PCA on surface wind, surface height, and SST in the South China Sea
459. Constructing a Flood-Adaptive Ecological Security Pattern from the Perspective of Ecological Resilience: A Case Study of the Main Urban Area in Wuhan
460. Spatial distribution, homologue patterns and ecological risks of chlorinated paraffins in mangrove sediments along the South China Coast
461. Implementation of a non-contact X-Y mover with high temperature superconductors
462. Relationship over southern China between the summer rainfall induced by tropical cyclones and that by monsoon
463. On the Decentralized Energy Management Strategy for the All-Electric APU of Future More Electric Aircraft Composed of Multiple Fuel Cells and Supercapacitors
464. Psychometric Properties of the Short-form Version of the Cognitive Style Questionnaire
465. Promotive effect of skin precursor-derived Schwann cells on brachial plexus neurotomy and motor neuron damage repair through milieu-regulating secretome
466. Impact of genotype imputation on the performance of GBLUP and Bayesian methods for genomic prediction
467. Optimal transmission frequency selection base on scattering cross section for ultrasound contrast harmonic imaging
468. Lithospheric structure of the South China Sea and adjacent regions: Results from potential field modelling
469. The Experienced Benefits of the 17-Item Benefit Finding Scale in Chinese Colorectal Cancer Survivor and Spousal Caregiver Couples
470. A method for urban flood risk assessment and zoning considering road environments and terrain
471. A mathematical programming approach to supply chain models with fuzzy parameters
472. Mantle sources of Cenozoic volcanoes around the South China Sea revealed by geochemical and isotopic data using the principal component analysis
473. Preparation of poly(methacrylic acid-co-ethylene glycol dimethacrylate)-functionalized magnetic polydopamine nanoparticles for the extraction of six cannabinoids in wastewater followed by UHPLC-MS/MS
474. Preceptors' Subjective Competency Ratings in Acute Care Hospitals in Taiwan
475. Modelling and verification of an automatic controller for a water treatment mixing tank
476. Spatial Distribution Recast for Organic Bulk Heterojunctions for High-Performance All-Inorganic Perovskite/Organic Integrated Solar Cells
477. Ocean Turbulence Denoising and Analysis Using a Novel EMD-Based Denoising Method
478. Denoising Ocean Turbulence Microstructure Signals for Application in Estimating Turbulence Kinetic Energy Dissipation Rates Based on EMD-PCA
479. Automatic extraction of subordinate clauses and its application in second language acquisition research
480. Mediating effect of self-concealment between non-suicidal self-injury and internet addiction in college students: a cross-sectional study
481. Scattering Center Modeling of Typical Planetary Landforms for Planetary Geomorphologic Exploration
482. Multiband acoustic helical interface states in inverse-designed sonic crystals with glide symmetry
483. Scattering center modeling for low-detectable targets
484. 3D nanotube-structured Ni@MnO2 electrodes: Toward enhanced areal capacitance of planar supercapacitors
485. Design of a Low-Profile and Low Scattering Wideband Planar Phased Antenna Array
486. PI3K/Akt signaling pathway is essential for de novo hair follicle regeneration
487. Runoff estimation in hillslope cropland of purple soil based on SCS-CN model
488. Reliable and Efficient Sparse Code Spreading Aided MC-DCSK Transceiver Design for Multiuser Transmissions
489. Experimental and numerical study on damage behavior of air-backed steel-concrete-steel composite panels subjected to underwater contact explosion
490. Co-transduction of dual-adeno-associated virus vectors in the neonatal and adult mouse utricles
491. RobustClone: a robust PCA method for tumor clone and evolution inference from single-cell sequencing data
492. RobustTree: An adaptive, robust PCA algorithm for embedded tree structure recovery from single-cell sequencing data
493. Age-related transcriptome changes in Sox2+ supporting cells in the mouse cochlea
494. Editable asymmetric all-solid-state supercapacitors based on high-strength, flexible, and programmable 2D-metal-organic framework/reduced graphene oxide self-assembled papers
495. Asymptotically optimal 2 Â¯ -separable codes with length 4
496. ENHANCING RAINFALL-RUNOFF POLLUTION MODELING BY INCORPORATION OF NEGLECTED PHYSICAL PROCESSES
497. Fine root densities of grasses and perennial sugarcane significantly reduce stream channel erosion in southern China
498. Effects of a group-based music imagery program on promoting coping resources among undergraduate students: a pilot randomized controlled trial
499. Sea-level trend in the South China Sea observed from 20 years of along-track satellite altimetric data
500. An Improved Remote Sensing Retrieval Method for Elevated Duct in the South China Sea
501. Validation of a Chinese Short Version of the Kentucky Inventory of Mindfulness Skills (KIMS-17) among people recovering from mental illness
502. Precise management system for chronic intractable pain patients implanted with spinal cord stimulation based on a remote programming platform: study protocol for a randomized controlled trial (PreMaSy study)
503. Mindfulness, and specifically compassion, buffers the relationship between hopelessness and suicide ideation in college students
504. A comprehensive review on application of stem cells for kidney diseases
505. A multiple indicator multiple cause (mimic) model of the self-compassion scale youth (scs-y) and investigation of differential item functioning in china, hong kong and uk adolescents
506. Stem cell/cellular interventions in human spinal cord injury: Is it time to move from guidelines to regulations and legislations? Literature review and Spinal Cord Society position statement
507. Development and validation of a simplified thoracolumbar spine fracture classification system
508. Tropical cyclone rainfall structure affecting indochina peninsula and lower mekong river basin (LMB)
509. Psychometric evaluation of the Fear of COVID-19 Scale among Chinese population
510. Multidecadal changes of Upper-ocean thermal conditions in the tropical northwest Pacific Ocean versus South China Sea during 1960-2015
511. Characterization of Spo11-dependent and independent phospho-H2AX foci during meiotic prophase I in the male mouse
512. Conjugation of synthetic cannabinoids JWH-018 and JWH-073, metabolites by human UDP-glucuronosyltransferases
513. Deformed Gazeau-Klauder SchrÃ¶dinger cat states with modified commutation relations
514. Development and preliminary validation of a self-compassion measure
515. Development and preliminary validation of the Brief Self-Compassion Inventory
516. Influence of DEM resolution on topographic correction models using spot satellite image
517. Control strategies of the variable speed generating systems
518. The development and validation of the Lovingkindness-Compassion Scale
519. Application of NEXRAD radar-based quantitative precipitation estimations for hydrologic simulation using arcpy and HEC software
520. NEXRAD quantitative precipitation estimations for hydrologic simulation using a hybrid hydrologic model
521. A size-exclusion HPLC method for the determination of sodium chondroitin sulfate in pharmaceutical preparations
522. Proposal of Pseudo-Capping Effects by Polymerâ€“Polymer Interaction through Preparation of Stereocomplex Pseudo-Polyrotaxane with Cyclodextrin and PEG-PLA Triblock Copolymers
523. An inflammatory gene signature distinguishes neurofibroma Schwann cells and macrophages from cells in the normal peripheral nervous system
524. Simulating the effects of agricultural management on water quality dynamics in rice paddies for sustainable rice production-model development and validation
525. Variability in Irradiance and Photometric Indices During the Last Two Solar Cycles
526. Runoff simulation using distributed hydrological modeling approach, remote sensing and GIS techniques: A case study from an Indian agricultural watershed
527. Advanced Switching Sequences Based Model-Predictive Control for Single-Phase NPC Converters
528. Assessing strategies to mitigate the impacts of a pandemic in apparel supply chains
529. Implementing smart waste management system for a sustainable circular economy in the textile industry
530. A test of the multidimensionality of the Self-Construal Scale in Thailand and the United States
531. Effect of compassion fatigue on emotional labor in female nurses: Moderating effect of self-compassion
532. South China Sea wind-wave characteristics. Part 1: Validation of wavematch-III using TOPEX/Poseidon data
533. Validation of Wavewatch-III Using the TOPEX/POSEIDON Data
534. Theory-based SCS-CN method and its applications
535. On the decomposition of cell phone activity patterns and their connection with urban ecology
536. Intramolecular spin alignment in photomagnetic molecular devices: a theoretical study
537. A Semiautonomous Control Strategy Based on Computer Vision for a Handâ€“Wrist Prosthesis
538. Validation of the Dutch Comprehensive Inventory of Mindfulness Experiences (CHIME) and development of a short form (CHIME-SF)
539. Emotional labor and emotional exhaustion in psychologists: Preliminary evidence for the protective role of self-compassion and psychological flexibility
540. Scientific caricatures in the earth science classroom: An alternative assessment for meaningful science learning
541. The Suicidal Narrative and Its Relationship to the Suicide Crisis Syndrome and Recent Suicidal Behavior
542. Introducing the GASP scale: A new measure of guilt and shame proneness
543. Transcriptomic profile reveals deregulation of hearing-loss related genes in vestibular schwannoma cells following electromagnetic field exposure
544. A new simplified comorbidity score as a prognostic factor in non-small-cell lung cancer patients: description and comparison with the Charlson's index
545. Seismic waveform inversion for core-mantle boundary topography
546. Teaching MDE through the formal verification of process models
547. Hydraulic geometry, GIS and remote sensing, techniques against rainfall-runoff models for estimating flood magnitude in ephemeral fluvial systems
548. Validation and psychometric evaluation of the Italian version of the Spinal Cord Injury Secondary Conditions Scale
549. Holocene changes in biome size and tropical cyclone activity around the Northern South China Sea
550. The role of internal resources in academic achievement: Exploring the meaning of self-compassion in the adaptive functioning of low-income college students
551. Timber in design-build architecture education
552. Hybrid powertrain, energy management system and techno-economic assessment of rubber tyre gantry crane powered by diesel-electric generator and supercapacitor energy storage system
553. Sonosensitive capsules for brain thrombolysis increase ischemic damage in a stroke model
554. The creativity-spirituality construct and its role in transformative coping
555. Muscle Microbiopsy to Delineate Stem Cell Involvement in Young Patients: A Novel Approach for Children With Cerebral Palsy
556. Generation of Bidimensional and Three-Dimensional Muscle Culture Systems
557. Genetic association between somatic cell score and milk lactose in early-to mid-lactation of first calving fleckvieh cows
558. Acceptance of pain, selfâ€compassion and psychopathology: Using the Chronic Pain Acceptance Questionnaire to identify patients' subgroups
559. Evidence for the involvement of the anthranilate degradation pathway in Pseudomonas aeruginosa biofilm formation
560. Anomalous diffusion and dynamical correlation between the side chains and the main chain of proteins in their native state
561. Joint receiver function/ScS reverberation analysis for examining discontinuity structure beneath Ocean Islands
562. I can see (myself) clearly now: Exploring the mediating role of self-concept clarity in the association between self-compassion and indicators of well-being
563. Building a forward-mode three-dimensional reflectance model for topographic normalization of high-resolution (1-5 m) imagery: Validation phase in a forested environment
564. A comparative analysis of prediction models for complete gross resection in secondary cytoreductive surgery for ovarian cancer
565. Simple components
566. Systematic symptom and problem assessment at admission to the palliative care ward - perspectives and prognostic impacts
567. Brain responses to a self-compassion induction in trauma survivors with and without post-traumatic stress disorder
568. Exploring self-compassion with lesbian, gay, and bisexual persons
569. Healing the self: The role of self-compassion and empathy in a mindfulness based modality for women survivors of interpersonal violence
570. Serious games for first person 'thinker'
571. VALIDITY AND RELIABILITY OF THE SPIRITUAL COPING STRATEGIES SCALE ARABIC VERSION IN SAUDI PATIENTS UNDERGOING HAEMODIALYSIS
572. Psychometric Evaluation of the Spiritual Coping Strategies Scale Arabic Version for Saudi Nursing Students
573. Psychometric Evaluation of the Filipino Versions of the Duke University Religion Index and the Spiritual Coping Strategies Scale in Filipino Hemodialysis Patients
574. Cogeneration systems of solar energy integrated with compressed air energy storage systems: A comparative study of various energy recovery strategies
575. Vertical distribution of archaeal communities in cold seep sediments from the Jiulong methane reef area in the South China Sea
576. Wave Height Estimation and Validation Based on the UFS Mode Data of Gaofen-3 in South China Sea
577. Satellite views of the seasonal and interannual variations of the particulate organic carbon in the northern south china sea
578. A Modified Smoothed Finite Element Method for Static and Free Vibration Analysis of Solid Mechanics
579. Assessing positive emotional memories with peers: The Early Memories of Warmth and Safeness with Peers Scale for adolescents
580. The flows of compassion in adolescents as measured by the compassionate engagement and action scales
581. The forms of Self-Criticising & Self-Reassuring Scale - Short Form for adolescents: Psychometric properties in clinical and non-clinical Portuguese samples
582. Relationship between self-concept and self-compassion scores and weight-loss surgery outcomes in women
583. Improving Limb Salvage for Chronic Limb-Threatening Ischemia With Spinal Cord Stimulation: A Retrospective Analysis
584. Measuring Readiness to Change Among Individuals in Residential Therapeutic Community Programs for Treatment of Substance Abuse
585. Fuel cell/supercapacitor passive configuration sizing approach for vehicular applications
586. Evaluation of the Effects of High Intensity Interval Training on Cytokine Levels and Clinical Course in Treatment of Opioid Use Disorder
587. Development and validation of a solid phase extraction-HPLC method for the determination of carbamazepin and its metabolites, carbamazepin epoxide and carbamazepin trans-diol, in plasma
588. Inferring individual cow effects, dairy system effects and feeding effects on latent variables underlying milk protein composition and cheese-making traits in dairy cattle
589. Biomechanical properties of fixed-angle volar distal radius plates under dynamic loading
590. Mindfulness, selfâ€compassion, posttraumatic stress disorder symptoms, and functional disability in US Iraq and Afghanistan war veterans
591. Blockchain-Enabled Cyber-Resilience Enhancement Framework of Microgrid Distributed Secondary Control Against False Data Injection Attacks
592. Voltage Fluctuation in a Supercapacitor During a High-g Impact
593. Electrospun Composite PLLA-PPSB Nanofiber Nerve Conduits for Peripheral Nerve Defects Repair and Regeneration
594. Three-Dimensional Modeling of Tsunami Waves Triggered by Submarine Landslides Based on the Smoothed Particle Hydrodynamics Method
595. Three-Phase Switched-Capacitor Boost Self-Balanced Multilevel Inverter for Photovoltaic Applications
596. Stroke Communication Scale based on the International Classification of Functioning, Disability and Health (ICF - SCS)
597. Dynamic models of the complex microbial metapopulation of lake mendota
598. The Italian validation of singelis's Self-Construal Scale (SCS): A short 10-item version shows improved psychometric properties
599. Preliminary Study of S100B and Sema3A Expression Patterns in Regenerating Muscle Implicates P75-Expressing Terminal Schwann Cells and Muscle Satellite Cells in Neuromuscular Junction Restoration
600. Comparison of commercially available chamber slides for computer-aided analysis of human sperm
601. A Model-driven and Template-based Approach for Requirements Specification
602. Assessment of climate change impact on the Gomti River basin in India under different RCP scenarios
603. Assessing the impacts of land use-land cover changes on direct surface runoff: a remote sensing approach in Khulna City
604. Charging Scheduling of Electric Vehicle incorporating Grid-to-Vehicle (G2V) and Vehicle-to-Grid (V2G) technology in Smart-Grid
605. A hydro-geomorphologic assessment of flood generation potentiality in ungauged sub-basins and their prioritization based on traditional, statistical, MCDM and Nash-GIUH models of a tropical plateau-fringe River
606. A Simple Three-Level Switching Architecture to Enhance the Power Delivery Duration of Supercapacitor Banks in Electrified Transportation
607. Novel bank switching of supercapacitors with enhanced energy utilization for electric vehicular applications
608. Escala de AutorreflexÃ£o e Insight: Nova Medida de AutoconsciÃªncia Adaptada e Validada para Adultos Brasileiros = Self-Reflection and Insight Scale: New self-consciousness measure adapted and validated to Brazilian adults
609. Self-consciousness concept and assessment in self-report measures
610. Health disparity at the intersection of religion and caste: Evidence from India
611. Deflating the LLSVPs: Bundles of Mantle Thermochemical Plumes Rather Than Thick Stagnant â€œPilesâ€
612. Guillain-Barre Syndrome Following Zika Virus Infection Is Associated With a Diverse Spectrum of Peripheral Nerve Reactive Antibodies
613. Comparison of pain, functioning, coping, and psychological distress in patients with chronic low back pain evaluated for spinal cord stimulator implant or behavioral pain management
614. 3D face recognition using stereo images
615. Differences in Gene Expression of Endogenous Opioid Peptide Precursor, Cannabinoid 1 and 2 Receptors and Interleukin Beta in Peripheral Blood Mononuclear Cells of Patients With Refractory Failed Back Surgery Syndrome Treated With Spinal Cord Stimulation: Markers of Therapeutic Outcomes?
616. Magnetic resonance imaging in patients with spinal neurostimulation systems
617. Increased levels of interleukins 8 and 10 as findings of canine inflammatory mammary cancer
618. Malignant Skin Cancer Excision in Combined Therapy with Electro-Chemotherapy and Dermal Substitute
619. Adjuvant spinal cord stimulation improves wound healing of peripheral tissue loss due to steal syndrome of the hand: clinical challenge treating a difficult case
620. Spinal cord stimulation to achieve wound healing in a primary lower limb critical ischaemia referral centre
621. qTSL: A Multilayer Control Framework for Managing Capacity, Temperature, Stress, and Losses in Hybrid Balancing Systems
622. Level of sub-contracting design responsibilities in design and construct civil engineering bridge projects
623. The association between pain intensity and disability in patients with failed back surgery syndrome, treated with spinal cord stimulation
624. LC-MS/MS method for the quantitation of metabolites of eight commonly-used synthetic cannabinoids in human urine--an Australian perspective
625. Benchmarking circularity in supply chains: A systematic literature review
626. Further Validation of a Dutch Translation of the Sussex Oxford Compassion for the Self Scale in Samples of Crisis Line Volunteers, Military Personnel and Nursing Students
627. Treatment of allodynia by occipital nerve stimulation in chronic migraine rodent
628. 3D Bioprinting Human-Induced Pluripotent Stem Cells and Drug-Releasing Microspheres to Produce Responsive Neural Tissues
629. Machine learning approach for the development of a crucial tool in suicide prevention: The Suicide Crisis Inventory-2 (SCI-2) Short Form
630. Profiling and assessing the risks of image- and performance-enhancing drugs use during the COVID-19 lockdown
631. El pacto de silencio en los familiares de los pacientes oncolÃ³gicos terminales = The conspiracy of silence in families of the terminal oncological patients
632. Evaluating the Improvement of Blend Potency Measurements in the Feed Frame of a Rotary Tablet Press Using Combined NIR and Raman Spectroscopy
633. A simple hydrologic model for water resources simulation on rio grande basin, minas gerais state, Brazil
634. Gene action and combining ability for certain agronomic traits in red rice lines and commercial cultivars
635. Scalable solution for agricultural soil organic carbon measurements using laser-induced breakdown spectroscopy
636. Self-compassion and Symptoms of Stress, Anxiety, and Depression
637. Influence of durum wheat-faba bean intercrop on specific quality traits of organic durum wheat
638. Functional abilities and cognitive decline in adult and aging intellectual disabilities. Psychometric validation of an Italian version of the Alzheimer's Functional Assessment Tool (AFAST): analysis of its clinical significance with linear statistics and artificial neural networks
639. Cultural validation of the Self-Compassion Scale and the Subjective Happiness Scale and the influence of gender on self-compassion and subjective happiness in a Sri Lankan undergraduate population
640. RAINFALL-RUNOFF MODELING AND ITS PRIORITIZATION AT SUB-WATERSHED LEVEL USING SWAT MODEL: A CASE OF FINCAâ€™AA, OROMIA, WESTERN ETHIOPIA
641. Neurostimulation for the treatment of axial back pain: A review of mechanisms, techniques, outcomes, and future advances
642. Pain Relief Salvage with a Novel Minimally Invasive Posterior Sacroiliac Joint Fusion Device in Patients with Previously Implanted Pain Devices and Therapies
643. Relationship between compassion fatigue and satisfaction, self-compassion, social support and negative emotionality among registered nurses
644. Smart sustainable city manufacturing and logistics: A framework for city logistics node 4.0 operations
645. Spanish adaptation of the self-control scale and the brief self-control scale and evidences of validity in university population
646. Professional success and gender in family medicine: Design of scales and examination of gender differences in subjective and objective success among family physicians
647. Gas-centered mutation testing of Ethereum Smart Contracts
648. Testing the efficacy of â€˜Unlearningâ€™, a mindfulness and compassion-based programme for cultivating nonviolence in teenagers: A randomised controlled trial
649. A boost type switched-capacitor multi-level inverter for renewable energy sources with Self-Voltage balancing of capacitors
650. Neuromodulation of vegetative state through spinal cord stimulation: where are we now and where are we going?
651. Validity and reliability study of james supportive care screening for cancer patients
652. The relationship between school culture and student achievement
653. Br-I ordered CsPbBr2I perovskite single crystal toward extremely high mobility
654. Urban plant phenology monitoring: Expanding the functions of widespread surveillance cameras to nature rhythm understanding
655. Surveillance camera-based monitoring of plant flowering phenology
656. Psychometric properties of the inventory of callous-unemotional in preschool students
657. Meat quality and flavor compounds of soft-boiled chickens: effect of Chinese yellow-feathered chicken breed and slaughter age
658. Application of 3D- printed hydrogels in wound healing and regenerative medicine
659. Modelling rainfall runoff relations using HEC-HMS in a semi-arid region: Case study in Ain Sefra watershed, Ksour Mountains (SW Algeria)
660. Rainfall-runoff simulation in an experimental basin using GIS methods
661. Exploring the role of explicit and implicit self-esteem and self-compassion in anxious and depressive symptomatology following acquired brain injury
662. Estimation and comparision of curve numbers based on dynamic land use land cover change, observed rainfall-runoff data and land slope
663. Diamond-Based Supercapacitors with Ultrahigh Cyclic Stability Through Dual-Phase MnO2-Graphitic Transformation Induced by High-Dose Mn-Ion Implantation
664. Validating the Curve Number estimation approaches: A case study of an urbanizing watershed from Western Maharashtra, India
665. Evaluating somatic cell scores with a Bayesian Gaussian linear state-space model
666. Collagen I-matrigel scaffolds for enhanced schwann cell survival and control of three-dimensional cell morphology
667. Development of the Relationships Fostering Healthy Masculinities Scale
668. The Impact of Fatigue on Sleep and Other Non-Motor Symptoms in Parkinsonâ€™s Disease
669. Predicting the fire performance of LSF walls made of web stiffened channel sections
670. Using optmization coupled with simulation to construct layout solutions
671. Residential Appliance Identification Based on Spectral Information of Low Frequency Smart Meter Measurements
672. Feature extraction based on 2D compressive sensing for SAR automatic target recognition
673. Analytical and numerical simulation of seismic resilient rocking column with enhanced RC column and pre-pressed disc springs devices
674. Eddy-induced heat transport in the South China sea
675. Lateral evolution of the rift-to-drift transition in the South China Sea: Evidence from multi-channel seismic data and IODP Expeditions 367&368 drilling results
676. Redox-active â€œStructural Pillarâ€ molecular doping strategy towards High-Performance polyaniline-based flexible supercapacitors
677. Experimental study of the oriented differentiation of bone marrow derived mesenchymal stem cells into chondrogenic phenotype in a specific culture fluid
678. Towards user-centered and legally relevant smart-contract development: A systematic literature review
679. Energy management strategy of Supercapacitor/Fuel Cell energy storage devices for vehicle applications
680. Functional Outcomes of Isolated Infrasyndesmotic Fibula Fractures
681. Relations between sexually transmitted infection diagnosis and sexual compulsivity in a community-based sample of men who have sex with men
682. Sexual compulsivity among heterosexual college students
683. Neural representation and clinically relevant moderators of individualised self-criticism in healthy subjects
684. Valve timing optimisation of a spark ignition engine with skip cycle strategy
685. Measuring Emotional Intelligence Enhances the Psychological Evaluation of Chronic Pain
686. Ayahuasca may help to improve self-compassion and self-criticism capacities
687. A worthy self is a caring self: Examining the developmental relations between selfâ€esteem and selfâ€compassion in adolescents
688. Reconstruction of Subsurface Salinity Structure in the South China Sea Using Satellite Observations: A LightGBM-Based Deep Forest Method
689. Symptom clusters in patients with advanced cancer: a systematic review of observational studies
690. Coral reef geomorphology of the Spratly Islands: A simple method based on time-series of Landsat-8 multi-band inundation maps
691. Adding meaning to measurement: Initial Rasch analysis of the ASHA FACS Social Communication Subtest
692. Expanding the phenotypic spectrum of Succinyl-CoA ligase deficiency through functional validation of a new SUCLG1 variant
693. Ultraâ€“high-molecular-weight polyethylene sublaminar tape as semirigid fixation or pedicle screw augmentation to prevent failure in long-segment spine surgery: An ex vivo biomechanical study
694. Numerical validation of the shear compression specimen. Part I: Quasi-static large strain testing
695. Numerical validation of the shear compression specimen. Part II: Dynamic large strain testing
696. Modification of the Shear-Compression Specimen for Large Strain Testing
697. Quality of Nursing Care Perceived by People With HIV in Brazil: A Cross-Sectional Study
698. Software requirements testing approaches: a systematic literature review
699. Forensic Analysis of Synthetic Cathinones on Nanomaterials-Based Platforms: Chemometric-Assisted Voltametric and UPLC-MS/MS Investigation
700. Development and initial validation of the Adolescent and Adult Mindfulness Scale (AAMS)
701. Distribution chain diagrams for fresh fruit supply chains: A baseline for emission assessment
702. Fault Diagnosis and Separation of PMSM Rotor Faults Using Search Coil Based on MVSA and Random Forests
703. Dynamics of dissolved inorganic carbon in the South China Sea: A modeling study
704. A fast measurement method for hot large forgings and experiments
705. Microzone-explosion synthesis of porous carbon electrodes for advanced aqueous solid-state supercapacitors with a high-voltage gel electrolyte
706. Mesoscale oceanic eddies in the South China Sea from 1992 to 2012: evolution processes and statistical analysis
707. Theoretical prediction and analysis of hybrid material hat-shaped tubes with strengthened corner structures under quasi-static axial loading
708. A study of the mixed layer of the South China Sea based on the multiple linear regression
709. Full wave vector electromagnetic scattering from two-dimensional arbitrary random rough surfaces
710. Effectiveness of a mindfulness-based intervention on oncology nursesâ€™ burnout and compassion fatigue symptoms: A non-randomized study
711. Correlates of psychological inflexibility mediate the relation between alexithymic traits and positive emotions
712. Relationships between nurses' empathy, self-compassion and dimensions of professional quality of life: A cross-sectional study
713. Randomized Placebo-/Sham-Controlled Trials of Spinal Cord Stimulation: A Systematic Review and Methodological Appraisal
714. Electron backscatter diffraction characterisation of liquid route processed Ti6242S/SCS-6 composites
715. Mindfulness of voices, selfâ€compassion, and secure attachment in relation to the experience of hearing voices
716. Extraction of Phenolic Compounds from Cherry Seeds: A Preliminary Study
717. Multi-site exploration of sex differences in brain reactivity to smoking cues: Consensus across sites and methodologies
718. High-throughput analysis of 3D Schwann cell arrays for use in neural tissue engineering
719. Self-compassion and depressive symptoms in a Norwegian student sample
720. Risk in sustainable construction supply chains: construct development and measurement validation
721. Identification of the quasi-static and dynamic behaviour of projectile-core steel by using shear-compression specimens
722. Verifying the PCP-rule by five-center bond indices
723. A pilot study exploring the relationship between self-compassion, self-judgement, self-kindness, compassion, professional quality of life and wellbeing among UK community nurses
724. MITIGATEâˆ—: An innovative cyber-security maritime supply chain risk management system
725. Focusing on the 5F-MDMB-PICA, 4F-MDMB-BICA synthetic cannabinoids and their primary metabolites in analytical and pharmacological aspects
726. A Legal-Relationship Establishment in Smart Contracts: Ontological Semantics for Programming-Language Development
727. PRDM16 expression and function in mammalian cochlear development
728. Socially sustainable smart cities: mapping the research trends by co-word analysis
729. High-Frequency Spinal Cord Stimulation and Pregnancy: A Case Report
730. Evaluation of different topographic corrections for landsat TM data by prediction of foliage projective cover (FPC) in topographically complex landscapes
731. Acceptance and Action Questionnaire â€“ II: Confirmatory factor analysis and measurement invariance between Non-Hispanic White and Hispanic/Latinx undergraduates
732. Simultaneous Determination and Validation of 5F-ADBICA and 5F-NPB-22 in Whole Blood and Urine by LC/MSâ€“MS
733. Exposure to ambient fine particulate matter and health effects in the medicare population
734. Mindfulness moderates the relationship between emotional eating and body mass index in a sample of people with cystic fibrosis
735. Relationship between self-compassion and psychological well-being with the mediating role of resilience in people with multiple sclerosis
736. Self-compassion: A novel link with symptoms in schizophrenia?
737. Selfâ€compassion and emotion regulation difficulties in obsessiveâ€“compulsive disorder
738. Single-cell RNA-seq uncovers novel metabolic functions of Schwann cells beyond myelination
739. How do mindfulness-based interventions work? Strategies for studying mechanisms of change in clinical research
740. Identifying supply chain capabilities of construction firms in industrialized construction
741. Critical supply chain vulnerabilities affecting supply chain resilience of industrialized construction in Hong Kong
742. Critical capabilities of improving supply chain resilience in industrialized construction in Hong Kong
743. Differentiation strategies for defence supply chain design
744. The relationship between citizen readiness and the intention to continuously use smart city services: Mediating effects of satisfaction and discomfort
745. Impact of calendar life and cycling ageing on supercapacitor performance
746. Pseudo Bond Graph for fault detection and isolation of an industrial chemical reactor part I: Bond graph modeling
747. The contingent effect of supply chain strategies on the relationship between supply chain integration and operational performance in manufacturing context
748. Engineering orthogonality in supramolecular polymers: from simple scaffolds to complex materials
749. Fabrication of supramolecular semiconductor block copolymers by ring-opening metathesis polymerization
750. A GIS and SCS-CN based integrated model for rainfall-runoff simulation of ungauged watershed
751. An empirical method for peak discharge prediction in ungauged arid and semi-arid region catchments based on morphological parameters and SCS curve number
752. The validity and reliability study of the fear of intimacy scaleâ€™s Turkish form
753. Optimization of deep eutectic solvent based ultrasonic assisted microextraction for determination of zearalenone residues in foods
754. A psychometric study of the Suicide Cognitions Scale with psychiatric inpatients
755. Validation of somatic cell score-associated SNPs from Holstein cattle in Sudanese Butana and Butana x Holstein crossbred cattle
756. Corrigendum to â€œConnecting the resource nexus to basic urban service provision â€“ with a focus on water-energy interactions in New York Cityâ€ [31 (May) (2017) 83â€“94] (Sustainable Cities and Society (2017) 31 (83â€“94), (S2210670716305947), (10.1016/j.scs.2017.02.007))
757. Combining geospatial information and SCS-CN for surface runoff estimation in Rib watershed, upper Blue Nile Basin, Ethiopia
758. Supply chain strategies in response to a black-swan event: aÂ comparison of USA, French and Polish firms
759. Can we really teach the GenerationÂ Z? Opportunities andÂ challenges at secondary level
760. Kinetics of the Strain-Promoted Oxidation-Controlled Cycloalkyne-1,2-quinone Cycloaddition: Experimental and Theoretical Studies
761. Performance of Loss Models for Predicting Flood Hydrographs in a Semiarid Watershed with Limited Observations Using Deterministic and Probabilistic Hydrologic Models
762. Introducing an appropriate empirical method for estimating sediment delivery ratio (SDR) via sedimentometry of check-dams in small catchments in arid regions (Semnan province, Iran)
763. Impact of COVID-19 on the outreach strategy of cancer social service agencies in Singapore: A pre-post analysis with Facebook data
764. Effective treatment of low-risk acute GVHD with itacitinib monotherapy
765. Self-compassion mediates improvement in well-being in a mindfulness-based stress reduction program in a community-based sample
766. Dysautonomia rating scales in Parkinson's disease: sialorrhea, dysphagia, and constipation--critique and recommendations by movement disorders task force on rating scales for Parkinson's disease
767. Sensitivity analysis of runoff hydrographs due to temporal rainfall patterns in Makkah Al-Mukkramah region, Saudi Arabia
768. EVALUATION OF METHODS OF CALCULATION OF CONCENTRATION TIMES AND PEAK FLOW IN HYDROGRAPHIC BASINS: CASE OF RIO PACORA
769. Development of a Dispersive Liquid-Liquid Microextraction for Synthetic Cathinones in Biological Fluids Based on Principles of Green Analytical Toxicology
770. Validation of a fidelity scale for accelerated- experiential dynamic psychotherapy
771. Study and modelling of very flexible lines through simulation
772. Demonstrating mood repair with a situationâ€based measure of selfâ€compassion and selfâ€criticism
773. Awake vs. asleep placement of spinal cord stimulators: A cohort analysis of complications associated with placement
774. Factors associated with increased length of stay and risk of complications in 336 patients submitted to spine surgery. The role of a validated capture system (SAVES v2) as a first-line tool to properly face the problem
775. Introducing Ratiometric Fluorescence to MnO2 Nanosheet-Based Biosensing: A Simple, Label-Free Ratiometric Fluorescent Sensor Programmed by Cascade Logic Circuit for Ultrasensitive GSH Detection
776. The requirement for freshly isolated human colorectal cancer (CRC) cells in isolating CRC stem cells
777. Hydrodynamic design of deep ocean water discharge for the creation of a nutrient-rich plume in the South China Sea
778. Analysis of synthetic cannabinoids in wastewater of major cities in China
779. Reliability and Validity of Self-Concealment Scale in Chinese Older Adults
780. Self-concealment scale (SCS) in middle-aged people: psychometric features and cross-age equivalence test
781. Reliability and validity evaluation of the stigma of loneliness scale in Chinese college students
782. Surface chlorophyll-a concentration spatio-temporal variations in the Northern South China Sea detected using MODIS data
783. Sensitivity analysis of CN using SCS-CN approach, rain gauges and TRMM satellite data assessment into HEC-HMS hydrological model in the upper basin of Oum Er Rbia, Morocco
784. Towards a Characterization of Late Talkers: The Developmental Profile of Children with Late Language Emergence through a Web-Based Communicative-Language Assessment
785. Synthetic complex Weyl superconductors, chiral Josephson effect and synthetic half-vortices
786. NSSI questionnaires revisited: A data mining approach to shorten the NSSI questionnaires
787. Music performance anxiety and self-compassion in college-level music majors
788. Exploration of caregiver behavior on fear of emotion, spirituality, and self-compassion
789. Differentiation of adipose-derived stem cells into Schwann cell phenotype induces expression of P2X receptors that control cell death
790. A protein in the yeast saccharomyces cerevisiae presents dna binding homology to the p53 checkpoint protein and tumor suppressor
791. Estimation of the volume of distribution of some pharmacologically important compounds from their structural descriptors
792. Trade-off Pareto optimum design of an innovative curved damper truss moment frame considering structural and non-structural objectives
793. Recent Coyote Algorithm-Based Energy Management Strategy for Enhancing Fuel Economy of Hybrid FC/Battery/SC System
794. Robust hydrogen-consumption-minimization strategy based salp swarm algorithm for energy management of fuel cell/supercapacitor/batteries in highly fluctuated load condition
795. Linking stress coping styles with brain mRNA abundance of selected transcripts for Senegalese sole (Solea senegalensis) juveniles
796. Developing a rapid method for undertaking the World Ocean Assessment in data-poor regions - A case study using the South China Sea Large Marine Ecosystem
797. Revisiting the Method of Designing Salt Equalization Lines between the Saline Compartments of Drum Boilers of Thermal Power Plants
798. Operation of a burner rig for thermal gradient cycling of thermal barrier coatings
799. The triple-As supply chain competitive advantage
800. Occurrence and spatial distribution of legacy and novel brominated flame retardants in seawater and sediment of the South China sea
801. Numerical and analytical investigations on projectile perforation on steelâ€“concreteâ€“steel sandwich panels
802. Study on â€œprocessing traceâ€ of honey-fried Eriobotryae Folium formula granules based on electronic tongue technology
803. Private and public self-consciousness
804. Secretome analysis of human schwann cells derived from malignant peripheral nerve sheath tumor
805. Schwann Cell Stimulation of Pancreatic Cancer Cells: A Proteomic Analysis
806. Early childhood social competence scale (EC-SCS): Factor structure and psychometric properties
807. Reduction in the bullwhip effect through negotiation-based multi-agent systems
808. Single cell RNA sequencing of human FAPs reveals different functional stages in Duchenne muscular dystrophy
809. Cooperative assembly of the mitochondrial respiratory chain
810. Calcareous nannofossils in surface sediments of the eastern and western South China Sea
811. Gender differences in self-compassion: a latent profile analysis of compassionate and uncompassionate self-relating in a large adolescent sample
812. Self-compassion is associated with optimum self-care behaviour, medical outcomes and psychological well-being in a cross-sectional sample of adults with diabetes
813. Gender differences in self-compassion: A latent profile analysis of compassionate and uncompassionate self-relating in a large adolescent sample
814. Embracing the complexity of our inner worlds: Understanding the dynamics of self-compassion and self-criticism
815. Development of a measure for the assessment of peerâ€related positive emotional memories
816. The validation of the Body Image Acceptance and Action Questionnaire: Exploring the moderator effect of acceptance on disordered eating
817. Bidirectional Partial Power DC-DC Configuration for HESS interface in EV Powertrains
818. A study of new antimalarial artemisinins through molecular modeling and multivariate analysis
819. Statistical analysis and modelling of surface runoff from arable fields in central Europe
820. An evaluation matrix to compare computer hydrological models for flood predictions
821. Curtailment and stochastic curtailment to shorten the CES-D
822. On localized and coherent states on some new fuzzy spheres
823. Flood control simulation for disaster management in Wai Batu Merah River Basin
824. Buffering against maladaptive perfectionism in bipolar disorder: The role of self-compassion
825. The association between the suicide crisis syndrome and suicidal behaviors: The moderating role of personality traits
826. Sub-cellular spectrochemical imaging of isolated human corneal cells employing synchrotron radiation-based Fourier-transform infrared microspectroscopy
827. Effective rainfall estimates for St. Augustinegrass lawns under varying irrigation programs
828. Comparison of machine perfusion versus cold storage in kidney transplant recipients from expanded criteria donors: a cohort-based study
829. Spiritual Climate as is Perceived by Greek Clinical Nurses. A Validation study
830. Sexuality education, sexual communication, rape myth acceptance, and sexual assault experience among deaf and hard of hearing college students
831. A parallel longest common subsequence algorithm in UPC
832. Extraordinary long detection window of a synthetic cannabinoid metabolite in human urine - Potential impact on therapeutic decisions
833. Synthetic cannabinoids in hair - Pragmatic approach for method updates, compound prevalences and concentration ranges in authentic hair samples
834. Impact of I4.0 technologies and their interoperability on performance: future pathways for supply chain resilience post-COVID-19
835. The unique associations between self-compassion and eating disorder psychopathology and the mediating role of rumination
836. Angry rumination mediates the unique associations between self-compassion and anger and aggression
837. Measuring the influence of culture on mental processes and behaviour? A conceptual and empirical introduction of a multidimensional questionnaire on cultural variables
838. Wie lÃ¤sst sich der einfluss von kultur auf erleben und verhalten messen? Konzeptuelle und empirische einfÃ¼hrung einer multidimensionalen kultur-fragebogenbatterie = Measuring the influence of culture on mental processes and behaviour? A conceptual and empirical introduction of a multidimensional questionnaire on cultural variables
839. Effects of Mindfulness Based Cognitive Therapy (MBCT) and Compassion Focused Therapy (CFT) on symptom change, mindfulness, self-compassion, and rumination in clients with depression, anxiety, and stress
840. Biodegradable nano black phosphorus based SDF1-Î± delivery system ameliorates Erectile Dysfunction in a cavernous nerve injury rat model by recruiting endogenous stem/progenitor cells
841. Biodegradable nano black phosphorus based SDF1-alpha delivery system ameliorates Erectile Dysfunction in a cavernous nerve injury rat model by recruiting endogenous stem/progenitor cells
842. The Social Cognition Scale (SCS): Pilot study about the assessment of social cognition in people with schizophrenia
843. In-plane quantum-dot superlattices of InAs on GaAsSb/GaAs(001) for intermediate band solar-cells
844. Experimental validation of voltage unbalance suppression method of high voltage distribution system using static capacitors
845. A positive feedback process between tropical cyclone intensity and the moisture conveyor belt assessed with lagrangian diagnostics
846. A teleological approach to information systems design
847. Multiple worlds: A formalism for simulation based design
848. The relations between self-compassion, self-coldness, and psychological functioning among North American and Hong Kong college students
849. Psychometric properties of the Add Health School Connectedness scale for 18 sociocultural groups
850. Understanding the effects of a self-compassion intervention on emerging adults
851. Acoustic barriers made with sonic crystals using local resonators as scatterers
852. Revision of the psychological concept of self-compassion
853. Turkish validity and reliability of the Sexual Complaints Screener for Women
854. Turkish validity and reliability of the sexual complaints screener for men
855. Prognosis Following Surgery for Recurrent Ovarian Cancer and Diagnostic Criteria Predictive of Cytoreduction Success: A Systematic Review and Meta-Analysis
856. Performance evaluation of spatially distributed, CN-based rainfall-runoff model configurations for implementation in spatial land use optimization analyses
857. Purinergic responses of chondrogenic stem cells to dynamic loading
858. Screening for subclinical Cushing's syndrome in type 2 diabetes mellitus: low false-positive rates with nocturnal salivary cortisol
859. Assessment of hybrid Lean-Agile (Leagile) supply chain strategies
860. A brief introduction to compassion: Construct definition and measurement
861. Consideration of disruptive technologies and supply chain sustainability through Î±-discounting AHPâ€“VIKOR: calibration, validation, analysis, and methods
862. Contribution of Corrugated Steel Plate on the Blast-Resistance Performance of Steel-Concrete Composite Panels
863. Advances in design of copper-based nanostructures and nanocomposites for high-performance supercapacitors
864. Integrating Safety Certification into Model-Based Testing of Safety-Critical Systems
865. A Sequential Classification Strategy Applied to the Detection of Terrestrial Animal Lipid in Fish Oil by MID-Infrared Spectroscopy
866. Numerical and analytical studies of multi-cell steel-concrete-steel sandwich panels under blast load
867. Characterizing Deep Learning Package Supply Chains in PyPI: Domains, Clusters, and Disengagement
868. Characterization of the Human Oropharyngeal Microbiomes in SARS-CoV-2 Infection and Recovery Patients
869. Psychometric properties of the Sport Courage Scale for Chinese athletes
870. Digital supply chain model in Industry 4.0
871. Spanish validation of the short-form of the Avoidance and Fusion Questionnaire (AFQ-Y8) with children and adolescents
872. Validation of the Spanish version of the Child and Adolescent Mindfulness Measure (CAMM) with samples of Spanish and Chilean children and adolescents
873. Configuration and Control of a MVDC Hybrid Charging Station of Electric Vehicles with PV/Battery/Hydrogen System
874. Methodology supporting architecture validations (MAVS)
875. Exploring the Relationship Between Self-Compassion and Compassion for Others: The Role of Psychological Distress and Wellbeing
876. Traceability for sustainability â€“ literature review and conceptual framework
877. Supervisory Control System for a Grid-Connected MVDC Microgrid Based on Z-Source Converters With PV, Battery Storage, Green Hydrogen System and Charging Station of Electric Vehicles
878. Application of the rating scale model to examine the psychometric properties of the self-control scale
879. AplicaciÃ³n del modelo de escala de clasificaciÃ³n para examinar las propiedades psicomÃ©tricas de la Self-Control Scale = Application of the rating scale model to examine the psychometric properties of the Self-Control Scale
880. A randomized controlled trial of smartphone-based mindfulness training for smoking cessation: A study protocol
881. Un-Till we get it right: Applying finance industry standards to cram-down rates
882. Because excellencism is more than good enough: On the need to distinguish the pursuit of excellence from the pursuit of perfection
883. Hydrological analysis of the river Aude, France, flash flood on 12 and 13 November 1999
884. Short communication: investigation of the feasibility of genomic selection in Icelandic Cattle
885. Distribution of tetraether lipids in surface sediments of the northern South China Sea: Implications for TEX86 proxies
886. Lateral Talar Subluxation Measurements in Nonoperatively Managed Weber B Ankle Fractures
887. 3-D Electromagnetic Force Characteristics and Modeling of Double-Sided Air-Cored Superconducting Linear Synchronous Motor for EDS Train
888. Direct identification of volatile compounds in the artificially cultivated and wild Chinese medicinal materials (Semiliquidambar cathayesis) by headspace-gas chromatography-ion mobility spectrometry
889. 26th International Conference on Neural Information Processing, ICONIP 2019
890. Attachment style and self-identity as predictors of romantic relationship quality among emerging adults
891. Selfâ€compassion as a facet of neuroticism? A reply to the comments of Neff, TÃ³thâ€KirÃ¡ly, and Colosimo (2018)
892. Barriers to self-compassion in the eating disorders: The factor structure of the fear of self-compassion scale
893. An examination of the factor structure and preliminary assessment of the psychometric properties of a Hebrew translation of the Body Appreciation Scale-2 (BAS-2)
894. Reliability and Validity Study of a Turkish Version of the Sialorrhea Clinical Scale for Parkinson's Disease (SCS-TR)
895. One of a kind: User profiling by social curation
896. Accuracy of prediction of gene content in large animal populations and its use for candidate gene detection and genetic evaluation
897. Step counting for slow and intermittent ambulation based on a smartwatch accelerometer
898. A Smartwatch Step Counter for Slow and Intermittent Ambulation
899. Self-compassion as a buffer against concrete but not abstract threat
900. The relationship between self-compassion, concern for others, and parental burnout in childâ€™s chronic care management
901. Stem Cell-Associated Marker Expression in Canine Hair Follicles
902. Characterization of putative stem cell populations in the cornea using synchrotron infrared microspectroscopy
903. Novel experimental identification method for a supercapacitor multipore model in order to monitor the state of health
904. Soft Cell Simulator: A tool to study soft multi-cellular robots
905. Cultivating self-compassion in trauma survivors
906. First human experience with autologous Schwann cells to supplement sciatic nerve repair: report of 2 cases with long-term follow-up
907. The coming out experience, internalized homophobia and self-compassion in lgbq young adults
908. Testing GFM and GFL Inverters Operating With Synchronous Condensers
909. Evaluation of ModClark model for simulating rainfall-runoff in Tangrah watershed, Iran
910. A meta-analysis of heritability estimates for milk fatty acids and their genetic relationship with milk production traits in dairy cows using a random-effects model
911. A mutated salp swarm algorithm for optimum allocation of active and reactive power sources in radial distribution systems
912. The influence of deforestation on runoff generation and soil erosion (Case study: Kasilian Watershed)
913. Fair resource allocation and SBS selection for MC-NOMA-based HetNets supporting D2D communications
914. Self-compassion in Iranian Muslims: Relationships with integrative self-knowledge, mental health, and religious orientation
915. A fully automated tool for a comprehensive validation of analytical homogenization models
916. Energy Management Strategy with Adaptive Cut-off Frequency for Hybrid Energy Storage System in Electric Vehicles
917. Attentional features of mindfulness are better predictors of face recognition than empathy and compassion-based constructs
918. Exploring the relationship between mindfulness, compassion and unfamiliar face identification
919. Auction and Classification of Smart Contracts
920. Attempt to validate the Self-Construal Scale in French: Systematic approach and model limitation
921. Assessing suicidal cognitions in adolescents: Establishing the reliability and validity of the Suicide Cognitions Scale
922. Beyond discourse and competence: Science and subjugated knowledge in street children studies
923. Network model of nociceptive processing in the superficial spinal dorsal horn reveals mechanisms of hyperalgesia, allodynia, and spinal cord stimulation
924. Fears of compassion: Development of three self-report measures
925. Inter-comparison of Rain-Gauge, Radar, and Satellite (IMERG GPM) precipitation estimates performance for rainfall-runoff modeling in a mountainous catchment in Poland
926. An evaluation of integrated curriculum as it exists in mathematics and science SSS as well as the subsequent supportive presentation of those standards in eighth grade mathematics and science textbooks
927. Putting it into context: Creating a self-report measure of big picture appraisal
928. Collapse and burst test methods for sand screens
929. Secondary impacts of covid-19 pandemic in fatigue, self-compassion, physical and mental health of people with multiple sclerosis and caregivers: The teruel study
930. Characterization of regional cold-hydrothermal inflows enriched in arsenic and associated trace-elements in the southern part of the Duero Basin (Spain), by multivariate statistical analysis
931. Four cases of death involving the novel synthetic cannabinoid 5F-Cumyl-PEGACLONE
932. Questionnaires for assessment of female sexual dysfunction: a review and proposal for a standardized screener
933. The Self Cohesion Scale: A measure of the Kohutian concept of self cohesion
934. Stressors, academic performance, and learned resourcefulness in baccalaureate nursing students
935. Stressors and learned resourcefulness in baccalaureate nursing students: a longitudinal study
936. Derivation of Probabilistic Thresholds of Spatially Distributed Rainfall for Flood Forecasting
937. Understandings of the mindfulness construct: A clinican survey
938. Shape optimization of high-rise solar chimneys to improve the uniformity of flowrate distribution
939. Theoretical model for high-rise solar chimneys and optimum shape for uniform flowrate distribution
940. Two-Channel Information Fusion Weak Signal Detection Based on Correntropy Method
941. Genotype and Phylogenetic Diversity of Symbiodinium ITS2 Sequences Within Clade C in Three Typical Coral Species from Luhuitou Fringing Reef of the South China Sea
942. A prediction based on clustering and personality questionnaire data for IGD risk: A preliminary work
943. Psychometric properties of the Mindful Attention Awareness Scale (MAAS) in a first-episode psychosis sample
944. Bipolar affective disorder: An investigation of psychosocial risk factors among children and adolescents
945. Schwann cells and mesenchymal stem cells in laminin-or fbronectin-aligned matrices and regeneration across a critical size defect of 15 mm in the rat sciatic nerve
946. Adapting Rainfall Variability to Flood Risk: A Case Study of the Ghaggar River Basin
947. Measuring safety culture in a research and development centre: A comparison of two methods in the Air Traffic Management domain
948. Validity of the French Version of the Reynolds Empathy Scale Among Intensive Care Nurses
949. Evaluation of Nurse Empathy Perceived by the Standardized Patient in Simulation: A French Validation of the Jefferson Scale of Patient Perceptions of Physician Empathy
950. Evaluation of several rainfall products used for hydrological applications over West Africa using two high-resolution gauge networks
951. Self-esteem, self-compassion, defensive self-esteem, and related features of narcissism as predictors of aggression
952. Fast real-time corrective control strategy for overload relief in bulk power systems
953. Recent Advances on Boosting the Cell Voltage of Aqueous Supercapacitors
954. Behavioral operations management and supply chain coordination mechanisms: a systematic review and classification of the literature
955. Gradation of Clinical Holistic Response as New Composite Outcome to Evaluate Success in Spinal Cord Stimulation Studies for Pain
956. Exhaled-Breath Testing Using an Electronic Nose during Spinal Cord Stimulation in Patients with Failed Back Surgery Syndrome: An Experimental Pilot Study
957. Spinal Cord Stimulation-Naive Patients vs Patients With Failed Previous Experiences With Standard Spinal Cord Stimulation: Two Distinct Entities or One Population?
958. Construct validation of the social competence scale in preschool-age children
959. A software agent-component based framework for multi-agent supply chain modelling and simulation
960. Subtropical cyclones over the southwestern South Atlantic: Climatological aspects and case study
961. Absolute photon power measurements at the European XFEL instruments
962. The elusive nature of self-measurement: the self-construal scale versus the twenty statements test
963. Application of a mindfulness program among healthcare professionals in an intensive care unit: Effect on burnout, empathy and self-compassion
964. An exploration of the relationship between teachers' subjective well-being and professional learning communities
965. Diagnostic performance of cardiac magnetic resonance segmental myocardial strain for detecting microvascular obstruction and late gadolinium enhancement in patients presenting after a ST-elevation myocardial infarction
966. Use of Spinal Cord Stimulation in Elderly Patients with Multi-Factorial Chronic Lumbar and Non-Radicular Lower Extremity Pain
967. Development and initial validation of the Trauma-Related Shame Inventoryâ€”Short Form
968. The development and examination of a Self-Control Scale derived from a standard addiction research assessment
969. Surgical Back Risk Syndrome and Spinal Cord Stimulation: Better Safe Than Sorry
970. Soil and water assessment tool hydrologic and water quality evaluation of poultry litter application to small-scale subwatersheds in Texas
971. Compassionate hearts protect against wandering minds: Self-compassion moderates the effect of mind-wandering on depression
972. A preliminary program evaluation of a narrative therapy intervention for persons incarcerated for violent crime
973. The James Supportive Care Screening: integrating science and practice to meet the NCCN guidelines for distress management at a Comprehensive Cancer Center
974. Better family relationshipsâ€”Higher well-being: The connection between relationship quality and health related resources
975. A CRITICAL SUCCESS FACTORS MODEL FOR ADOPTING AND IMPLEMENTING SMART CONTRACTS
976. Effectiveness of spinal cord stimulation in chronic spinal pain: A systematic review
977. Phenotype-Based Screening of Synthetic Cannabinoids in a Dravet Syndrome Zebrafish Model
978. The Beagle 2 stereo camera system
979. Italian Version of the Relationship Profile Test (RPT-I): Temporal Stability, Construct Validity, and Cross-Cultural Comparison
980. A novel approach to 32-channel peripheral nervous system myelin imaging in vivo, with single axon resolution
981. Emergence and Progression of Behavioral Motor Deficits and Skeletal Muscle Atrophy across the Adult Lifespan of the Rat
982. Measuring outcome correlation for Bell cat state and geometric phase induced spin parity effect
983. Supercapacitor thermal characterization in transient state
984. Personal versus impersonal relationship compliance and their relationship with personality
985. The "neuro-mapping locator" software. A real-time intraoperative objective paraesthesia mapping tool to evaluate paraesthesia coverage of the painful zone in patients undergoing spinal cord stimulation lead implantation
986. Optimized power electronics interface for auxiliary power buffer based on supercapacitors
987. Validation of a methodology for neuro-urological and lumbosacral stimulation studies in domestic pigs: a humanlike animal model
988. Rule-based dynamic container stacking to optimize yard operations at port terminals
989. Analyzing long-term changes in water discharge and soil condition of ogouchi reservoir catchment
990. Observation of core phase ScS from the Mw 9.0 Tohoku-Oki earthquake with high-rate GPS
991. Application of three-dimensional interpolation in estimating diapycnal diffusivity in the south china sea
992. A genome-wide association study using international breeding-evaluation data identifies major loci affecting production traits and stature in the Brown Swiss cattle breed
993. Location reconstructions of attributed SCs by monopulse radar
994. Effect of Annealing on the Defects and Morphology of Cu9S5: Implication for Advanced Electrode for Asymmetric Supercapacitors
995. School culture: A validation study and exploration of its relationship with teachers' work environment
996. Hybrid construction of tissue-engineered nerve graft using skin derived precursors induced neurons and Schwann cells to enhance peripheral neuroregeneration
997. Quantifying flow speeds by using microstructure shear and temperature spectral analysis
998. Discovering personalized driver mutation profiles of single samples in cancer by network control strategy
999. Genetic discontinuities in a dominant mangrove Rhizophora apiculata (Rhizophoraceae) in the Indo-Malesian region
1000. Soil Moisture Accounting (SMA)based sediment graph models for small watersheds
1001. Short communication: detection of quantitative trait loci influencing somatic cell score in Spanish Churra sheep
1002. Estimating the total Ï€-electron energy
1003. Vertex-degree-based molecular structure descriptors of benzenoid systems and phenylenes
1004. Paddy rice response to phosphorus supply and sowing density in pre-germinated system
1005. Spaceâ€constrained scheduling optimization method for minimizing the effects of stacking of trades
1006. Contributions of Ih to feature selectivity in layer II stellate cells of the entorhinal cortex
1007. Lean Six Sigma, strategic control systems, and organizational performance for automotive suppliers
1008. Development of Machine Learning-Based Models to Predict Treatment Response to Spinal Cord Stimulation
1009. Validation of symptom clusters in patients with metastatic bone pain
1010. Observer for an omnidirectional mobile robot
1011. Childhood adversity, socioemotional functioning and generalized anxiety in young adults from mixed immigration status families
1012. Artificial neural network prediction of the psychometric activities of phenylalkylamines using DFT-calculated molecular descriptors
1013. Risk-Based Inspection and Rehabilitation Planning of Service Connections in Intermittent Water Supply Systems for Leakage Management in Arid Regions
1014. Modeling and Control of Fuel Cell/Supercapacitor Hybrid Source Based on Energetic Macroscopic Representation
1015. An examination of the relationship between self-compassion and burnout in human service providers working in underserved, faith-based contexts
1016. Hybrid energy storage sizing based on discrete Fourier transform and particle swarm optimization for microgrid applications
1017. Impact of asset management in a green supply chain
1018. Unveiling vulnerable smart contracts: Toward profiling vulnerable smart contracts using genetic algorithm and generating benchmark dataset
1019. Combinatorial tissue engineering partially restores function after spinal cord injury
1020. Psychometric analysis of the Slovak version of the compassionate engagement and action scales
1021. Robust psychometric analysis and factor structure of the Forms of Selfâ€“criticizing/Attacking and Selfâ€“Reassuring Scale
1022. Slovak validation of the Levels of Self-Criticism Scale: An Item Response Theory analysis
1023. Investigation of Reaction Mechanism and the Effects of Process Parameters on Ionic Liquidâ€“Based Delignification of Sugarcane Straw
1024. Individualized virtual reality for increasing self-compassion: Evaluation study
1025. Phylogenetic relationships of the commercial red snapper (Lutjanidae sp.) from three marine regions
1026. The role of self-compassion in physical and psychological well-being
1027. Control of satellites with minimum number of control actuators
1028. Internet-Administered Cognitive Behavioral Therapy for Hypersexual Disorder, With or Without Paraphilia(s) or Paraphilic Disorder(s) in Men: A Pilot Study
1029. Predicting treatment outcomes following an exacerbation of airways disease
1030. Exploring the Factors of Social Exclusion: Empirical Study of Rural Haryana, India
1031. Application of the HEC-HMS model for runoff simulation in a tropical catchment
1032. Secretory carcinoma of the minor salivary gland in the lip with ETV6-NTRK3 fusion: Case report and literature review
1033. Rapid sampling microdialysis as a novel tool for parenchyma assessment during static cold storage and hypothermic machine perfusion in a translational ex vivo porcine kidney model
1034. Rapid sampling microdialysis as a novel tool for parenchyma assessment during static cold storage and hypothermic machine perfusion in a translational ex vivo porcine kidney model
1035. Impulsivity and self-harm behavior in patients with synthetic cannabinoids dependence
1036. Die deutschsprachige Version der Sexual Sensation Seeking Scale und der Sexual Compulsivity Scale = The German versions of the Sexual Sensation Seeking Scale and the Sexual Compulsivity Scale
1037. Preliminary Validation of Sentinel 3a OLCI Bio-Optical Products in South China Sea
1038. Research and application on a coupled hydrological and hydrodynamic model in plain tidal river network
1039. Geomagnetic sudden commencement at the dawn-time dip equator
1040. Bias correction of sea surface temperature retrospective forecasts in the South China Sea
1041. ICA-domain filtering of Poisson noise images
1042. Single-cell sequencing: a promising approach for uncovering the mechanisms of tumor metastasis
1043. Application of DINCAE to reconstruct the gaps in chlorophyll-a satellite observations in the South China sea and West Philippine Sea
1044. Students' Image of the School Counselor in Junior High School
1045. Establishing the reliability and validity of a performance measure of big picture appraisal
1046. Response of Reinforced and Sandwich Concrete Panels Subjected to Projectile Impact
1047. Internal phosphorus load in a Mexican reservoir: forecast and validation
1048. Prosthetic socket fit comfort score
1049. The influence of treatment on the assessment of health-related quality of life (HRQoL) in patients with bronchial asthma - own research
1050. The Effect of Symptom Clusters and Sleep Disorder on Quality of Life among Patients with Chronic Obstructive Pulmonary Disease
1051. Application of sequential control based on Device Code strategy in 1000MW power plant
1052. Survival and complications of computer aided-designing and computer-aided manufacturing vs. conventionally fabricated implant-supported reconstructions: a systematic review
1053. Factor Structure of the Self-Construal Scale Revisited: Implications for the Multidimensionality of Self-Construal
1054. Revision and Replacement of Spinal Cord Stimulator Paddle Leads
1055. The impact of posttraumatic stress disorder on the psychological distress, positivity, and well-being of Australian police officers
1056. Cognitive and behavioral domains that reliably differentiate normal aging and dementia in down syndrome
1057. Sex work and the global pandemic
1058. Taking a mass-balance approach to assess marine plastics in the South China Sea
1059. The investigation of SNP in SOCS2 gene and its effect on milk yield, fat, protein, and somatic cell count in Awassi ewes
1060. Spatial modeling of river bank shifting and associated LULC changes of the Kaljani River in Himalayan foothills
1061. Evidence for Criterion-Related Validity of Pressure-Mediated Reflection Spectroscopy for Approximating Fruit and Vegetable Consumption among Preschool Children
1062. Systematic Review of Reflection Spectroscopy-Based Skin Carotenoid Assessment in Children
1063. Parenting-Related Self-Compassion Scale (P-SCS): Adaptation and validation in Indonesian parents
1064. Self-confidence in communication and identity in late adolescents
1065. Preliminary validation of a simultaneous Alcohol and Marijuana Consequence Scale for emerging adults
1066. Validity of the cohort of Crete in the Seven Countries Study: A time-series study applied to the cancer mortality trend between 1960 and 2011
1067. Interaction of experimental and modelling procedure to improve the fatigue behaviour of a metal matrix composite
1068. Interaction between cyclic loading and residual stresses in titanium matrix composites
1069. Blockchain and NFT-based traceability and certification for UAV parts in manufacturing
1070. Common bean cultivars performance under Rhizobium inoculation and the relation between yield components traits
1071. Psychometric evaluation of the Spanish and English versions of the spiritual coping strategies scale
1072. Construct validity of the Self-Compassion Scale-Short Form among psychotherapy clients
1073. Can clinical vignettes administered by mobile phone call be used to measure quality of contraceptive care practices in Malawi?
1074. Prediction of fishing intensity and trends across South China Sea biogeographic zones
1075. Double-image asymmetric cryptosystem using cylindrical diffraction and spectrum fusion and compression
1076. Mapping future offshore wind resources in the South China Sea under climate change by regional climate modeling
1077. The Chinese version of the Functionality Appreciation Scale: Psychometric properties and measurement invariance across gender and age
1078. Origin of a high-velocity layer: Insights from seismic reflection imaging (South China Sea)
1079. Differential gene expression in primary cultured sensory and motor nerve fibroblasts
1080. Differential Gene Expression in Primary Cultured Sensory and Motor Nerve Fibroblasts
1081. Forward Modeling of Scattering Centers from Coated Target on Rough Ground for Remote Sensing Target Recognition Applications
1082. LINC transmitter with double threshold SCS
1083. Torque Ripple Minimization of a Five-Phase Induction Motor under Open-Phase Faults Using Symmetrical Components
1084. Torque Ripple Suppression of a Five-phase Induction Motor under Single-phase Open
1085. Design of a fast charging system for service robots
1086. Thermohaline Dynamics in the Northern Continental Slope of the South China Sea: A Case Study in the Qiongdongnan Slope
1087. Remote sensing and validation of the South China Sea western boundary current in December 2003, 2004 and 2005
1088. Deep learning and image preprocessing-based crack repair trace and secondary crack classification detection method for concrete bridges
1089. Masculinity and barriers to seeking counseling: The buffering role of self-compassion
1090. A highly efficient liquid chromatography method for the determination of synthetic cannabinoid 5F-PB-22 in mice plasma at nano-concentration range: Application to pharmacokinetic study
1091. Suprachoroidal Delivery in Rats and Guinea Pigs Using a High-Precision Microneedle Injector
1092. Bond dissociation enthalpies of large aromatic carbon-centered radicals
1093. Psychometric validity of the Compassionate Engagement and Action Scale for Adolescents: A Swedish version
1094. Translation and validation of the Swedish version of the Self-Compassion Scale for Youth
1095. AI-based diagnosis and phenotype - Genotype correlations in syndromic craniosynostoses
1096. Selfâ€Balancing Supercapacitor Energy Storage System Based on a Modular Multilevel Converter
1097. Objective wearable measures and subjective questionnaires for predicting response to neurostimulation in people with chronic pain
1098. The Believability of Anxious Feelings and Thoughts Questionnaire (BAFT): A psychometric evaluation of cognitive fusion in a nonclinical and highly anxious community sample
1099. The effects of soil map changes on spillway design floods
1100. Enhancing supervisory relationships with values and committed action training
1101. The Dark Empath: Characterising dark traits in the presence of empathy
1102. The MAC-P program: A pilot study of a mindfulness and compassion program for youth with psychotic experiences
1103. Sports Courage in Malaysian Silat Athletes: Confirmatory Factor Analysis of the Malay Language Version
1104. [Electrodiagnosis in plexopathies; an update]
1105. Stereo Reconstruction Method for 3D Surface Wave Fields around a Floating Body Using a Marker Net in a Wave Tank
1106. Loss of Cx43 in murine sertoli cells leads to altered prepubertal sertoli cell maturation and impairment of the mitosis-meiosis switch
1107. Measurement of meaning in life
1108. Examining a brief self-compassion intervention for emotion regulation in individuals with exposure to trauma
1109. Automatic classification of large changes into maintenance categories
1110. A Visual and Narrative Timeline Review of Spinal Cord Stimulation Technology and US Food and Drug Administration Milestones
1111. Violence risk assessment in Hong Kong
1112. Characterizing Adult Cochlear Supporting Cell Transcriptional Diversity Using Single-Cell RNA-Seq: Validation in the Adult Mouse and Translational Implications for the Adult Human Cochlea
1113. B2B supply chain performance enhancement road map using data mining techniques
1114. Factor structure of the suicide cognitions scale in a national sample of female veterans
1115. Factor Structure of the Suicide Cognitions Scale in a National Sample of Female Veterans
1116. Effects of electrode positioning on perception threshold and paresthesia coverage in spinal cord stimulation
1117. Historic concepts vs. contemporary maritime regimes in UNCLOS: Chinaâ€™s claims in the South China Sea
1118. Deep-Water Bottom Current Research in the Northern South China Sea
1119. Transplantation of autologous Schwann cells for the repair of segmental peripheral nerve defects
1120. Crystal configuration and photoluminescent aspects of red-emitting combustion synthesized novel BaYZn3AlO7: Eu3+ nanophosphor
1121. Intra-testicular injection of adenoviral constructs results in Sertoli cell-specific gene expression and disruption of the seminiferous epithelium
1122. Evaluation of WMS model in basins without statistical data in southwestern Iran using Dicken's experimental method (case study: Kuhgel Basin of Khuzestan Province)
1123. Psychometric properties of the santa clara brief compassion scale-farsi version
1124. Impacts of digital technologies on supply chain performance: a system dynamics approach
1125. The performance prediction and improvement of SPH with the interaction-list-sharing method on PEZY-SCs
1126. A Compensation Strategy for Mitigating Intermittencies Within a PV Powered Microgrid Using a Hybrid Multilevel Energy Storage System
1127. Exploring the Optoelectronic and Photovoltaic Characteristics of Lead-Free Cs2TiBr6 Double Perovskite Solar Cells: A DFT and SCAPS-1D Investigations
1128. An extensive study on multiple ETL and HTL layers to design and simulation of high-performance lead-free CsSnCl3-based perovskite solar cells
1129. Severity Analysis of Secondary Crashes on High-Speed Roadways: Pattern Recognition Using Association Rule Mining
1130. Supply chain sustainability practices and governance for mitigating sustainability risk and improving market performance: A dynamic capability perspective
1131. Ripple effect modelling of supplier disruption: integrated Markov chain and dynamic Bayesian network approach
1132. Supplementary control based on current source coupling for improving dynamic characteristics of active distribution network
1133. Validation of a Chinese Version of the Five Facet Mindfulness Questionnaire in Hong Kong and development of a short form
1134. The effects of mindfulness-based stress reduction program on the mental health of family caregivers: A randomized controlled trial
1135. Engineering of Two-dimensional Cobalt-Glycine Complex Thin Sheets of Vertically Aligned Nanosheet Basic Building Blocks for High Performance Supercapacitor Electrode Materials
1136. The disposition to apologize
1137. Strain-induced bands of BÃ¼ngner formation promotes axon growth in 3D tissue-engineered constructs
1138. The validity of the three flows of compassion scales
1139. HFC-6000 triple module redundant (TMR) system and TÃœV SIL3 certification process
1140. Acoustic response of gas hydrate formation in sediments from South China Sea
1141. A steady-state simulation model of supplemental cooling system integrated with vapor compression refrigeration cycles for commercial airplane
1142. An Integrated Model of Pluvial Flood Risk and Adaptation Measure Evaluation in Shanghai City
1143. Fatty acid composition of surface sediments in the subtropical Pearl River estuary and adjacent shelf, Southern China
1144. Mapping bottom depth and albedo in coastal waters of the South China Sea islands and reefs using Landsat TM and ETM+ data
1145. Detecting Malposition of Coil Couple for Transcutaneous Energy Transmission
1146. Empirical ocean color algorithm for estimating particulate organic carbon in the South China Sea
1147. Self-centering companion spines with friction spring dampers: Validation test and direct displacement-based design
1148. A time-space varying distributed unit hydrograph (TS-DUH) for operational flash flood forecasting using publicly-available datasets
1149. Characterizing surface circulation in the Taiwan Strait during NE monsoon from Geostationary Ocean Color Imager
1150. Design of a Functionalized Carbon Cloth Substrate for a Ni and Co-Based High-Performance Supercapacitor
1151. DiseÃ±o y validaciÃ³n de una escala para medir el estilo controlador del estudiante de EducaciÃ³n Superior
1152. Accepting our weaknesses and enjoying better relationships: An initial examination of self-security
1153. The Differentiation Stage of Transplanted Stem Cells Modulates Nerve Regeneration
1154. Effect of growing watershed imperviousness on hydrograph parameters and peak discharge
1155. Hierarchical Nanosheets/Walls Structured Carbon-Coated Porous Vanadium Nitride Anodes Enable Wide-Voltage-Window Aqueous Asymmetric Supercapacitors with High Energy Density
1156. Validation of the Chinese version of Selfâ€‘Compassion Scale for Youth (SCSâ€‘Y)
1157. The Self-Construal Scale: An examination of its reliability and validity among Chinese university students
1158. Part family grouping method for reconfigurable manufacturing system considering process time and capacity demand
1159. Developing Customized NRCS Unit Hydrographs for Ungauged Watersheds in Indiana
1160. Assessment of waterlogging hazard in Tianhe District of Guangzhou City using remote sensing and GIS
1161. Weighted Gene Co-Expression Network Analysis Identified Cancer Cell Proliferation as a Common Phenomenon During Perineural Invasion
1162. Performance Improvement of Model Predictive Current Control of Fault-Tolerant Five-Phase Flux-Switching Permanent Magnet Motor Drive
1163. Model predictive current control of open-circuit fault-tolerant five-phase flux-switching permanent magnet motor drives
1164. Artificial intelligence-powered early identification of refractory constipation in children
1165. Foam Evolution Inspired Modeling for Staged Construction of Ultra-Dense Small Cell Networks
1166. Simultaneous Determination of 112 Synthetic Cannabinoids in Hair by Ultra-High Performance Liquid Chromatography-Tandem Mass Spectrometry
1167. Degeneration of Key Structural Components Resulting in Ageing of Supercapacitors and the Related Chemical Ageing Mechanism
1168. Nonlinear finite element modelling and parametric study of curved steel-concrete-steel double skin composite panels infilled with ultra-lightweight cement composite
1169. Experimental and analytical studies of curved steel-concrete-steel sandwich panels under patch loads
1170. Structural behaviour of steel-concrete-steel sandwich composite wall subjected to compression and end moment
1171. Residual Flexural Performance of Double-Layer Steelâ€“RLHDC Composite Panels after Impact
1172. Construct validity and measurement invariance of the Asian Family Characteristics Scale in the Thai population
1173. ICVTS on-line discussion A Cost-benefit success rates are mandatory for SCS
1174. 3D CFD wind flow analysis technique applied to a parabolic solar tracker for two extreme weather conditions with experimental results and a controller proposition
1175. The extensive solid-form landscape of sulfathiazole: Geometrical similarity and interaction energies
1176. The sexual consent scale-revised: development, reliability, and preliminary validity
1177. Toward a full implementation of SCIM functional block in IMS framework
1178. Joint wireless charging and hybrid power based resource allocation for LTE-A wireless network
1179. Spinal Cord Stimulation for the Treatment of Failed Neck Surgery Syndrome: Outcome of a Prospective Case Series
1180. BURST(able): A Retrospective, Multicenter Study Examining the Impact of Spinal Cord Stimulation with Burst on Pain and Opioid Consumption in the Setting of Salvage Treatment and "Upgrade"
1181. Selective Radiofrequency Stimulation of the Dorsal Root Ganglion (DRG) as a Method for Predicting Targets for Neuromodulation in Patients With Post Amputation Pain: A Case Series
1182. Application of physically based semi-distributed hec-hms model for flow simulation in tributary catchments of kaohsiung area taiwan
1183. How do dynamic capabilities enable hotels to be agile and resilient? A mediation and moderation analysis
1184. Finding diamonds in the rough: evaluating the quality of meta-analyses in chronic pain â€“ an infographic
1185. Further evaluation of the criterion validity of the Severe Impairment Battery for the assessment of cognitive functioning in adults with Down syndrome
1186. Core patient-reported outcome measures for chronic pain patients treated with spinal cord stimulation or dorsal root ganglia stimulation
1187. Reconstruction and analysis of long-term satellite-derived sea surface temperature for the South China Sea
1188. Development of smart application for house condition survey
1189. Balancing control and trust to manage CSR compliance in supply chains
1190. Psychometric properties of the sexual compulsivity scale in men who have sex with men in spanish population
1191. Psychometric Properties of the Sexual Compulsivity Scale in Men Who Have Sex with Men in Spanish Population
1192. A Curated Solidity Smart Contracts Repository of Metrics and Vulnerability
1193. Rainfall-runoff modelling of Doddahalla watershedâ€”an application of HEC-HMS and SCN-CN in ungauged agricultural watershed
1194. The impact of an urbanizing tropical watershed to the surface runoff
1195. TOWARDS SMART CONSTRUCTION SITES FOR EFFECTIVE PROJECT DELIVERY
1196. Genome-wide association studies for milk somatic cell score in romanian dairy cattle
1197. Role of health simulation centres in the COVID-19 pandemic response in Italy: a national study
1198. Smart computing models of California bearing ratio, unconfined compressive strength, and resistance value of activated ash-modified soft clay soil with adaptive neuro-fuzzy inference system and ensemble random forest regression techniques
1199. Model-Driven approach to integrate requirements for safety-critical systems
1200. Can We Boost Treatment Adherence to an Online Transdiagnostic Intervention by Adding Self-Enhancement Strategies? Results From a Randomized Controlled Non-inferiority Trial
1201. Modeling effects of changing land use/cover on daily streamflow: An Artificial Neural Network and curve number based hybrid approach
1202. Self-construal and behavioral motivation systems among patients with depression in Indonesia: A hospital-based study
1203. Decellularized Matrix Induced Spontaneous Odontogenic and Osteogenic Differentiation in Periodontal Cells
1204. Digital Supply Chain Twins: Managing the Ripple Effect, Resilience, and Disruption Risks by Data-Driven Optimization, Simulation, and Visibility
1205. Determination of the polyphenol contents in Macedonian grapes and wines by standardized spectrophotometric methods
1206. Smart control system of human resources potential of the region
1207. Mindfulness Based Flourishing Program: A cross-cultural study of Hong Kong Chinese and British participants
1208. GIS-based watershed management modeling for surface runoff calculation in Tatara River basin, Japan
1209. Sustainably resilient supply chains evaluation in public transport: A fuzzy chance-constrained two-stage DEA approach
1210. Spinal Cord High-Frequency Stimulation. The Current Experience and Future Directions
1211. Improved Control Strategy of a Supercapacitor-Based Energy Recovery System for Elevator Applications
1212. Highly Reliable Monitoring and Equalization in a Hybrid Energy Storage System with Batteries and Supercapacitors for Electric Motor Drives in Building Applications
1213. An Enhanced Equalization and Energy Support Algorithm for Lithium-Ion Battery Storage Systems in Electric Motor Drives
1214. T-box transcription factor 21 is expressed in terminal Schwann cells at the neuromuscular junction
1215. Quality of life and comorbidity score as prognostic determinants in non-small-cell lung cancer patients
1216. Measuring microbial food safety output and comparing self-checking systems of food business operators in Belgium
1217. The mediating effect of perceived burdensomeness on the relation between depressive symptoms and suicide ideation in a community sample of older adults
1218. Concurrent validity of skin carotenoid status as a concentration biomarker of vegetable and fruit intake compared to multiple 24-h recalls and plasma carotenoid concentrations across one year: a cohort study
1219. Multi-criteria decision making to validate performance of RBC-based formulae to screen Î² -thalassemia trait in heterogeneous haemoglobinopathies
1220. Multi-criteria decision making to validate performance of RBC-based formulae to screen [Formula: see text]-thalassemia trait in heterogeneous haemoglobinopathies
1221. On the Ia - S relation of the SCS-CN method
1222. Water balance modeling of Tandula (India) reservoir catchment using SWAT
1223. Five-Level Common-Ground-Type Boosting Inverter With Lesser Capacitor-Stored Energy
1224. A Nine-Level Common-Ground Type Boost Inverter for PV Applications
1225. A Five-Level X-Type Boosting Inverter With Reduced Stored Energy of Switched-Capacitors
1226. Does self-construal impact awareness of emotion in others?
1227. Self-criticism as a mediator in the relationship between unhealthy perfectionism and distress
1228. Identification of potential sites for rainwater storage to enhance agricultural practices in kirthar mountain range
1229. Optimal Sizing of BESS for Attaining Frequency Stability under High PV Penetration
1230. Cyber risk management strategies and integration: toward supply chain cyber resilience and robustness
1231. Fuzzy logic-based energy management strategy on dual-source hybridization for a pure electric vehicle
1232. Improving the Two-Color Temperature Sensing Using Machine Learning Approach: GdVO4:Sm3+ Prepared by Solution Combustion Synthesis (SCS)
1233. ExoNav: A Novel Robotic Steering and Latching Mechanism for Spinal Cord Stimulation Electrodes
1234. Hydrologic modeling of watersheds using remote sensing, gis and agnps
1235. Regional calibration of SCS-CN L-THIA model: Application for ungauged basins
1236. Forecasting the impact of epidemic outbreaks on the supply chain: modelling asymptomatic cases of the COVID-19 pandemic
1237. Estimation of the Genetic Parameters and G Ã— e Interactions for Growth Traits and Shell-Closing Strength in Pacific Oysters (Crassostrea gigas)
1238. Reliable Event Detection via Multiple Edge Computing on Streaming Traffic Social Data
1239. Joint multi-site domain adaptation and multi-modality feature selection for the diagnosis of psychiatric disorders
1240. Comparative proteomic analysis of primary schwann cells and a spontaneously immortalized schwann cell line RSC 96: a comprehensive overview with a focus on cell adhesion and migration related proteins
1241. Thermo-viscoplastic behavior of DP800 steel at quasi-static, intermediate, high and ultra-high strain rates
1242. Glass-activated regeneration of volumetric muscle loss
1243. Impact of the quasi-biweekly oscillation over the western North Pacific on East Asian subtropical monsoon during early summer
1244. Numerical modeling and experimental validation for suspended ceiling system with free boundary condition
1245. Diatoms from the surface sediments of the South China Sea and their relationships to modern hydrography
1246. Tsunami hazard assessment at guanghai bay of the south china sea
1247. Involvement of Abnormal p-Î±-syn Accumulation and TLR2-Mediated Inflammation of Schwann Cells in Enteric Autonomic Nerve Dysfunction of Parkinsonâ€™s Disease: an Animal Model Study
1248. Involvement of Abnormal p-alpha-syn Accumulation and TLR2-Mediated Inflammation of Schwann Cells in Enteric Autonomic Nerve Dysfunction of Parkinson's Disease: an Animal Model Study
1249. Two-dimensional Co3O4 thin sheets assembled by 3D interconnected nanoflake array framework structures with enhanced supercapacitor performance derived from coordination complexes
1250. Software-Defined Virtual Synchronous Condenser
1251. The effect of spinal cord stimulation on epileptic seizures suppression
1252. QSPR study on the gas/particle partition coefficient of polychlorinated biphenyls using the molecular distance-edge vector index
1253. Conceptualizing antecedent runoff condition using recurrence relation to modify SCS model
1254. Study of the oversized capacity and the increased energy loss of hybrid energy storage systems and design of an improved controller based on the low-pass filter
1255. Symptom clusters in advanced cancer
1256. A CMOS technology for educational activities and academic projects
1257. Genetic Parameters of Somatic Cell Score in Florida Goats Using Single and Multiple Traits Models
1258. Selecting suitable behavioural tests to identify proactive and reactive stress coping styles in flathead grey mullet (Mugil cephalus) juveniles
1259. IL-8 regulates the stemness properties of cancer stem cells in the small-cell lung cancer cell line H446
1260. Recent deep water ventilation in the South China Sea and its paleoceanographic implications
1261. A study on influence of predictor multicollinearity on performance of the stepwise regression prediction equation
1262. A nonlinear artificial intelligence ensemble prediction model for typhoon intensity
1263. Identifying Oceanic Responses with Validated Satellite Observations after the Passage of Typhoons in the Northern South China Sea
1264. Blind background extraction from videos in the cloud
1265. Testicular Lmcd1 regulates phagocytosis by Sertoli cells through modulation of NFAT1/Txlna signaling pathway
1266. Comparative age and growth of Uroteuthis chinensis and Uroteuthis edulis from China Seas based on statolith
1267. Species identification of two Loliginidae cryptic species in China Seas with morphological and molecular methods
1268. Exploring the nexuses of aging, mental health, and obesity: A community based study in northern india
1269. Onset of the 1998 summer monsoon in the South China Sea - Evidence from satellite microwave scatterometer
1270. Community-weighted mean traits play crucial roles in driving ecosystem functioning along long-term grassland restoration gradient on the Loess Plateau of China
1271. Cloud-edge collaboration composition and scheduling for flexible manufacturing service with a multi-population co-evolutionary algorithm
1272. Combination of LiCs and EDLCs with batteries: A new paradigm of hybrid energy storage for application in EVs
1273. Electrical circuit modelling of double layer capacitors for power electronics and energy storage applications: A review
1274. Constructing a dialogue at science museum: A practice of 'Do you know X?' From the perspective of interaction analysis
1275. Establishing Minimal Clinically Important Difference in Sleep Outcomes after Spinal Cord Stimulation in Patients with Chronic Pain Disorders
1276. Performance of land surface schemes on simulation of land falling tropical cyclones over Bay of Bengal using ARW model
1277. Simulation of track and intensity of TCs over Bay of Bengal: Sensitivity to micro physics and Cu parameterization schemes
1278. CD4 Inhibits Helper T Cell Activation at Lower Affinity Threshold for Full-Length T Cell Receptors Than Single Chain Signaling Constructs
1279. CD4 Inhibits Helper T Cell Activation at Lower Affinity Threshold for Full-Length T Cell Receptors Than Single Chain Signaling Constructs
1280. The development and validation of the Short Cyberchondria Scale (SCS)
1281. Considerations on the psychometric properties and validity of the Spinal Cord Injury Secondary Conditons Scale
1282. Automated discovery of mimicry attacks
1283. Rainfall-runoff simulation in Cache River Basin, Illinois, using HEC-HMS
1284. The reliability and validity of the Chinese version of the Mindful Self-Care Scale for College Students
1285. Enabling collaborative dynamic capabilities in strategic communities: Firm- vs network-centric perspectives
1286. A two-model integrated technology adoption framework for using blockchain in supply chain management: attitude towards blockchain as a mediator
1287. Advances in Spinal Cord Stimulation for Treatment of Chronic Pain
1288. Estimating water availability using the SCS-CN method based on long term hydrologic simulation and the geographic information system
1289. Soil carbon stocks of semi-arid grasslands in northern Mexico
1290. Subnormothermic perfusion with h2s donor ap39 improves dcd porcine renal graft outcomes in an ex vivo model of kidney preservation and reperfusion
1291. A state-of-the-art review on performance measurement petri net models for safety critical systems of NPP
1292. Green synthesised Mn3O4and poly (o-phenylenediamine) for antimicrobial textile-based supercapacitor applications
1293. Validity and reliability of Turkish Version of the Craving Experience Questionnaire in the assessment of cigarette smoking
1294. Treatment of fish farm sludge supernatant by aerated filter beds and steel slag filters-effect of organic loading rate
1295. Carbon Capture, Utilization, and Storage Risks from Supply Chain Perspective: A Review of the Literature and Conceptual Framework Development
1296. Identification of Possible Incoming Runoff using Different Combinations of Extreme Rainfall Events in a Semi-arid Context: Banas River, Bisalpur Dam Catchment
1297. Identifying Potential Rainwater Harvesting Sites of a Semi-arid, Basaltic Region of Western India, Using SCS-CN Method
1298. In vitro metabolic profiles of adamantyl positional isomers of synthetic cannabinoids
1299. Age estimation in the harbour seal (Phoca vitulina) based on the closure of skull sutures and synchondroses
1300. A generalized switched-capacitor-based modular T-type inverter topology with reduced switch count
1301. A Common-Ground-Type Five-Level Inverter with Dynamic Voltage Boost
1302. Populism and party: Society developmental regimes in Tamil Nadu and West Bengal
1303. Effects of spinal cord stimulation on heart rate variability in patients with chronic pain
1304. Preliminary reliability and validity of a Spinal Cord Injury Secondary Conditions Scale
1305. Super Capacitor and TCSC Based Territorial Control Strategy for Frequency Regulation of Multi-Area Interconnected Power System
1306. A spherical fuzzy assessment framework for evaluating the challenges to LARG supply chain adoption in pharmaceutical companies
1307. Associations between low self-control and aggression among Malaysian male prisoners
1308. A bioelectronic route to compassion: Rationale and study protocol for combining transcutaneous vagus nerve stimulation (tVNS) with compassionate mental imagery
1309. Energy management of a dc microgrid composed of photovoltaic/fuel cell/battery/supercapacitor systems
1310. Feature models as service contracts in service oriented architecture
1311. Multiple software product lines for service oriented architecture
1312. The influence of organisational safety climate on group safety outcomes: The mediation role of supervisor safety communication and monitoring
1313. Use of Nonofficial Intermittent Waterfall Occurrence Data for the Validation of an Infiltration Model for Volcanic Jeju Island, Korea
1314. Aggregative Game for Distributed Charging Strategy of PEVs in a Smart Charging Station
1315. Distributed plug-in electric vehicles charging strategy considering driver behaviours and load constraints
1316. The impact of eurythmy therapy on stress coping strategies and health-related quality of life in healthy, moderately stressed adults
1317. DNA-Wrapped CNT Sensor for Small Nucleic Acid Detection: Influence of Short Complementary Sequence
1318. Single-Cell RNA Transcriptome Helps Define the Limbal/Corneal Epithelial Stem/Early Transit Amplifying Cells and How Autophagy Affects This Population
1319. The roles of self-compassion and self-coldness in the relationships between inferiority and stress and anxiety among gifted adolescents
1320. Turkish version of body awareness questionnaire: Validity and reliability study
1321. ADVANCED THERMAL PROFILING OF TURBOCHARGER COMPRESSOR WHEELS USING PHOSPHORESCENCE THERMAL HISTORY COATINGS
1322. Investigating differences in self-compassion levels: Effects of gender and age in a Greek adult sample
1323. Investigating the Role of Second Chance Schools and COVID-19 Pandemic on the Mental Health and Self-Image of Greek Adult Students
1324. Impact of the Abbreviated Suicide Crisis Syndrome Checklist on Clinical Decision Making in the Emergency Department
1325. Rainfall-surface runoff estimation for the Lower Bhavani basin in south India using SCS-CN model and geospatial techniques
1326. Analysis of critical barriers in the sustainable supply chain ofÂ MSMEs: a case of Makhana (Foxnut) industry
1327. Using interpretative phenomenological analysis to assess identity formation among users of synthetic cannabinoids
1328. Auto-calibration of HEC-HMS Model for Historic Flood Event under Rating Curve Uncertainty. Case Study: Allala Watershed, Algeria
1329. An integrated approach of flash flood analysis in ungauged Mediterranean watersheds using post-flood surveys and unmanned aerial vehicles
1330. Evaluation of hydrological and hydraulic models applied in typical mediterranean ungauged watersheds using post-flash-flood measurements
1331. Event-based and continous floodÂ modeling in Zijinguan watershed, Northern China
1332. Research design considerations for randomized controlled trials of spinal cord stimulation for pain: Initiative on Methods, Measurement, and Pain Assessment in Clinical Trials/Institute of Neuromodulation/International Neuromodulation Society recommendations
1333. Validity and reliability of an adapted social capital scale among Indian adults
1334. Importance and awareness of corporate social reporting practices in banks - An empirical analysis in Punjab
1335. Exploring associations between self-compassion, self-criticism, mental health, and quality of life in adults with cystic fibrosis: Informing future interventions
1336. A daily diary study of self-compassion, body image, and eating behavior in female college students
1337. The impact of spinal cord stimulation on physical function and sleep quality in individuals with failed back surgery syndrome: a systematic review
1338. Evidence for a stem-cell lineage in corneal squamous cell carcinoma using synchrotron-based Fourier-transform infrared microspectroscopy and multivariate analysis
1339. Psychometric properties of the Lesbian, Gay, and Bisexual Identity Scaleâ€“Turkish (LGBIS-TR)
1340. Use of a brief version of the self-compassion inventory with an international sample of people with HIV/AIDS
1341. Effects of Neurod1 Expression on Mouse and Human Schwannoma Cells
1342. A long-term response-based rainfall-runoff hydrologic model: Case study of the upper Blue Nile
1343. Baclofen-induced Changes in the Resting Brain Modulate Smoking Cue Reactivity: A Double-blind Placebo-controlled Functional Magnetic Resonance Imaging Study in Cigarette Smokers
1344. Dynamic simulations in support of installation of light rail tracks on the Homer H. Hadley Memorial floating bridge
1345. QUANTUM ALGORITHMS FOR THE SHORTEST COMMON SUPERSTRING AND TEXT ASSEMBLING PROBLEMS
1346. Quantum Algorithm for the Shortest Superstring Problem
1347. Bio-Heat Model of Kilohertz-Frequency Deep Brain Stimulation Increases Brain Tissue Temperature
1348. Multi-criteria optimization of sports compression socks using Taguchi-VIKOR statistical approach
1349. Scalable culture techniques to generate large numbers of purified human Schwann cells for clinical trials in human spinal cord and peripheral nerve injuries
1350. Safety of Spinal Cord Stimulation in Patients Who Routinely Use Anticoagulants
1351. Rooftop Solar PV Assisted E-Rickshaw with Hybrid Energy Storage System
1352. Genetic polymorphisms of TRAPPC9 and CD4 genes and their association with milk production and mastitis resistance phenotypic traits in Chinese Holstein
1353. Demystifying the impact of university graduateâ€™s core competencies on work performance: A Saudi industrial perspective
1354. Role of Heterocyclic Organic Compounds on the Optoelectronic Properties of Halide Perovskite Single Crystals
1355. Psychometric Properties of the Persian Version of the Early Life Experiences Scale (ELES)
1356. Estimating daughter yield deviation and validation of genetic trend for somatic cell score in holstein cattle using random regression test day model
1357. Development of Schwann cell-seeded conduit using chitosan-based biopolymers for nerve repair
1358. A study on the nanoindentation behaviour of single crystal silicon using hybrid MD-FE method
1359. The behavior of steel-concrete-steel sandwich composite beams with box-profile shear connectors: Experimental and numerical
1360. Brain activity monitoring system with HDMI interface developed on an FPGA
1361. A model validation study of hierarchical and distributed web caching model
1362. Correlation Between Aspects of Perceived Patient Loneliness and Spinal Cord Stimulation Outcomes
1363. A Daily Water Balance Model Based on the Distribution Function Unifying Probability Distributed Model and the SCS Curve Number Method
1364. SCS Curve Number to Model Flooding in the Upper St. Johns River Using Retrieved Remotely Sensed Precipitation from NEXRAD, and TRMM
1365. Evaluation of appropriate locations and capacities of on-farm ponds in Northeast Thailand
1366. Social capitals and english language learning in an iranian language institute
1367. Exploring the relationship between social capitals and english language achievement within a specific grade and context
1368. Physical layer security in shotgun cellular systems over correlated/independent shadow fading channels
1369. Reviewing of Indonesian studentsâ€™ scientific communication skills: A structural equation modeling analysis
1370. The effectiveness of acceptance and commitment therapy for social anxiety disorder: A randomized clinical trial
1371. Comparing the Soil Conservation Service model with new machine learning algorithms for predicting cumulative infiltration in semi-arid regions
1372. Mindfulness-based stress reduction for healthy individuals: A meta-analysis
1373. Interpersonal mindfulness questionnaire: Scale development and validation
1374. Mindfulness and self-compassion as predictors of humor styles in US and Russia
1375. NetREm: Network Regression Embeddings reveal cell-type transcription factor coordination for gene regulation
1376. Investigating current paradigms in supply chain risk management â€“ a bibliometric study
1377. A social epidemiological perspective on local tennis league participation: A multigroup moderated-mediation structural analysis using PLS-SEM
1378. The Relationship between Runs of Homozygosity and Inbreeding in Jersey Cattle under Selection
1379. Interpersonal Mindfulness in Parenting Scale: Testing the psychometric properties of a Korean version
1380. DEVS-based doctrine validation of fleet anti-air defense
1381. Pharmacokinetic and Safety Comparison of Fixed-Dose Combination of Cilostazol/Rosuvastatin (200 + 20 mg) Versus Concurrent Administration of the Separate Components in Healthy Adults
1382. Stressed, depressed, and rank obsessed: Individual differences in compassion and neuroticism predispose towards rank-based depressive symptomatology
1383. Stressed, depressed, and rank obsessed: Individual differences in compassion and neuroticism predispose towards rankâ€based depressive symptomatology
1384. Compassion mediates poor sleep quality and mental health outcomes
1385. The Spiritual Contagion Scale: A measure of beliefs in the transfer of metaphysical properties
1386. Assessing compassion in Korean population: Psychometric properties of the Korean version of Sussex-Oxford Compassion Scales
1387. Exploration of sub-annual calibration schemes of hydrological models
1388. Improving sound transmission through triple-panel structure using porous material and sonic crystal
1389. Agent-based simulation of drug disposition in cirrhotic liver
1390. Experiments on the thermal stratification in the branch of NPP
1391. Design method for broadband free-space electromagnetic cloak based on isotropic material for size reduction and enhanced invisibility
1392. Comprehensive SC renewal planning scheme considering optimal allocation and reallocation
1393. A technique for SC renewal planning considering optimal allocation and life cycle cost
1394. Establishing Canadian metrics for self-report measures used to assess hypersexuality
1395. Understanding the Latent Structure of Hypersexuality: A Taxometric Investigation
1396. Ripple effect quantification by supplier risk exposure assessment
1397. Consistency of symptom clusters in advanced cancer
1398. Cancer symptom clusters: old concept but new data
1399. Enhancing self-compassion using a Gestalt two-chair intervention
1400. Pax7 shows higher satellite cell frequencies and concentrations within intrafusal fibers of muscle spindles
1401. A Novel Three-Phase Multilevel Inverter Cascaded by Three-Phase Two-Level Inverter and Two Single-Phase Boosted H-Bridge Inverters
1402. Methodological and statistical characteristics of meta-analyses on spinal cord stimulation for chronic pain: a systematic review
1403. Social empathy and associated factors among nurses: A mixed method study
1404. What is the evidence on efficacy of spinal cord stimulation in (subgroups of) patients with critical limb ischemia?
1405. Comparison of calcaneal fixation of a retrograde intramedullary nail with a fixed-angle spiral blade versus a fixed-angle screw
1406. Intraoperative mechanical bone strength determination in tibiotalocalcaneal fusion: A biomechanical investigation
1407. Daily socket comfort in transtibial amputee with a vacuum-assisted suspension system: study protocol of a randomized, multicenter, double-blind multiple N-of-1 trial
1408. Selfâ€connection and wellâ€being: Development and validation of a selfâ€connection scale
1409. The Role of Self-Connection in the Relationship between Mindfulness and Meaning: A Longitudinal Examination
1410. Analysis, design and implementation of fuzzy logic controlled quasi-resonant zero-current switching switched-capacitor bidirectional converter
1411. Psychometric properties of the Turkish version of the Beliefs About Emotions Questionnaire (BAEQ) and a preliminary investigation in relation to emotion regulation
1412. Psychometric characteristics of the short Czech version of the five facet mindfulness questionnaire
1413. Corrigendum on: A validation study of the self-compassion scale-short form (SCS-SF) with ant colony optimization in a Turkish sample*, (Psihologija, 56(1), (89â€“114), (10.2298/PSI211127015K))
1414. A validation study of the Self-Compassion Scaleâ€“Short Form (SCS-SF) with ant colony optimization in a Turkish sample
1415. Development of the Difficulties in Emotion Regulation Scale Short Form (DERS-SF) with metaheuristic algorithms
1416. Effects of leaf quality and microhabitat on the survival of a leaf-rolling weevil (Attelabidae)
1417. Age estimation by evaluating median palatine suture closure using postmortem CT
1418. A limited panel of immunomarkers can reliably distinguish between clear cell and high-grade serous carcinoma of the ovary
1419. Supply chain resilience: a systematic literature review and typological framework
1420. Conclusion: Future Implications of ASEANâ€™s Institutional Strategies
1421. The Concept of Institutional Strategy and Change
1422. Development of the stress-induced cognition scale
1423. Measuring school climate: An overview of measurement scales
1424. On the possibilities of watershed parameterization for extreme flow estimation in ungauged basins
1425. A high gain soft-switching active-clamped coupled-inductor-based converter for grid-tied photovoltaic applications
1426. Peripheral nerve tissue engineering
1427. Effects of Cognitively Based Compassion Training in the outskirts: A mixed study
1428. A new control strategy for interfacing battery supercapacitor storage systems for PV system
1429. DC Grid Voltage Regulation Using New HESS Control Strategy
1430. Self-construal in Chile and Norway: Implications for cultural differences in individualism and collectivism
1431. Highly Oriented Carbon Nanotube Supercapacitors
1432. Novel technique for peripheral nerve reconstruction in the absence of an artificial conduit
1433. Seismic reflectivity inversion using spectral compressed sensing
1434. A method of deriving time-variant distributed unit hydrograph
1435. Geomechanical upscaling methods: Comparison and verification via 3D printing
1436. Numerical simulation of the response of non-composite steel-concrete-steel sandwich panels to impact loading
1437. Revised version of sport courage scale for children
1438. Courage in Competition: Adaptation of the Sports Courage Scale for American English and Validation of the Factor Structure with Student-Athletes at Clemson University
1439. Meta-analysis of the Mantel-Haenszel index for the detection of Differential Item Functioning
1440. Characterization and Schwann Cell Seeding of up to 15.0 cm Long Spider Silk Nerve Conduits for Reconstruction of Peripheral Nerve Defects
1441. Time-optimal feedrate scheduling with actuator constraints for 5-axis machining
1442. Inherent stiffness of three different posterior transpedicular fixation instrumentation. A mechanical comparative study
1443. Sex differences in humor experiences in relationship to compassion for oneself and for others
1444. Effectiveness of mindfulness-based cognitive therapy for improving subjective and eudaimonic well-being in healthy individuals: A randomized controlled trial
1445. Roles of positive psychology for mental health in UK social work students: Self-compassion as a predictor of better mental health
1446. Self-compassion, mental health shame and work motivation in German and Japanese employees
1447. Mental health of UK university business students: Relationship with shame, motivation and self-compassion
1448. Mental health shame of UK construction workers: Relationship with masculinity, work motivation, and self-compassion
1449. Mental health of therapeutic students: Relationships with attitudes, self-criticism, self-compassion, and caregiver identity
1450. Positive psychology for mental wellbeing of UK therapeutic students: Relationships with engagement, motivation, resilience and self-compassion
1451. Cross-cultural comparison of mental health between german and south african employees: Shame, self-compassion, work engagement, and work motivation
1452. Mental health of medical workers in Japan during COVID-19: Relationships with loneliness, hope and self-compassion
1453. Pathways to sex addiction: Relationships with adverse childhood experience, attachment, narcissism, self-compassion and motivation in a gender-balanced sample
1454. Positive psychology of Malaysian university students: Impacts of engagement, motivation, self-compassion, and well-being on mental health
1455. Cross-cultural comparison of mental health in social work students between UK and Ireland: Mental health shame and self-compassion
1456. 'Echelle de bonheur subjectif (SHS): PropriÃ©tÃ©s psychomÃ©triques de la version franÃ§aise de lâ€™Ã©chelle (SHS-F) et ses relations avec le bien-Ãªtre psychologique, lâ€™affect et la dÃ©pression' = 'Subjective Happiness Scale (SHS): Psychometric properties of the French version of the scale (SHS-F) and its relationship to psychological well-being, affect and depression'
1457. Stress regulation in drug-resistant epilepsy
1458. Cognitive and socio-affective outcomes of project-based learning: Perceptions of Greek Second Chance School students
1459. Adult student assessment in second chance schools in Greece: Teachers' views
1460. Sub-paresthesia spinal cord stimulation reverses thermal hyperalgesia and modulates low frequency EEG in a rat model of neuropathic pain
1461. Flatness and sliding mode based controller of fuel cell and supercapacitors hybrid source
1462. Lack of body positional effects on paresthesias when stimulating the dorsal root ganglion (DRG) in the treatment of chronic pain
1463. Measuring the immeasurables: Development and initial validation of the Self-Other Four Immeasurables (SOFI) Scale based on Buddhist teachings on loving kindness, compassion, joy, and equanimity
1464. Self-compassion in depression: Associations with depressive symptoms, rumination, and avoidance in depressed outpatients
1465. Malaysian perspective on great power competition in the Indo-Pacific
1466. Reconfiguration of four legged walking robot for actuator faults
1467. Spinal cord stimulation and rehabilitation in an individual with chronic complete L1 paraplegia due to a conus medullaris injury: motor and functional outcomes at 18 months
1468. Simulation of the natural characteristics of vertical a-Si:H/Î¼c-Si:H tandem solar cells. 2. Analysis of the results and comparison with the experiment
1469. Evolutionary-based iterative local search algorithm for the Shortest Common Supersequence problem
1470. Clustering method incorporating network topology and dynamics
1471. Theta burst stimulation add on to dialectical behavioral therapy in borderline-personality-disorder: methods and design of a randomized, single-blind, placebo-controlled pilot trial
1472. Impact of smart city system on the construction industry in Sri Lanka
1473. Identifying best combination of methodologies for event-based hydrological modeling using HEC-HMS software: a case study on the Panchganga River basin, India
1474. A Large-Scale RNAi Screen Identifies SGK1 as a Key Survival Kinase for GBM Stem Cells
1475. Reliability Assessment of Safety-Critical Systems of Nuclear Power Plant using Ordinary Differential Equations and Reachability Graph
1476. Supply chain management practices, retail outlets attributes and organisational performance: a case of organised food retailers in India
1477. Hierarchical formation of Ni sulfide single walled carbon nanotubes heterostructure on tin-sulfide scaffolds via mediated SILAR process: Application towards long cycle-life solid-state supercapacitors
1478. An optimized technique for reliability analysis of safety-critical systems: A case study of nuclear power plant
1479. Critical success factors of customer involvement in greening the supply chain: An empirical study
1480. Modeling daily surface runoff, sediment and nutrient loss at watershed scale employing Arc-APEX model interfaced with GIS: a case study in Lesser Himalayan landscape
1481. Identification of rainwater harvesting sites using SCS-CN methodology, remote sensing and Geographical Information System techniques
1482. A generalized case of electromagnetic scattering from a finite number of ferromagnetic cylinders
1483. Parameter Estimation for Quantitative Dependability Analysis of Safety-Critical and Control Systems of NPP
1484. A probabilistic hazard assessment framework for safety-critical and control systems: A case study for a nuclear power plant
1485. A Vehicle-to-Vehicle Wireless Energy Sharing Scheme Using Blockchain
1486. Blood Sugar Level Indication Through Chewing and Swallowing from Acoustic MEMS Sensor and Deep Learning Algorithm for Diabetic Management
1487. Blood Sugar Level Indication Through Chewing and Swallowing from Acoustic MEMS Sensor and Deep Learning Algorithm for Diabetic Management
1488. Multi-Input Switched-Capacitor Based Cross-Connected Boosting Inverter with Soft-Charging
1489. Are compassionate and self-image goals comparable across cultures?
1490. Response of near-inertial energy to a supercritical tropical cyclone and jet in the South China Sea: Modelling study
1491. Evaluation of a school-based emotional health screening program
1492. The prognostic value of the simplified comorbidity score in the treatment of small cell lung carcinoma
1493. A confirmatory factor analysis and validation of the forms of selfâ€criticism/reassurance scale
1494. Validation and clinical implementation of commercial secondary check software with heterogeneity corrections
1495. Usefulness of lysophosphatidylcholine measurement in the cerebrospinal fluid for differential diagnosis of neuropathic pain: Possible introduction into clinical laboratory testing
1496. Mapping psychoticâ€like experiences: Results from an online survey
1497. Investigating the relationship between supply chain innovation, risk management capabilities and competitive advantage in global supply chains
1498. Targeted screening and quantification of synthetic cathinones and metabolites in hair by UHPLC-HRMS
1499. Determination of the Synthetic Cannabinoids JWH-122, JWH-210, UR-144 in Oral Fluid of Consumers by GC-MS and Quantification of Parent Compounds and Metabolites by UHPLC-MS/MS
1500. Equipment collaboration in general test languages of safety critical system
1501. Micellar electrokinetic chromatography method for the analysis of synthetic and phytocannabinoids
1502. Continuous modelling of the Bouregreg watershed (Morocco) using the HEC-HMS model
1503. Flood forecasting with a dam watershed event-based hydrological model in a semi-arid context: case study in Morocco
1504. Applying hiPSCs and Biomaterials Towards an Understanding and Treatment of Traumatic Brain Injury
1505. On the origins and evolution of qualia: An experience-space perspective
1506. Psychometric Properties of the Independent and Interdependent Self-Construal Questionnaire: Evidence From the Czech Republic
1507. Metallosupramolecular compounds based on Cu(ii)/oxalate/twisted NSSN ligands showing a new in situ S-C bond cleavage
1508. Appropriate methods for evaluating the efficiency and capacitive behavior of different types of supercapacitors
1509. Battery/Supercapacitors Combination in Uninterruptible Power Supply (UPS)
1510. Transplantation of tissue engineering neural network and formation of neuronal relay into the transected rat spinal cord
1511. Phytoplankton and Bacterial Responses to Monsoon-Driven Water Masses Mixing in the Kuroshio Off the East Coast of Taiwan
1512. Learned resourcefulness, cognitive processes, and adaptive functioning in depressed adults
1513. Travel Time and Waveform Measurements of Global Multibounce Seismic Waves Using Virtual Station Seismogram Stacks
1514. Novel design of drive and test system for TDI IRCCD
1515. Drive & test system for 288 X 4 TDI IRCCD
1516. Recovery after psychosis (RAP): A compassion focused programme for individuals residing in high security settings
1517. Impact of load models in energy management using combined approach of Voltâ€“Var control and distributed generation
1518. The role of self-compassion in mediating the effect of perfectionism on personal growth initiative in dental hygienists
1519. Attenuation performance of runoff storage basins within a moderate to steep slope urban catchment in Cebu, Philippines
1520. Self-compassion, internal, and external shame in women at risk of developing an eating disorder
1521. Experimental validation of a grid-aware optimal control of hybrid AC/DC microgrids
1522. Analytical Computation of the Sensitivity Coefficients in Hybrid AC/DC Networks
1523. The calibration of a complex watershed model in sparse data environments
1524. Evaluation of the accuracy and intra- and interobserver reliability of three manual laxity tests for canine cranial cruciate ligament ruptureâ€”An ex vivo kinetic and kinematic study
1525. Notice of Retraction: The face detection algorithm combined skin color segmentation and PCA
1526. Pedometry as an External Measure of Spinal Cord Stimulation Patient Outcomes
1527. Simultaneous Bayesian Estimation of Excitatory and Inhibitory Synaptic Conductances by Exploiting Multiple Recorded Trials
1528. Validation of an UPLC-MS/MS method for the determination of sixteen synthetic cannabinoids in human hair. Application to document chronic use of JWH-122 following a non-fatal overdose
1529. Evolution of the study coordinator role: the 28-year experience in Diabetes Control and Complications Trial/Epidemiology of Diabetes Interventions and Complications (DCCT/EDIC)
1530. Self-concealment: Integrative review and working model
1531. Modeling of prestressed concrete bridge girders
1532. Constitutive models of concrete structures subjected to seismic shear
1533. Simulation of post-tensioned bridge columns under reversed-cyclic loads
1534. The relationship between mindfulness and self-compassion for self-assessed competency and self-efficacy of psychologists-in-training
1535. Written records of historical tsunamis in the northeastern South China Sea - Challenges associated with developing a new integrated database
1536. Application of a neuro-fuzzy network for gait event detection using electromyography in the child with cerebral palsy
1537. Multidimensional longest common subsequence discovery from large database using DNA operations
1538. Human epithelial stem cells persist within tissue-engineered skin produced by the self-assembly approach
1539. Evaluation of a compassionate mind group for self-harming behaviour within a secondary mental health service
1540. Quality parameters and pattern recognition methods as a tool in tracing the regional origin of multifloral honey
1541. A novel induced pluripotent stem cell model of Schwann cell differentiation reveals NF2 - related gene regulatory networks of the extracellular matrix
1542. Energy consumption estimation in different climates of a solar combisystem combined with an absorption chiller
1543. Numerical and experimental results of a novel and generic methodology for energy performance evaluation of thermal systems using renewable energies
1544. Development and factor structure of the French version of the Self-Compassion Scale Short Form (SCS-SF-FV)
1545. On the existence of optimal solutions for the modular supervisory control of hybrid systems
1546. Latent thermal energy storage for solar driven cooling systems
1547. Global approach test improvement using a neural network model identification to characterise solar combisystem performances
1548. Unraveling heterogeneities in mindfulness profiles: A review and latent profile analysis of the Five Facet Mindfulness Questionnaire Short-Form (FFMQ-SF) in the Spanish population
1549. Dorsal column steerability with dual parallel leads using dedicated power sources: A computational model
1550. Dorsal column steerability with dual parallel leads using dedicated power sources: a computational model
1551. Predicted effects of pulse width programming in spinal cord stimulation: a mathematical modeling study
1552. A Preliminary Study of the Validation for Childrenâ€™s Social Communication Scale for Children Aged 6-7 Years
1553. Experimental and numerical studies on the design of a sonic crystal window
1554. Novel plenum window with sonic crystals for indoor noise control
1555. Design of a Vaccine Passport Validation System Using Blockchain-based Architecture: Development Study
1556. The Revised Curve Number Rainfallâ€“Runoff Methodology for an Improved Runoff Prediction
1557. Therapeutic Sources of Skeletal Muscle Regeneration from Volumetric Muscle Loss: A Narrative Review
1558. Coculture of Chondrocytes and Stem Cells: A Review of Head and Neck Cell Lines for Cartilage Regeneration
1559. Exploring flavor perception through metabolite profiling and sensory approaches during starter kimchi fermentation
1560. Switched-Capacitor-Based Five-Level T-Type Inverter (SC-5TI) with Soft-Charging and Enhanced DC-Link Voltage Utilization
1561. Psychometrics and comparison of the Compulsive Sexual Behavior Inventory and the Sexual Compulsivity Scale in a male college student sample
1562. Predicting survival in pulmonary arterial hypertension in the UK
1563. QSAR Model for Predicting the Cannabinoid Receptor 1 Binding Affinity and Dependence Potential of Synthetic Cannabinoids
1564. A Novel Adaptive Digital Filter-Based Energy Management Strategy Applied to Hybrid Energy Storage System for Electric Vehicles
1565. Topological properties of finite-size heterostructures of magnetic topological insulators and superconductors
1566. Effects of electrical stimulation on the myelination of dorsal root ganglia and Schwann cells
1567. Estimating Point and Nonpoint Source Pollutant Flux by Integrating Various Models, A Case Study of the Lake Hawassa Watershed in Ethiopiaâ€™s Rift Valley Basin
1568. Antenatal mobile-delivered mindfulness-based intervention to reduce perinatal depression risk and improve obstetric and neonatal outcomes: A randomized controlled trial
1569. Are contemplative capacities created equal?: Examining demographic differences in adolescentsâ€™ contemplative capacity and differences in psychological well-being
1570. ENSO-based probabilistic forecasts of Marchâ€“May U.S. tornado and hail activity
1571. Artifactual hyperpolarization during extracellular electrical stimulation: Proposed mechanism of high-rate neuromodulation disproved
1572. Adaptation and Validation of a Spanish-Language Version of the Self-Concealment Scale
1573. AdaptaciÃ³n y ValidaciÃ³n en EspaÃ±ol de la Self-Concealment Scale = Adaptation and validation of a Spanish-language version of the Self-Concealment Scale
1574. An internal intrusion detection and protection system by using data mining and forensic techniques
1575. Assessment of contamination and origin of metals in mining affected river sediments: A case study of the Aries River catchment, Romania
1576. A study on the development of the smart classroom scale
1577. Finite element analysis on enhanced C-channel connectors in SCS sandwich composite structures
1578. A GIS-based method for depicting the characteristics of mesoscale eddies: A case study in the Northern South China Sea
1579. Range profile analysis of buried objects in layered media based on efficient full-wave simulation
1580. Street Community-Level Urban Flood Risk Assessment Based on Numerical Simulation
1581. Achieving versatile optoelectronic applications of environment-friendly halide double perovskite by multi-ion codoping strategy
1582. Construction of nitrogen-doped graphene quantum dot embedded NiGa layered double hydroxide for high-performance asymmetric supercapacitors
1583. Statistical Models of Sea Surface Salinity in the South China Sea Based on SMOS Satellite Data
1584. A physics-based atmospheric and BRDF correction for Landsat data over mountainous terrain
1585. Forward numerical investigation of potential tsunami deposits in the South China sea: A case study of Nan'ao Island
1586. Construction of small-sized blood vessel using SCs in vitro
1587. A new SCS method to improve power combiner efficiency in LINC system
1588. Performance Evaluation of Urban Intersection C-V2X Simulation System
1589. The Dependency of Probabilistic Tsunami Hazard Assessment on Magnitude Limits of Seismic Sources in the South China Sea and Adjoining Basins
1590. Compositional and structural characterization of dorsal root ganglion neurons and co-cultured Schwann cells by confocal Raman microspectral imaging
1591. Perovskite Single Crystals: Synthesis, Optoelectronic Properties, and Application
1592. Adjusting Distribution of Multiwall Carbon Nanotubes in Poly(L-lactide)/Poly(oxymethylene) Blends via Constructing Stereocomplex Crystallites: Toward Conductive and Microwave Shielding Enhancement
1593. Effects of runoff generation methods and simulation time steps on flood simulation: a case study in Liulin experimental watershed
1594. Comparison of remote sensing data with in-situ wind observation during the development of the South China Sea monsoon
1595. Wind onset and withdrawal of Asian summer monsoon and their simulated performance in AMIP models
1596. The difference of RRS product derived from MODIS MERIS and SeaWiFS in South China Sea
1597. Effects of a mindfulness-based intervention on fertility quality of life and pregnancy rates among women subjected to first in vitro fertilization treatment
1598. Vertically Oriented and Interpenetrating CuSe Nanosheet Films with Open Channels for Flexible All-Solid-State Supercapacitors
1599. Skin precursor-derived Schwann cells accelerate in vivo prevascularization of tissue-engineered nerves to promote peripheral nerve regeneration
1600. Chiral MoS2@BC fibrous membranes selectively promote peripheral nerve regeneration
1601. Matrilin-2 within a three-dimensional lysine-modified chitosan porous scaffold enhances Schwann cell migration and axonal outgrowth for peripheral nerve regeneration
1602. Phased Array Scattering-Cross-Section Reduction Based on Hybrid Scattering Cancellation Technique
1603. Low-SCS Microstrip Thinned Array
1604. Low-Scattering-Cross Section Thinned Phased Array Antenna Based on Active Cancellation Technique
1605. In-Band SCS Reduction of Microstrip Phased Array Based on Impedance Matching Network
1606. Enriched carbon dots/graphene microfibers towards high-performance micro-supercapacitors
1607. Knowledge mapping of Schwann cells: a bibliometric investigation (2012-2022)
1608. Stability of Symptom Clusters in Children With Acute Lymphoblastic Leukemia Undergoing Chemotherapy
1609. Symptom Clusters in Children With Leukemia Receiving Chemotherapy: A Scoping Review
1610. Correction of the Residual Effect From Solar Beta Angle for Onboard Calibration of Satellite Calibration Spectrometer
1611. Porosity and tortuosity: Keys for accurate modeling of porous electrodes in supercapacitors
1612. Suppression to Angular Oscillation among Synchronous Generators by Optimizing Parameters and Set-Points of Synchronous Condenser and High-Voltage DC
1613. Entrapment as a mediator of suicide crises
1614. Geographical distribution of coral reefs and their responses to environmental factors in the South China Sea
1615. Source identification and co-occurrence patterns of major elements in South China Sea sediments
1616. Prediction of bacterial species richness in the South China Sea slope sediments
1617. Capacitive energy storage from single pore to porous electrode identified by frequency response analysis
1618. The impact of acute and chronic aerobic and resistance exercise on stem cell mobilization: A review of effects in healthy and diseased individuals across different age groups
1619. Emerging senolytic agents derived from natural products
1620. Distribution of clastic minerals of surface sediments in the western China Sea and their provenance
1621. Distinct Bottom-Water Bacterial Communities at Methane Seeps With Various Seepage Intensities in Haima, South China Sea
1622. Organophosphorus pesticides in southeastern China marginal seas: Land-based export and ocean currents redistribution
1623. Preparation of amphiphilic poly(divinylbenzene-co-N-vinylpyrrolidone)-functionalized polydopamine magnetic nanoadsorbents for enrichment of synthetic cannabinoids in wastewater
1624. Effects of mindfulness-based intervention to improve bracing compliance in adolescent idiopathic scoliosis patients: A randomized controlled trial
1625. Comparative Study of Field Modulation Effects in Consequent-Pole PM Machines With Different Stator Slot Configurations
1626. Optimization of scs model to estimate runoff in the mid-southern hilly region of shandong province and evaluation of applying it
1627. Occurrence of polycyclic aromatic hydrocarbons (PAHs) in coral reef fish from the South China Sea
1628. Combined strategy of endothelial cells coating, Sertoli cells coculture and infusion improves vascularization and rejection protection of islet graft
1629. A daily diary study of the relationships among daily selfâ€compassion, perceived stress and healthâ€promoting behaviours
1630. Identification of NR0B1 as a novel androgen receptor co-repressor in mouse Sertoli cells
1631. An Optimized SCS-CN Method to Calculate Runoff over Different Underlying Surfaces in Yanqing District of Beijing
1632. Geoacoustic inversion for sediments in the South China Sea based on a hybrid inversion scheme
1633. The relationship between the interictal epileptiform discharge source connectivity and cortical structural couplings in temporal lobe epilepsy
1634. Rotation-invariant nonrigid point set matching in cluttered scenes
1635. 3D parametric channel estimation for multi-user massive-MIMO OFDM systems
1636. Piezo1 promotes peripheral nerve fibrotic scar formation through Schwann cell senescence
1637. Influence of the temperature gradient and the pulling velocity on solidification cracking susceptibility during welding: A phase field study
1638. Experimental and modelling evaluations of sulfide formation in a mega-sized deep tunnel sewer system and implications for sewer management
1639. Constructing supercapacitors with biopolymer bearing zwitterion as hydrogel electrolyte and binder for superior performance at âˆ’40 Â°C
1640. Relationship between sexual compulsivity and sexual risk behaviors among Chinese sexually active males
1641. [Estimation of urban non-point source pollution loading and its factor analysis in the Pearl River Delta]
1642. Effects of a virtual mindful self-compassion training on mindfulness, self-compassion, empathy, well-being, and stress in Uruguayan primary school teachers during COVID-19 times
1643. Impact tests on steel-concrete-steel sandwich beams with lightweight concrete core
1644. Steel-concrete-steel sandwich composite structures-recent innovations
1645. A study on self-compassion and stigmatisation in female patients with breast cancer
1646. Effects of model coupling on Typhoon Kalmaegi (2014) simulation in the South China sea
1647. Interference haptic stimulation and consistent quantitative tactility in transparent electrotactile screen with pressure-sensitive transistors
1648. Stem cell therapy for stress urinary incontinence: a critical review
1649. Validation and Variation of Upper Layer Thickness in South China Sea from Satellite Altimeter Data
1650. Millennial-scale changes in terrestrial sediment input and Holocene surface hydrography in the northern South China Sea (IMAGES MD972146)
1651. Consensus in Identification and Stability of Symptom Clusters Using Different Symptom Dimensions in Newly Diagnosed Acute Myeloid Leukemia Patients Undergoing Induction Therapy
1652. The roles of microRNAs in schwann cell myelination and myelin maintenance
1653. Unveiling the occurrence and ecological risks of organophosphate esters in seawater of the northern South China Sea
1654. Research trends in electrospun conducting polymers derived CNFs and their composite as the potential electrodes for high-performance flexible supercapacitors
1655. Satellite-observed variability of phytoplankton size classes associated with a cold eddy in the South China Sea
1656. Xinanjiang model combined with Curve Number to simulate the effect of land use change on environmental flow
1657. Partial-nodes-based state estimation for linear complex networks with randomly occurring sensor delay and stochastic coupling strength
1658. Resilience-Oriented Control for Cyber-Physical Hybrid Energy Storage Systems Using a Semiconsensus Scheme: Design and Practice
1659. A Semi-Consensus Strategy Toward Multi-Functional Hybrid Energy Storage System in DC Microgrids
1660. Topographic correction method for steep mountain terrain images
1661. Simulation of the Boreal Winter East Asian Cold Surge by IAP AGCM4.1
1662. Extremely low radioactivity in marine sediment of coral reefs and its mechanism
1663. Three-dimensional properties of mesoscale eddies in the South China Sea based on eddy-resolving model output
1664. Shear failure mechanisms of SCS sandwich beams considering bond-slip between steel plates and concrete
1665. Failure mechanism and failure patterns of SCS composite beams with steel-fiber-reinforced UHPC
1666. Ultimate capacity and failure mechanism of SCS and S-UHPC composite deep beams: Test and modeling
1667. Experimental study, finite element simulation and theoretical analysis on failure mechanism of steelâ€“concrete-steel (SCS) composite deep beams with UHPC
1668. Theoretical models and reliability assessment of steel-UHPC-steel composite beams in offshore structures
1669. Bioinformatics and validation reveal the potential target of curcumin in the treatment of diabetic peripheral neuropathy
1670. Reciprocal associations between self-compassion and eating disorder symptoms: An 8-month longitudinal study
1671. Helping the self help others: Self-affirmation increases self-compassion and pro-social behaviors
1672. A micro focus with macro impact: Exploration of initial abstraction coefficient ratio (Î») in Soil Conservation Curve Number (CN) methodology
1673. Derivation of region-specific curve number for an improved runoff prediction accuracy
1674. Inferential statistics of claim assessment
1675. Statistical and type II error assessment of a runoff predictive model in Peninsula Malaysia
1676. A multidimensional approach to perfectionism and self-compassion
1677. Not hating what you see: Self-compassion may protect against negative mental health variables connected to self-objectification in college women
1678. A Hybrid TWDM-RoF Transmission System Based on a Sub-Central Station
1679. Occurrence of microplastics in the seawater and atmosphere of the South China Sea: Pollution patterns and interrelationship
1680. Ground Validation Experiment and Spectral Detection Capability Evaluation of Mars Mineralogical Spectrometer (MMS) Aboard HX-1 Orbiter
1681. Corrigendum to â€œFormalizing an integrated metric system measuring performance of urban sustainability: Evidence from Chinaâ€ [Sustainable Cities and Society 79 (2022) 103702] (Sustainable Cities and Society (2022) 79, (S221067072200035X), (10.1016/j.scs.2022.103702))
1682. Genetic parameters and genome-wide association for milk production traits and somatic cell score in different lactation stages of Shanghai Holstein population
1683. Impact of variance of heterogeneous spectrum on performance of Cognitive Radio Ad Hoc Networks
1684. One-pot molten salt-assisted synthesis of nitrogen-doped mesoporous carbon for high energy density supercapacitors
1685. Pore-structure control of porous carbon electrode materials and energy-storage performance in water-in-salt electrolytes
1686. Estimation of PV output power in moving and rocking hybrid energy marine ships
1687. Subsurface plankton layers observed from airborne lidar in Sanya Bay, South China Sea
1688. Scattering center modelling from geometrical model of target
1689. Evaluation of ERA5 Wave Parameters with In Situ Data in the South China Sea
1690. A Low Profile, Dual-Band, Dual-Polarized Patch Antenna with Antenna-Filter Functions and Its Application in MIMO Systems
1691. Analyzing Critical Influencing Factors of the Maturity of Smart Construction Site Applications
1692. The significance of phytoplankton photo-adaptation and benthic-pelagic coupling to primary production in the South China Sea: Observations and numerical investigations
1693. Inter-annual variation of chlorophyll in the northern South China Sea observed at the SEATS Station and its asymmetric responses to climate oscillation
1694. Defect engineered Ti3C2Tx MXene electrodes by phosphorus doping with enhanced kinetics for supercapacitors
1695. Complexes discovery from weighted protein-protein interaction networks
1696. Trans-Pacific whole mantle structure
1697. An empirical investigation of information sharing behavior on social commerce sites
1698. Seismic noise in South China Sea: High-quality time-frequency analysis from 0.01 to 125 Hz
1699. An optimization approach for multi-echelon supply chain viability with disruption risk minimization
1700. A signomial programming-based approach for multi-echelon supply chain disruption risk assessment with robust dynamic Bayesian network
1701. Full-depth profiles of PAHs in the Western South China Sea: Influence of Upwelling and Mesoscale Eddy
1702. Modelling of bridging toughening in fiber-reinforced composites
1703. nfluence of different topographic correction methods on the remote sensing extraction of Robinia pseudoacacia distribution
1704. Multilayer anti-reflective coating with ultra-low refractive index SiO2 nanopillars for high efficiency multi-junction GaAs solar cells
1705. Structured compressive sensing based narrowband interference mitigation for vehicular communications
1706. Impulsive noise cancellation for MIMO-OFDM PLC systems: A structured compressed sensing perspective
1707. Structured compressed sensing based narrowband interference elimination for in-home power line communications
1708. Structured-Compressed-Sensing-Based Impulsive Noise Cancelation for MIMO Systems
1709. Association between the biophysical environment in coastal south china sea and large-scale synoptic circulation patterns: The role of the northwest pacific subtropical high and Typhoons
1710. Numerical simulation of the Kuroshio intrusion into the South China Sea by a passive tracer
1711. "Marine ontology" and marine territorial governance in South China Sea
1712. Sponge Supercapacitor rule-based energy management strategy for wireless sensor nodes optimized by using dynamic programing algorithm
1713. Large-Signal Stability Analysis for Islanded DC Microgrids with n+1 Parallel Energy-Storage Converters
1714. From self to others: Examining the association between self-compassion and prosocial behavior in Chinese adolescents using latent profile analysis
1715. Sediment sources and dispersion on the western sunda shelf, Malay Peninsula, Southern South China sea
1716. Source Localization Using Gradient Boosting Decision Tree with a Single Hydrophone in Deep Ocean
1717. Discarded Polyimide Film-Derived Hierarchical Porous Carbon Boosting the Energy Density of Supercapacitors in Na2SO4and Spiro-(1,1â€²)-bipyrrolidinium Tetrafluoroborate Electrolytes
1718. Development and internal validation of a Wasp Sting Severity Score to assess severity and indicate blood purification in persons with Asian wasp stings
1719. Fe3+ Cross-Linked Polyaniline/Cellulose Nanofibril Hydrogels for High-Performance Flexible Solid-State Supercapacitors
1720. Production of acrylic acid and propionic acid by constructing a portion of the 3-hydroxypropionate/4-hydroxybutyrate cycle from Metallosphaera sedula in Escherichia coli
1721. Real-time non-intrusive appliance load monitoring under supply voltage fluctuations
1722. Barriers to achieving sustainability in pharmaceutical supply chains in the post-COVID-19 era
1723. Validation of a new scale for the evaluation of sialorrhea in patients with Parkinson's disease
1724. Psychometric Properties of the Self-Compassion Scale-Short Form: Study of Its Role as a Protector of Spanish Nurses Professional Quality of Life and Well-Being during the COVID-19 Pandemic
1725. Validation of three predictive models for suboptimal cytoreductive surgery in advanced ovarian cancer
1726. Identification of main factors affecting mechanical characteristics of silicon carbide sludge-based geopolymer via experimental design and associated statistical analysis
1727. The use of style guides to support user interface design in the metro rail industry
1728. Development of a distributed artificial neural network for hydrologic modeling
1729. A school connectedness scale for use with adolescents
1730. Electrolytic exfoliation of few-layer graphene/sodium dodecylbenzenesulfonate for coin- and cylindrical-cell supercapacitor electrodes
1731. Battery-like flexible supercapacitors from vertical 3D diamond/graphite composite films on carbon cloth
1732. Self-compassion, daily spiritual experiences, and psychological well-being: A moderation analysis with Christian emerging adults
1733. Development and testing of the social capital scale for families of children with special health care needs
1734. Psychometric properties and cross-cultural equivalence of the Arabic Social Capital Scale: instrument development study
1735. Mindâ€“body practice is related to pro-environmental engagement through self-compassion and global identity rather than to self-enhancement
1736. Dysmenorrhea and related factors in Taiwanese adolescent girls
1737. Sliding-boundary-constrained cantilever structure for vibration isolation via nonlinear stiffness modulation
1738. Wide-View Association of 3D Scattering Centers Based on Muti-Manifold Clustering
1739. Global Scattering Center Representation of Target Wide-Angle Single Reflection/Diffraction Mechanisms Based on the Multiple Manifold Concept
1740. Spiritual Competency Scale: A confirmatory factor analysis
1741. Measurement equivalence of Self-Construal Scale across Han, Uygur and Mongolian races
1742. Automatic Classification of Slit-Lamp Photographs by Imaging Illumination
1743. Investigating Genetic Characteristics of Chinese Holstein Cowâ€™s Milk Somatic Cell Score by Genetic Parameter Estimation and Genome-Wide Association
1744. A Generic Digital Twin Framework for Collaborative Supply Chain Development
1745. Spatial-temporal variability of snow cover over the Amur River Basin inferred from MODIS daily snow products in recent decades
1746. Upper Ocean variations at IODP Hole U1505C in the northern South China Sea and their response to the East Asian Monsoon during the middle Miocene
1747. Nutrient transport and dynamics in the South China Sea: A modeling study
1748. A deep dive into compassion: Italian validation, network analysis, and correlates of recent compassion scales
1749. Modeling the soil response to rainstorms after wildfire and prescribed fire in mediterranean forests
1750. Body-Image Acceptance and Action Questionnaire: Its deleterious influence on binge eating and psychometric validation
1751. 10 kHz spinal cord stimulation for the treatment of chronic back and/or leg pain: Summary of clinical studies
1752. Are all classes created equal? Increasing precision of conceptual modeling grammars
1753. Comparative effects of mindfulness and support and information group interventions for parents of adults with autism spectrum disorder and other developmental disabilities
1754. Characteristics of modern pollen distribution in surface sediment samples for the northern South China Sea from three transects
1755. Characteristics of pollen in surface sediments from the southern South China Sea and its paleoclimatic significance
1756. Characteristics of surface soil pollen of northern Borneo and its paleoenvironmental significance
1757. Seismic Stratigraphic Interpretation Based on Unsupervised Validation and Spectral Clustering Sampling
1758. Sexual compulsivity and its relationship with condomless sex among unmarried female migrant workers in Shanghai, China: a cross-sectional study
1759. Validation of electronic structure methods for isomerization reactions of large organic molecules
1760. Influence of outer river flood level on drainage discharge in plain lake areas
1761. Intrinsic chiral topological superconductor thin films
1762. Metabolic characteristic profiling of 1-amino-3,3-dimethyl-1-oxobutan-2-yl-derived indole and indazole synthetic cannabinoids in vitro
1763. Valuing negative affect weakens affect-health linkages: Similarities and differences across affect valuation measures
1764. Industry 4.0 as an enabler of sustainability diffusion in supply chain: an analysis of influential strength of drivers in an emerging economy
1765. Development of CSMM-based shell element for reinforced concrete structures
1766. Predictors of future anxiety about male pattern baldness in New Zealand males
1767. Theoretical Analysis of Hybrid Metalâ€“Dielectric Nanoantennas with Plasmonic Fano Resonance for Optical Sensing
1768. Construction of hierarchical zinc cobalt sulfide@nickel sulfide core-shell nanosheet arrays for high-performance asymmetric solid-state supercapacitors
1769. Psychological functioning in a sample of long-term practitioners of mindfulness meditation
1770. Using a topographic index to distribute variable source area runoff predicted with the SCS curve-number equation
1771. On Estimating Instantaneous Temperature of a Supercapacitor String Using an Observer Based on Experimentally Validated Lumped Thermal Model
1772. Self-compassion in relation to alexithymia, empathy, and negative mood in young adults
1773. Simulation study of flash floods in Gaozhuang River basin based on HEC-HMS model
1774. The beneficial effect of ginsenoside Rg1 on Schwann cells subjected to hydrogen peroxide induced oxidative injury
1775. In vivo bioluminescent imaging of Schwann cells in a poly(DL-lactide- Îµ-caprolactone) nerve guide
1776. In vivo bioluminescent imaging of Schwann cells in a poly(DL-lactide-epsilon-caprolactone) nerve guide
1777. Imaging of structure at and near the core-mantle boundary using a generalized radon transform: 2. Statistical inference of singularities
1778. Single-cell sequencing analysis reveals development and differentiation trajectory of Schwann cells manipulated by M. leprae
1779. Sequential oxygen supply system promotes peripheral nerve regeneration by enhancing Schwann cells survival and angiogenesis
1780. Impact of mesoscale eddies on the source funnel of sediment trap measurements in the South China Sea
1781. Piezoelectric conduit combined with multi-channel conductive scaffold for peripheral nerve regeneration
1782. 3D porous PEDOT/MXene scaffold toward high-performance supercapacitors
1783. Supply chain crime-taxonomy development and empirical validation
1784. Measuring the Supply Chain Performance of the Floricultural Sector Using the SCOR Model and a Multicriteria Decision-Making Method
1785. Influence of power-law index and hybrid-nanoparticles concentrations on the behavior of non-Newtonian hybrid nanofluid inside water solar collector
1786. Exploring compassion: a meta-analysis of the association between self-compassion and psychopathology
1787. The relationships between self-compassion, attachment and interpersonal problems in clinical patients with mixed anxiety and depression and emotional distress
1788. An ICPT-supercapacitor hybrid system for surge-free power transfer
1789. An ICPT-supercapacitor technology for contactless power transfer with surge suppression
1790. Effects of Caffeine on Exertion, Skill Performance, and Physicality in Ice Hockey
1791. Exploring the potential mechanisms of action of the mindfulness-based social work and self-care programme
1792. Soft City Sensing: A turn to computational humanities in data-driven urbanism
1793. Direct quantitative analysis of the natural moisturizing factor (NMF) in the stratum corneum by direct analysis in real time mass spectrometry (DART-MS)
1794. Disruption mitigation in the semiconductors supply chain by using public blockchains
1795. Characterization and validation of point mutation in Exon 19 of calcium channel, voltage-dependent, Alpha-2/Delta subunit 1(CACNA2D1) gene and its relationship with mastitis traits in Sahiwal
1796. Impact of WRF Parameterization Schemes on Track and Intensity of Extremely Severe Cyclonic Storm â€œFaniâ€
1797. The associations of dispositional mindfulness, self-compassion, and reappraisal with symptoms of depression and anxiety among a sample of Indigenous students in Canada
1798. The prediction of academic buoyancy based on personality traits: Mediational effect of self-compassion
1799. Challenges in flood modeling over data-scarce regions: How to exploit globally available soil moisture products to estimate antecedent soil wetness conditions in Morocco
1800. Contribution in the Study and Numerical Investigation of the Flow Characteristics in a Solar Chimney
1801. Phenotypic heterogeneity in modeling cancer evolution
1802. Self-compassion and body dissatisfaction in men: Extension of the tripartite influence model
1803. Evaluating equanimity: Mindfulness, intimate safety, and relationship satisfaction among meditators
1804. Implementation of a novel nine-level double boosting multi-level inverter
1805. Gresilient supplier selection through Fuzzy Ordinal Priority Approach: decision-making in post-COVID era
1806. Swarm intelligent based metaheuristics for a bi-objective flexible job shop integrated supply chain scheduling problems
1807. Exploring connections between self-compassion, mindfulness, and social anxiety
1808. Ramp-Rate Limitation Control of Distributed Renewable Energy Sources Via Supercapacitors
1809. Â«God as a causal agentÂ»: Religious attribution in orthodox context
1810. A three-dimensional biomimetic peripheral nerve model for drug testing and disease modelling
1811. On the Role of Fermi Energy in Determining Properties of Superconductors: a Detailed Comparative Study of Two Elemental Superconductors (Sn and Pb), a Non-cuprate (MgB2) and Three Cuprates (YBCO, Bi-2212 and Tl-2212)
1812. On the Generalized BCS Equations Incorporating Chemical Potential for the Tc and the Calculation of the Coherence Length of Some Elements and Compressed H3S
1813. Modelling streamflow using the SWAT model and multi-site calibration utilizing SUFI-2 of SWAT-CUP model for high altitude catchments, NW Himalaya's
1814. Association of Psychopathology Symptoms, Self-Compassion, and Forgiveness in Patients With Pulmonary Embolism
1815. Selection Response Due to Different Combination of Antagonistic Milk, Beef, and Morphological Traits in the Alpine Grey Cattle Breed
1816. Dominance of agility in tourism value chains: evidence from India
1817. Single-tooth titanium implants produced with direct laser metal-forming technology. Results from a 1-year prospective multicenter study
1818. Trueness, precision, time-efficiency and cost analysis of chairside additive and subtractive versus lab-based workflows for manufacturing single crowns: An in vitro study
1819. Value Stream Mapping (VSM) to Evaluate and Visualize Interrelated Process-Chains Regarding Circular Economy
1820. CHK1-targeted therapy to deplete DNA replication-stressed, p53-deficient, hyperdiploid colorectal cancer stem cells
1821. International Conference on Civil Engineering: Innovative Development in Engineering Advances, ICC-IDEA 2023
1822. Biomonitoring for occupational health risk assessment (BOHRA)
1823. An Investigation of the Predictors of Comfortable and Fast Gait Speed in Community-Dwelling Older Adults
1824. How does grazing relate to body mass index, self-compassion, mindfulness and mindful eating in a student population?
1825. Mindfulness, self-compassion, and mindful eating in relation to fat and sugar consumption: An exploratory investigation
1826. Supply chain resilience capabilities in automotive and other industries: a mixed method approach
1827. New scaling relations to compute atom-in-material polarizabilities and dispersion coefficients: part 1. Theory and accuracy
1828. 20-50-day oscillation of summer Yangtze rainfall in response to intraseasonal variations in the subtropical high over the western North Pacific and South China Sea
1829. Replication and extension of the dual pathway model of disordered eating: The role of fear of negative evaluation, suggestibility, rumination, and self-compassion
1830. Decoding two-dimensional complex multicomponent separations by autocovariance function
1831. Validation of TRMM Rainfall Data on Slope Stability in Karanganyar, Indonesia
1832. The application of NIR spectroscopy with chemometric analysis for monitoring a powder blending process
1833. Prediction of fresh and ripened cheese yield using detailed milk composition and udder health indicators from individual Brown Swiss cows
1834. Ensembling end-to-end deep models for computational paralinguistics tasks: ComParE 2020 mask and breathing sub-challenges
1835. Genetic parameter estimation for major milk fatty acids in Alpine and Saanen primiparous goats
1836. The Role of Self-compassion as a Mediator Between Insomnia, Depression, and Anxiety
1837. Mindfulness profiles in a sample of self-reported sleep disturbance individuals
1838. Establishing the cross-cultural measurement equivalence of individualism-collectivism
1839. Effect of side chain length on the morphology of blends of 2,5-bis(3-alkylthiophen-2-yl)thieno[3,2-b]thiophene oligomers and fullerene derivatives
1840. Estimation of genetic parameters for production, composition and processability of milk from dairy sheep in a New Zealand flock
1841. The relationships between psychological flexibility, self-compassion, and emotional well-being
1842. Vehicle Magnetic Signature Compatibility for Inductive Loop Sensor with Various Signal Conditioning Systems
1843. Analysis, characterisation and modelling of pem fuel cells
1844. Exploring the mediator role of self-critical rumination between emotion regulation and psychopathology: A validation study of the Self-Critical Rumination Scale (SCRS) in a Spanish-speaking sample
1845. Exploring the effect of external shame on body appreciation among portuguese young adults: The role of self-compassion
1846. Axon-derived PACSIN1 binds to the Schwann cell survival receptor, LRP1, and transactivates TrkC to promote gliatrophic activities
1847. Attitudes to psychiatry and to mental illness among nursing students: Adaptation and use of two validated instruments in preclinical education
1848. Examining the importance of academic-specific self-compassion in the academic self-control model
1849. Dynamic modeling, simulation and control of hybrid energy storage system based on compressed air and supercapacitors
1850. Requirements engineering for safety-critical systems: A systematic literature review
1851. Requirements engineering for safety-critical systems: An interview study with industry practitioners
1852. Design of dielectric cloaks by scattering cancellation technique using genetic algorithms
1853. Surgical residents' perceptions of patient safety climate in Dutch teaching hospitals
1854. Impurity Effects on Caroli-de Gennes-Matricon Mode in Vortex Core in Superconductors
1855. Runoff modeling of the wadi systems for estimating flash flood and groundwater recharge potential in Southern Sinai, Egypt
1856. GDF5 as a rejuvenating treatment for age-related neuromuscular failure
1857. Estimating and modeling soil loss and sediment yield in the tata valley using empirical model musle
1858. Principal components by FTIR spectroscopy as innovative characterization technique during differentiation of pluripotent stem cells to pancreatic cells
1859. Clinical utilization of fast-acting sub-perception therapy (FAST) in SCS-implanted patients for treatment of mixed pain
1860. Increasing smart city competitiveness and sustainability through managing structural capital
1861. Advancing the assessment of compassion: Psychometric study of the compassion motivation and action scales in a Portuguese sample
1862. Initiation of Supporting Cell Activation for Hair Cell Regeneration in the Avian Auditory Epithelium: An Explant Culture Model
1863. Relationship among symptom clusters, quality of life, and treatment-specific optimism in patients with cancer
1864. Psychometric properties of Self-Compassion Scale Short Form (SCS-SF) in Spanish adolescents
1865. Modeling approaches for DC-DC converters with switched capacitors
1866. Spectrophotometric determination of the acidity constants of calcon in water and mixed water-organic solvents
1867. Modeling iPSC-derived human neurofibroma-like tumors in mice uncovers the heterogeneity of Schwann cells within plexiform neurofibromas
1868. Unbalancing cAMP and Ras/MAPK pathways as a therapeutic strategy for cutaneous neurofibromas
1869. Evaluation of the revised Sense of Coherence scale in a sample of older adults: A means to assess resilience aspects
1870. Using the Sexual Compulsivity Scale to predict outcomes of sexual behavior in young adults
1871. The contribution of self-compassion and core self-evaluations on burnout among licensed professional counselors
1872. Normothermic Ex-vivo Kidney Perfusion in a Porcine Auto-Transplantation Model Preserves the Expression of Key Mitochondrial Proteins: An Unbiased Proteomics Analysis
1873. Development and validation of the sense of competence scale - revised
1874. Mindfulness, self-compassion and wellbeing
1875. Psychosocial predictors of IVF success after one year: a follow-up study
1876. An examination of the relationship between teachers' sense of efficacy and school culture
1877. The role of suicide ideation in assessing near-term suicide risk: A machine learning approach
1878. Exploring second coordination sphere effects in nitric oxide synthase
1879. Measuring learned resourcefulness in college students: Factor structure of the self-control schedule (SCS)
1880. A model for agribusiness supply chain risk management using fuzzy logic. Case study: Grain route from Ukraine to Poland
1881. Simulating Mars: Enabling Testing of the Perseverance Rover Sampling and Caching Subsystem on Earth
1882. Cultivating compassion in medicine: a toolkit for medical students to improve self-kindness and enhance clinical care
1883. The Aarhus Neuromodulation Database
1884. The Choice of Spinal Cord Stimulation Versus Targeted Drug Delivery in the Management of Chronic Pain: Validation of an Outcomes Predictive Formula
1885. Choice of spinal cord stimulation versus targeted drug delivery in the management of chronic pain: a predictive formula for outcomes
1886. Spinal Cord Stimulation 50 Years Later: Clinical Outcomes of Spinal Cord Stimulation Based on Randomized Clinical Trials-A Systematic Review
1887. Computational and Experimental Validation of the Serviceability of CPS Actuators Under RBMK Graphite Stack Distortion Conditions
1888. Development and application of a simple hydrologic model simulation for a Brazilian headwater basin
1889. Suicide Crisis Syndrome: A systematic review
1890. Theoretical study on interactions of fluorinated organomercurials with arene and gold fragments
1891. The Mediating Role of Perceived Stress in Associations Between Self-Compassion and Anxiety and Depression: Further Evidence from Chinese Medical Workers
1892. Examining psychometric properties and measurement invariance of a Chinese version of the Self-Compassion Scale - Short Form (SCS-SF) in nursing students and medical workers
1893. STAMP-based analysis of deepwater well control safety
1894. Shear resistance and deflection prediction of steelâ€“concreteâ€“steel sandwich panel with headed stud connectors
1895. Stakeholder Influence on Sustainable Supply Chain Management: A Case Study of a German Apparel Frontrunner
1896. Factor structure, reliability, and validity of the revised Suicide Crisis Inventory in major depression: A multicentric Indian study
1897. Lifestyle behaviours predicting major cardiovascular diseases mortality in a practically extinct cohort of middle-aged men followed-up for 61 years
1898. Can we still learn from the Seven Countries Study?
1899. Psychopathological symptoms associated with synthetic cannabinoid use: a comparison with natural cannabis
1900. Diversity among Latino/a college students and its impact on student organization involvement
1901. Modelling of river flow in ungauged catchment using remote sensing data: application of the empirical (SCS-CN), Artificial Neural Network (ANN) and Hydrological Model (HEC-HMS)
1902. Trait-level differences in constructs that underpin the contextual behaviour therapies between binge-eating disorder with and without overvaluation of weight and shape
1903. Self-compassion and fear of self-compassion: Mechanisms underlying the link between child maltreatment severity and psychological distress in college women
1904. Self-blame and social constraints in a treatment-seeking suicide bereaved sample
1905. The Impact of the Coronavirus Pandemic on Supply Chains and Their Sustainability: A Text Mining Approach
1906. The influence of mindfulness, selfâ€compassion, psychological flexibility, and posttraumatic stress disorder on disability and quality of life over time in war veterans
1907. Predictors of recovery from post-deployment posttraumatic stress disorder symptoms in war veterans: The contributions of psychological flexibility, mindfulness, and self-compassion
1908. A bulk micromachined distributed digital microwave phase shifter with butterfly multilayer bridges and MAM capacitors
1909. Toward accurate and robust 2-D/3-D registration of implant models to single-plane fluoroscopy
1910. The impact of asymmetry on performance in different collaboration and integration environments in supply chain management
1911. Can asymmetry impact performance, collaboration & integration? An empirical study
1912. Electrical Ground Support Equipment for the Sampling Caching System of the Mars 2020 Rover
1913. Analysis of the implementation semester credit system with a scientific approach to the biology learning process
1914. Dimensionality and measurement invariance of the social safeness and pleasure scale in adolescents from community and residential youth care
1915. A Czech version of the Overall Anxiety Severity and Impairment Scale (OASIS): Standardization and psychometric properties
1916. An HPLC method for the determination of digoxin in dissolution samples
1917. Student and teacher perceptions of school social climate and attitudes toward bullying: Implications for intervention
1918. Watershed responses in arid environments
1919. Maternal and paternal emotion socialization relates to adolescent self-compassion
1920. Transfer Functions of a Spinal Cord Stimulation System in Mixed Media and Homogeneous Media for Estimation of RF Heating during MRI Scans
1921. Transfer Functions of a Spinal Cord Stimulation System in Mixed Media and Homogeneous Media for Estimation of RF Heating during MRI Scans
1922. Proposal for selection criteria of secondary cytoreductive surgery in recurrent epithelial ovarian, tubal, and peritoneal cancers
1923. Modeling Stray Capacitances of High-Voltage Capacitive Dividers for Conventional Measurement Setups
1924. The structure and measurement of self-construals: A cross-cultural study of the Self-Construal Scale
1925. Evaluation of infiltration models with different numbers of fitting parameters in different soil texture classes
1926. Wave climate simulation for southern region of the South China Sea
1927. Is intrathoracic rib plate fixation advantageous over extrathoracic plating? A biomechanical cadaveric study
1928. Assessing the Adaptation of Internet of Things (IoT) Barriers for Smart Cities' Waste Management Using Fermatean Fuzzy Combined Compromise Solution Approach
1929. Mitigation on self-discharge behaviors via morphological control of hierarchical Ni-sulfides/Ni-oxides electrodes for long-life-supercapacitors
1930. Estimation of design runoff curve numbers for Narmada watersheds (India)
1931. Assessment of design runoff curve number for a watershed
1932. Hysteresis-based analysis of overland metal transport
1933. An efficient clustering mechanism towards large scale service composition in IoT
1934. Long-term hydrological simulation based on the Soil Conservation Service curve number
1935. Validity and extension of the SCS-CN method for computing infiltration and rainfall-excess rates
1936. Recent public health concerns of the high-altitude tribal population of Lahaul and Spiti, Himachal Pradesh
1937. Perceived clinical utility of the suicide crisis syndrome: A multisite pilot study
1938. Comparing mirror neuron system activity between sporadic and familial cases of schizophrenia
1939. Social Media Addiction, Self-Compassion, and Psychological Well-Being: A Structural Equation Model
1940. Epiphytic lichen Flavoparmelia caperata as a sentinel for trace metal pollution
1941. Degeneracy in the robust expression of spectral selectivity, subthreshold oscillations, and intrinsic excitability of entorhinal stellate cells
1942. Heterogeneous stochastic bifurcations explain intrinsic oscillatory patterns in entorhinal cortical stellate cells
1943. Biplane fluoroscopy-guided percutaneous spinal cord stimulation
1944. [Development the Japanese of the Self-Compassionate Reactions Inventory]
1945. Development of the Japanese version of the Self-Compassionate Reactions Inventory
1946. Application of time domain reflectometry to high suspended sediment concentration measurements: Laboratory validation and preliminary field observations in a steep mountain stream
1947. SCS: A case study on service composition in automobile supply chain
1948. Application of trajectory clustering and regionalization to ocean eddies in the South China Sea
1949. Simulation of three dimensional elevator system using cell-DEVS formalism
1950. Application of RT-DEVS in military
1951. The Sampling and Caching Subsystem (SCS) for the Scientific Exploration of Jezero Crater by the Mars 2020 Perseverance Rover
1952. A new tool for rating cognitive behavioural supervisionâ€”Preliminary findings in a clinical setting
1953. Pain medication tapering for patients with Persistent Spinal Pain Syndrome Type II, treated with Spinal Cord Stimulation: A RCT- study protocol of the PIANISSIMO study
1954. Studying the Mediating Role of Psychological Flexibility and Self-Compassion in the Relationship between Traumatic Memories of Shame and Severity of Depression and Anxiety Symptoms
1955. Analyte protectant approach to protect amide-based synthetic cannabinoids from degradation and esterification during GCâ€“MS analysis
1956. 3rd International Conference on Green Environmental Engineering and Technology, IConGEET 2021
1957. Formation of graphene-wrapped multi-shelled NiGa2O4 hollow spheres and graphene-wrapped yolk-shell NiFe2O4 hollow spheres derived from metal-organic frameworks for high-performance hybrid supercapacitors
1958. Weighted Single-Step Genome-Wide Association Study Uncovers Known and Novel Candidate Genomic Regions for Milk Production Traits and Somatic Cell Score in Valle del Belice Dairy Sheep
1959. Introducing a system dynamicâ€“based model of quality estimation for construction industry subcontractorsâ€™ works
1960. Conceptualising a supply and demand resilience methodology: A hybrid DEMATEL-TOPSIS-possibilistic multi-objective optimization approach
1961. Would your company's resilience be internally viable after COVID-19 pandemic disruption?: A new PADRIC-based diagnostic methodology
1962. An intelligent navigational strategy for mobile robots in uncertain environments using smart cuckoo search algorithm
1963. Evaluation of official tropical cyclone landfall forecast issued by india meteorological department
1964. Consolidation of LVFRT capabilities of microgrids using energy storage devices
1965. Molecular mechanisms of Schisandra chinensis in treating depression-neuropathic pain comorbidity by network pharmacology and molecular docking analysis
1966. Critical success factors for a circular economy: Implications for business strategy and the environment
1967. Age-Correlated Phenotypic Alterations in Cells Isolated From Human Degenerated Intervertebral Discs With Contained Hernias
1968. Thermal decomposition of cardboard wastes using steam gasification
1969. Mountain lake embankment slope stabilization by vertical vibrated stone columns, highway 1-San Francisco Bay area
1970. Landuse change prediction and its impact on surface run-off using fuzzy C-mean, markov chain and curve number methods
1971. A Five-Level Switched-Capacitor Based Transformerless Inverter With Boosting Capability for Grid-Tied PV Applications
1972. Two-Dimensional Core-Shell Structure of Cobalt-Doped@MnO2 Nanosheets Grown on Nickel Foam as a Binder-Free Battery-Type Electrode for Supercapacitor Application
1973. Human Schwann Cells in vitro III. Analytical Methods and a Practical Approach for Quality Control
1974. The properties of human Schwann cells: Lessons from in vitro culture and transplantation studies
1975. Beyond the profit motive: Environmentally conscious (re)design of supply chain structures
1976. 'Self-criticism: A measure of uncompassionate behaviors toward the self, based on the negative components of the Self-Compassion Scale': Corrigendum
1977. Burnout subtypes and absence of self-compassion in primary healthcare professionals: A cross-sectional study
1978. Application of a rapid Î¼-SPE clean-up for multiclass quantitative analysis of sixteen new psychoactive substances in whole blood by LCâ€“MS/MS
1979. Application of a rapid mu-SPE clean-up for multiclass quantitative analysis of sixteen new psychoactive substances in whole blood by LC-MS/MS
1980. Design and control of a modular multilevel DC/DC converter for regenerative applications
1981. Assessing catastrophic thinking associated with debilitating mental health conditions
1982. Relationships between preschool teacher cultural beliefs and classroom practices
1983. An integrated principal component analysis and multi-objective mathematical programming approach to agile supply chain network design under uncertainty
1984. Power Conversion Technologies for a Hybrid Energy Storage System in Diesel-Electric Locomotives
1985. Noninvasive Reflection Spectroscopy Measurement of Skin Carotenoid Score in Infants Is Feasible and Reliable
1986. Noninvasive Reflection Spectroscopy Measurement of Skin Carotenoid Score in Infants Is Feasible and Reliable
1987. Psychometric properties of the Interpersonal Mindfulness in Parenting Scale in a sample of Portuguese mothers
1988. A bifactor analysis of the Difficulties in Emotion Regulation Scale - Short Form (DERS-SF) in a sample of adolescents and adults
1989. Self-critical rumination as a mediator between attachment orientations and perceived stress: Evidence from a cross-cultural validation of the Self-Critical Rumination Scale
1990. [Complaints, needs of patients with systemic sclerosis: a better understanding for a better care]
1991. Uncovering DEVS simulation behaviour throughout the Open Provenance Model
1992. Unraveling the layers of empathy: Self-compassion as a partial mediator in male and female offendersâ€™ personal distress and aggression
1993. Measurements of Best, Worst, and Average Socket Comfort Are More Reliable Than Current Socket Comfort in Established Lower Limb Prosthesis Users
1994. Self-Compassion, Adaptive Reactions and Health Behaviours Among Adults With Prediabetes and Type 1, Type 2 and Gestational Diabetes: A Scoping Review
1995. The predictive value of self-compassion for psychological adjustment in left ventricular assist device patients: an observational study
1996. Continental Shelf Sediments of Sarawak, Malaysian Borneo
1997. Satellite ocean colour algorithm for Prochlorococcus, Synechococcus, and picoeukaryotes concentration retrieval in the South China Sea
1998. Secure context switch for private computing on public platforms
1999. High prevalence of syndemic health problems in patients seeking post-exposure prophylaxis for sexual exposures to HIV
2000. Suicide Cognitions Scale: Psychometric Support in a Community Sample Using Bifactor Modeling and Altered Item Content
2001. Applying self-compassion in sport: An intervention with women athletes
2002. The effects of customer equity and religious motivation on customer retention and switching intention: A study of the Egyptian banking sector
2003. A frequency-based linguistic approach to protein decoding and design: Simple concepts, diverse applications, and the SCS Package
2004. Reducing the administrative demands of the Science Curiosity Scale: A validation study
2005. Validation and cross-cultural robustness of the School-wide Climate Scale (SCS) across Spanish and Chilean students
2006. DEVELOPMENT AND VALIDATION OF SOCIAL COMPARISON SCALE FOR WOMEN WITH INFERTILITY IN PAKISTAN
2007. Psychometric evaluation and validation of Urdu Social Rank Scale for women with infertility in Pakistan
2008. Time perspective and self-control: metacognitive management of time is important for efficient self-regulation of behavior
2009. Phenology-adjusted dynamic curve number for improved hydrologic modeling
2010. Sexual compulsivity in heterosexual married adults: The role of sexual excitation and sexual inhibition in individuals not considered 'high-risk'
2011. Multicomponent mixed metallic hierarchical ZnNi@Ni@PEDOT arrayed structures as advanced electrode for high-performance hybrid electrochemical cells
2012. Performance analyses of effective rainfall estimation methods for accurate quantification of agricultural water footprint
2013. A protective factor against mental health problems in youths? A critical note on the assessment of self-compassion
2014. Self-Compassion Correlates of Anxiety and Depression Symptoms in Youth: A Comparison of Two Self-Compassion Measures
2015. On the Edge of Psychopathology: Strong Relations Between Reversed Self-compassion and Symptoms of Anxiety and Depression in Young People
2016. Inflexible youngsters: Psychological and psychopathological correlates of the Avoidance and Fusion Questionnaire for Youths in nonclinical Dutch adolescents
2017. Good for the Self: Self-Compassion and Other Self-Related Constructs in Relation to Symptoms of Anxiety and Depression in Non-clinical Youths
2018. The process of science: A critical evaluation of more than 15 years of research on self-compassion with the Self-Compassion Scale
2019. Self-compassion and adolescentsâ€™ positive and negative cognitive reactions to daily life problems
2020. Protection as the mirror image of psychopathology: Further critical notes on the Self-Compassion Scale
2021. Protection or Vulnerability? A Meta-Analysis of the Relations Between the Positive and Negative Components of Self-Compassion and Psychopathology
2022. What matters most? Age and gender differences in self-compassion and body attitudes among college students
2023. An examination of the 3-factor model and structural invariance across racial/ethnic groups for the FACIT-Sp: a report from the American Cancer Society's Study of Cancer Survivors-II (SCS-II)
2024. An examination of the 3-factor model and structural invariance across racial/ethnic groups for the FACIT-Sp: A report from the American Cancer Society's Study of Cancer Survivors-ll (SCS-II)
2025. Attachment style, thought suppression, self-compassion and depression: Testing a serial mediation model
2026. Supercapacitor testing for power smoothing in a variable speed offshore wave energy converter
2027. Supercapacitor testing for power smoothing in a variable speed offshore wave energy converter
2028. Occurrence of high-speed solar wind streams over the grand modern maximum
2029. Micro-and nanoscale control of the cardiac stem cell niche for tissue fabrication
2030. Making Data Valuable for Smart City Service Systems - A Citizen Journey Map for Data-driven Service Design
2031. Fostering Mental Wellness and Team Success: The Impact of Coach-Athlete Relationship and Sports Psychological Safety
2032. MicroRNA-155 contributes to plexiform neurofibroma growth downstream of MEK
2033. How can value methodology connect firms of a supply chain?
2034. Modeling transversely loaded metal-matrix composites
2035. Development of Short-Term Reservoir Level Forecasting Models: A Case Study of Ajwa-Pratappura Reservoir System of Vishwamitri River Basin of Central Gujarat
2036. Runoff estimation and identification of water harvesting structures for groundwater recharge using geo-spatial techniques
2037. Spatial mapping of runoff from a watershed using scs-cn method with remote sensing and gis
2038. Designed construction of yolk-shell structured trimanganese tetraoxide nanospheres: Via polar solvent-assisted etching and biomass-derived activated porous carbon materials for high-performance asymmetric supercapacitors
2039. Rational Construction of Bi2CuO12Se4 and VGCFs@Fe2O3 Composite Electrodes for High-Performance Semi-Solid-State Asymmetric Supercapacitors
2040. Synthesis of hierarchical ZnO/NiO nanocomposite Wurtz hexagonal nanorods via hydrothermal for high-performance symmetric supercapacitor application
2041. Spinal Cord Stimulation for Spasticity: Historical Approaches, Current Status, and Future Directions
2042. Soil carbon sequestration in tropical agroforestry systems: a feasibility appraisal
2043. Abuse, invalidation, and lack of early warmth show distinct relationships with selfâ€criticism, selfâ€compassion, and fear of selfâ€compassion in personality disorder
2044. Functionalization of graphene oxide via chromium complexes coordinated on 5-aminopyridine-2-carboxylic acid as a symmetric supercapacitor electrode materials in energy storage devices
2045. Seeking safety therapy for pathological gambling and PTSD: a pilot outcome study
2046. Connecting in-session corrective emotional experiences with postsession therapeutic changes: A systematic case study
2047. Microspectroscopy of spectral biomarkers associated with human corneal stem cells
2048. Screening Child Social-emotional and Behavioral Functioning in Low-Income African Country Contexts
2049. Stress Testing Method for Scenario-Based Testing of Automated Driving Systems
2050. Quality of care in contraceptive services provided to young people in two Ugandan districts: a simulated client study
2051. Electronic tongue for the simple and rapid determination of taste and odor compounds in water
2052. Clinical utility of squamous and transitional nuclear structure alterations induced by Schistosoma haematobium in chronically infected adults with bladder damage verified by ultrasound in Ghana
2053. Determining potential rainwater harvesting sites using a continuous runoff potential accounting procedure and GIS techniques in central Italy
2054. Impacts of hydrometeorological factors on discharge simulation in the North West Himalayas: a SUFI-2 algorithm-driven investigation using the SWAT model
2055. 19F and 1H quantitative-NMR spectroscopic analysis of fluorinated third-generation synthetic cannabinoids
2056. Supply chain resilience: conceptual model building and validation
2057. Risks to Big Data Analytics and Blockchain Technology Adoption in Supply Chains
2058. Drooling rating scales in Parkinson's disease: A systematic review
2059. Efficient charge storage by ZnCo2S4 nanoflakes@MgCo2O4 nanorods composite in Mg2+/Zn2+/K+ conducting electrolytes
2060. Automotive recall risk: impact of buyerâ€’supplier relationship on supply chain social sustainability
2061. Cross-tier interference management scheme for downlink mMIMIO-NOMA hetnet
2062. Simulation of rainfallâ€“runoff process for an ungauged catchment using an event-based hydrologic model: A case study of koraiyar basin in Tiruchirappalli city, India
2063. Development and validation of a simple thin-layer chromatographic method for the analysis of p-chlorophenol in treated wastewater
2064. Matrix stiffness determines the fate of nucleus pulposus-derived stem cells
2065. Assessing self-criticism and self-reassurance: Examining psychometric properties and clinical usefulness of the Short-Form of the Forms of Self-Criticizing/Attacking & Self-Reassuring Scale (FSCRS-SF) in Spanish sample
2066. Methodology for Surface Runoff Evaluation Using HEC-HMS in Gauged basins
2067. Detection of the Synthetic Cannabinoids AB-CHMINACA, ADB-CHMINACA, MDMB-CHMICA, and 5F-MDMB-PINACA in Biological Matrices: A Systematic Review
2068. A highly thermal-stable orange red emitting La(OH)â‚ƒ:SmÂ³âº phosphor for w-LED and thermal sensor dual-applications
2069. Maternal pandemic-related stress during pregnancy associates with infants' socio-cognitive development at 12 months: A longitudinal multi-centric study
2070. Spatio-temporal dynamics of phytoplankton functional groups in the South China Sea and their relative contributions to marine primary production
2071. Validation and psychometric evaluation of a brief screening questionnaire for psychological distress in patients with psoriasis
2072. Adaptation of the Fears of Compassion Scale into Turkish: a reliability and validity study
2073. Self-kindness when facing stress: The role of self-compassion, goal regulation, and support in college studentsâ€™ well-being
2074. Self-compassion Is Best Measured as a Global Construct and Is Overlapping with but Distinct from Neuroticism: A Response to Pfattheicher, Geiger, Hartung, Weiss, and Schindler (2017)
2075. Self-Compassion and Psychological Well-Being
2076. 'The Self-Compassion Scale is a valid and theoretically coherent measure of self-compassion': Erratum
2077. Does self-compassion entail reduced self-judgment, isolation, and over-identification? A response to Muris, Otgaar, and Petrocchi (2016)
2078. Commentary on Muris and Otgaar (2020): Let the empirical evidence speak on the Self-Compassion Scale
2079. The differential effects fallacy in the study of self-compassion: Misunderstanding the nature of bipolar continuums
2080. Development and Validation of the Self-Compassion Scale for Youth
2081. Self-compassion: What it is, what it does, and how it relates to mindfulness
2082. Self-compassion and well-being in parents of children with Autism
2083. Compassion, well-being, and the hypo-egoic self
2084. The development and validation of the State Self-Compassion Scale (long- and short form)
2085. Self-compassion versus global self-esteem: Two different ways of relating to oneself
2086. Self-compassion: Theory and measurement
2087. Self-compassion and ACT
2088. Estimation of Water Yield under Baseline and Future Climate Change Scenarios in Genale Watershed, Genale Dawa River Basin, Ethiopia, Using SWAT Model
2089. Self-Compassion in Adolescents and Young Adults With Inflammatory Bowel Disease: Relationship of Self-Compassion to Psychosocial and Physical Outcomes
2090. Calibration of an automated California mastitis test with focus on the device-dependent variation
2091. Alternative governmental carbon policies on populations of green and non-green supply chains in a competitive market
2092. Examining the roles of self-compassion and resilience on health-related quality of life for individuals with Multiple Sclerosis
2093. The relationship between perfectionism and interpersonal sensitivity with self-compassion in university students: The mediation of repetitive negative thinking
2094. V-Ag doped ZnO nanorod as high-performance electrode material for supercapacitors with enhanced specific capacitance and cycling stability
2095. Common Ground Single-Phase Single-Stage Transformerless Inverter Five Levels Using Reduced Components and Switched Capacitor Cell
2096. Common Ground Buck Type Five-Level Transformerless Inverter With Less Stress
2097. Secondary conditions in a community sample of people with spinal cord damage
2098. Mindfulness group work: Preventing stress and increasing self-compassion among helping professionals in training
2099. The effect of the vibratory surface finishing process on surface integrity and dimensional deviation of selective laser melted parts
2100. From the attributes of smart tourism technologies to loyalty and WOM via user satisfaction: the moderating role of switching costs
2101. Translation and validation of a Chinese version of the Self-Control Schedule in Chinese childbearing women
2102. Response of Sorghum bicolor genotypes for yield and yield components and organic carbon storage in the shoot and root systems
2103. Merging control of a hybrid energy storage system using battery/supercapacitor for electric vehicle application
2104. A comparative study of adaptive filtering strategies for hybrid energy storage systems in electric vehicles
2105. A Family of Sliding Mode Controllers: Analysis, Design and Comparison
2106. A cost-benefit analysis of capacitor allocation problem in radial distribution networks using an improved stochastic fractal search algorithm
2107. Fast Speed Convergent Stability of T-S Fuzzy Sliding-Mode Control and Disturbance Observer for a Secure Communication of Chaos-Based System
2108. An Optimal Homogenous Stability-Based Disturbance Observer and Sliding Mode Control for Secure Communication System
2109. â€œSelf-compassion and life satisfaction in Vietnamese adolescents: the mediating role of emotional and behavioral problemsâ€
2110. Therapeutic effect of spinal cord stimulation on neuropathic pain and its influencing factors
2111. Phylogeography of bivalve Cyclina sinensis: testing the historical glaciations and Changjiang River outflow hypotheses in northwestern Pacific
2112. Verification & validation of an agent-based forest fire simulation model
2113. Self-compassion: Implications for work-family conflict and balance
2114. The Pain-Invalidation Scale: Measuring Patient Perceptions of Invalidation Toward Chronic Pain
2115. The floating growing system and new growing systemÂ® to grow leafy vegetables and herbs
2116. Simulation and influence factor analysis of circulation and thermal structure of the surface layer of the South China Sea
2117. Compassionate and self-image goals in the United States and Japan
2118. The Multidimensional Self-Control Scale (MSCS): Development and validation
2119. Centralized control of system voltage/reactive power using genetic algorithm
2120. A Step-by-Step Design Methodology for Broadband Tunable Microwave Metasurface Absorbers Using Theory of Characteristic Modes
2121. Constrained discrete mode control of supercapacitor energy storage system for improved AGC of a multi-area power system with effects of wind power
2122. Mindfulness-based cognitive therapy intervention for young adults with cancer: A pilot mixed-method study
2123. Programmable Nanocarbon-Based Architectures for Flexible Supercapacitors
2124. TradeMap: A FINMA-compliant Anonymous Management of an End-2-end Trading Market Place
2125. Invasive stimulation therapies for the treatment of refractory pain
2126. Psychological compassion climate: Examining the nomological network of perceptions of work group compassion
2127. Psychometric Properties of the Persian Version of the Weight Control Strategies Scale (WCSS)
2128. Assessing Hydrological Response in the Timah-Tasoh Reservoir Sub-Catchments: Calibration and Validation using the HEC-HMS Model
2129. Development and assessment of a community follow-up questionnaire for the Rick Hansen spinal cord injury registry
2130. An exploration of relationship between selfâ€compassion and voiceâ€related distress in people who hear voices
2131. Sleep disturbance mediates the link between both selfâ€compassion and selfâ€criticism and psychological distress during prolonged periods of stress
2132. An inverse method for watershed change detection using hybrid conceptual and artificial intelligence approaches
2133. Stability improvement of 200Â MW Gabal El-Zayt wind farm connected to electrical grid using supercapacitor and static synchronous compensator during extreme gust
2134. Comparison of Lambertian Model on Multi-Channel Algorithm for Estimating Land Surface Temperature Based on Remote Sensing Imagery
2135. Quantitative Sensory Testing in Spinal Cord Stimulation: A Narrative Review
2136. Improving competitiveness in manufacturing-wholesaling-retailing supply chains
2137. U.S.-China strategic competition in south and east china seas: Background and issues for congress
2138. Measurement invariance of the Suicide Cognitions Scale-Revised (SCS-R)
2139. Successful long-term outcomes of spinal cord stimulation despite limited pain relief during temporary trialing
2140. Hypersexual Disorder According to the Hypersexual Disorder Screening Inventory in Help-Seeking Swedish Men and Women With Self-Identified Hypersexual Behavior
2141. Lesser degree of HR and HF-HRV recovery from an evaluative stressor is associated with higher levels of perfectionism and self-compassion
2142. Investigating the psychometric properties of Self-criticism and Self-reassurance Scale in Iranian students
2143. Taste and smell abnormalities in advanced cancer: Negative impact on subjective food intake
2144. Information assurance modeling using the Department of Defense Architecture Framework
2145. Building inter-personal competence in architecture and urban design students through smart cities at a higher education institution
2146. Molybdenum selenide nanotubes decorated carbon net for a high performance supercapacitor
2147. Measuring mindfulness in Black Americans: A psychometric validation of the Five Facet Mindfulness Questionnaire
2148. Intake of nutrients from scavengeable resources by scavenging chickens supplemented free choice with protein and energy
2149. The maturity of supply chain sustainability disclosure from a continuous improvement perspective
2150. Development and validation of a Shopping Consciousness Scale in voluntary panel Web surveys
2151. Development of a Novel Railway Positioning System Using RFID Technology
2152. Single-step genome-wide association for longitudinal traits of Canadian Ayrshire, Holstein, and Jersey dairy cattle
2153. The nomological network of cognitive fusion among people living with HIV: Associations with rumination, shame, and depressive symptoms
2154. Terrorism catastrophizing as related to self-esteem and cultural worldview in a sample of outpatient adults with pre-existing mental health disorders
2155. Genome-Wide Association Studies of Somatic Cell Count in the Assaf Breed
2156. Simultaneous analysis of 29 synthetic cannabinoids and metabolites, amphetamines, and cannabinoids in human whole blood by liquid chromatography-tandem mass spectrometry - A New Zealand perspective of use in 2018
2157. A brain for all seasons: An in vivo MRI perspective on songbirds
2158. The relationship of self-concealment to behavioral inhibition and behavioral approach
2159. U.S.-China strategic competition in South and East China seas: Background and issues for Congress (updated)
2160. The Interpersonal Mindfulness in Parenting Scale: Examining the reliability and validity in Spanish parents
2161. Demagnetization Detection and Severity Assessment in PMSMs Using Search Coils Exploiting Machine's Symmetry
2162. Upper boundary of the pacific plate subducting under Hokkaido, Japan, estimated from ScSp phase
2163. Upper boundary of the Pacific plate subducting beneath Hokkaido, Japan, estimated from ScSp phase
2164. Secretory carcinoma of the breast and its histopathological mimics: value of markers for differential diagnosis
2165. Long-term variability of extreme significant wave height in the South China Sea
2166. [Suicide risk assessment and suicide-specific syndromes]
2167. Correction to: The courage to care: Teacher compassion predicts more positive attitudes toward trauma-informed practice
2168. The Courage to Care: Teacher Compassion Predicts More Positive Attitudes Toward Trauma-Informed Practice
2169. Determination of Johnson-Cook plasticity model parameters for CoCrMo alloy
2170. 3D-microtissue derived secretome as a cell-free approach for enhanced mineralization of scaffolds in the chorioallantoic membrane model
2171. Dedifferentiated Schwann cell-derived TGF-Î²3 is essential for the neural system to promote wound healing
2172. Dedifferentiated Schwann cell-derived TGF-beta3 is essential for the neural system to promote wound healing
2173. Estimation of Flood Discharge in Ungauged Basin Using GPM-IMERG Satellite-Based Precipitation Dataset in a Moroccan Arid Zone
2174. Flood modeling through remote sensing datasets such as LPRM soil moisture and GPM-IMERG precipitation: A case study of ungauged basins across Morocco
2175. Measuring disease exacerbation and flares in rheumatoid arthritis: Comparison of commonly used disease activity indices and individual measures
2176. Machine Learning Algorithms Provide Greater Prediction of Response to SCS Than Lead Screening Trial: A Predictive AI-Based Multicenter Study
2177. Preservation of Porcine Donation after Circulatory Death (DCD) Liver by Perfusion and Orthotopic Liver Transplantation
2178. Development the Internet Usage Self-Control Scale for College Students
2179. Producing hedonic price indices for developing markets: Explicit time variable versus strictly cross-sectional models
2180. Construct Validity Study of the Turkish Form of the Short Beck Depression Inventory
2181. Quantitative flow characteristics for side-by-side square cylinders via PIV
2182. Energy Optimization in Ultra-Dense Radio Access Networks via Traffic-Aware Cell Switching
2183. A randomized controlled efficacy trial of mindfulness-based stress reduction compared with an active control group and usual care for fibromyalgia: The EUDAIMON study
2184. Impact of mindfulness and self-compassion on anxiety and depression: The mediating role of resilience
2185. Spread-spectrum vs. Quantization-based data hiding: Misconceptions and implications
2186. Reliable analysis of the single nucleotide polymorphism of lactase persistence LPH(-13910) C/T from saliva derived DNA: validation of a standardized saliva collection system
2187. Local anchor based location management schemes for small cells in hetnets
2188. Deep eutectic solvents as green and cost-effective supercapacitor electrolytes
2189. Functional Connectivity Magnetic Resonance Imaging Sequences in Patients With Postsurgical Persistent Spinal Pain Syndrome Type 2 With Implanted Spinal Cord Stimulation Systems: A Safety, Feasibility, and Validity Study
2190. Scaling factor assessment in single-step GBLUP evaluations for small genotyped populations: A case study on Iranian Holstein cattle
2191. The Effects of Irrigation Water Salinity on the Synthesis of Photosynthetic Pigments, Gas Exchange, and Photochemical Efficiency of Sour Passion Fruit Genotypes
2192. Ultrasound-guided placement of long peripheral cannulas in children over the age of 10 years admitted to the emergency department: a pilot study
2193. The mindful therapist trainee: An exploratory study of the in-session internal experiences of beginning therapists
2194. Study Protocol for a randomized controlled trial of mindfulness training with immersive technology (virtual reality) to improve the quality of life of patients with multimorbidity in Primary Care: The Mindful-VR study
2195. Assessing the Accuracy of Biogenic Content Estimation from Visible Derivative Spectroscopy of Sedimentary Cores from the Western Pacific
2196. A marine sediment component interpretation based on diffuse reflectance spectrophotometry from northern South China Sea sediment core MD972148
2197. Climatology of physical hydrographic and biological characteristics of the Northern South China Sea Shelf-sea (NoSoCS) and adjacent waters: Observations from satellite remote sensing
2198. Joint optimization of parameters of synchronous condenser excitation and step-up transformer considering the stability boundary of the power system
2199. Self-consciousness and psychological distress: A study using the Greek SCS
2200. Novel oxygenation technique for hypothermic machine perfusion of liver grafts: Validation in porcine Donation after Cardiac Death (DCD) liver model
2201. Targeting spectroscopic accuracy for dispersion bound systems from ab initio techniques: Translational eigenstates of Ne@C70
2202. Self-Balanced Switched-Capacitor Common-Grounding Boost Multilevel Inverter
2203. Industry 4.0 and supply chain sustainability: benchmarking enablers to build reliable supply chain
2204. Soil Loss Estimation Using RUSLE in Hard Rock Terrain: a Case Study of Bundelkhand, India
2205. Positive, not negative, selfâ€compassion mediates the relationship between selfâ€esteem and wellâ€being
2206. Cyber security risks in globalized supply chains: conceptual framework
2207. Delineation of potential groundwater zones based on multicriteria decision making technique
2208. ULK1 affects cell viability of goat Sertoli cell by modulating both autophagy and apoptosis
2209. Development and testing of a modified SWAT model based on slope condition and precipitation intensity
2210. Ab initio modeling of methanol interaction with single-walled carbon nanotubes
2211. Strong (Î±, k)-cut and computational-based segmentation based novel hesitant fuzzy time series forecasting model
2212. An operational method for Flood Directive implementation in ungauged urban areas
2213. Loss of Dicer in Sertoli cells has a major impact on the testicular proteome of mice
2214. Key sources of operational inefficiency in the pharmaceutical supply chain
2215. Web-based platform for eco-sustainable supply chain management
2216. Decoding the different states of visual attention using functional and effective connectivity features in fMRI data
2217. An Approach for Estimating Monthly Curve Number Based on Remotely-Sensed MODIS Leaf Area Index Products
2218. Reprograming skin fibroblasts into Sertoli cells: a patient-specific tool to understand effects of genetic variants on gonadal development
2219. Chondrogenesis of human mesenchymal stem cells encapsulated in a hydrogel construct: neocartilage formation in animal models as both mice and rabbits
2220. Band-Based Best Model Selection for Topographic Normalization of Normalized Difference Vegetation Index Map
2221. Wellbeing and distress in young people with chronic conditions: How do positive psychology variables relate to mental health outcomes?
2222. Factors associated with provision of smoking cessation support to pregnant women - a cross-sectional survey of midwives in New South Wales, Australia
2223. Investigating the impact of physical activity counselling on self-compassion and physical activity
2224. Multi-agent based sineâ€“cosine algorithm for optimal integration of DERs with consideration of existing OLTC in distribution networks
2225. Isolating stem cells in the inter-follicular epidermis employing synchrotron radiation-based Fourier-transform infrared microspectroscopy and focal plane array imaging
2226. Optimal treatment for lumbar spinal stenosis: an update
2227. Validation of the Immunization Data Reported under Health Management Information System by the Primary Health Centres of Rural Vadodara
2228. A pilot study of eight-session mindfulness-based cognitive therapy adapted for womenâ€™s sexual interest/arousal disorder
2229. PREDICTION OF RUNOFF BY SYNTHETIC UNIT HYDROGRAPH METHODS FOR THE DESIGN STORMS IN WARANA RIVER BASIN, MAHARASHTRA, INDIA
2230. Effect of Heartfulness Meditation on Oxidative Stress and Mindfulness in Healthy Participants
2231. Supercomputing centers and electricity service providers: A geographically distributed perspective on demand management in Europe and the United States
2232. Force control in single DOF dual arm cooperative space robot
2233. Understanding the charge storage mechanism of supercapacitors:: In situ / operando spectroscopic approaches and theoretical investigations
2234. A preliminary study about the prospects of extended range forecast of tropical cyclogenesis over the north Indian ocean during 2010 post-monsoon season
2235. Testing nonâ€cognitive attributes in selection centres: How to avoid being reliably wrong
2236. How effective are selection methods in medical education? A systematic review
2237. Buffering impostor feelings with kindness: The mediating role of self-compassion between gender-role orientation and the impostor phenomenon
2238. Control Strategy to Provide Frequency Support Functionality Using a Supercapacitor-Based Energy Storage System
2239. CSN1S1, CSN3 and LPL: Three Validated Gene Polymorphisms Useful for More Sustainable Dairy Production in the Mediterranean River Buffalo
2240. Comparison of lumped and quasi-distributed clark runoff models using the SCS curve number equation
2241. Hydrologic modelling of flash floods and their effects
2242. Development and validation of a fluorometric method for the determination of hesperidin in human plasma and pharmaceutical forms
2243. Ferrocene surface-modified Fe3O4 nanoparticles as prominent electrode material for supercapacitor application
2244. Lambert W function based closed-form expressions of supercapacitor electrical variables in constant power applications
2245. Optimization of supercapacitor sizing for high-fluctuating power applications by means of an internal-voltage-based method
2246. A novel analytical solution for the calculation of temperature in supercapacitors operating at constant power
2247. Derivation, validation and comparative performance of a simplified chest X-ray score for assessing the severity and outcome of pulmonary tuberculosis
2248. Verification of potential intensity relations for the northwest Indian Ocean tropical cyclones during 1990-2019
2249. Insights into stem cell therapy for premature ovarian insufficiency
2250. Mindfulness, Self-Compassion, and Acceptance as Predictors of Sexual Satisfaction in Cisgender Heterosexual Men and Women
2251. Analyzing the coastal sea level trends from SCMR-reprocessed altimeter data: A case study in the northern South China Sea
2252. Magnetic separation of peripheral nerve-resident cells underscores key molecular features of human Schwann cells and fibroblasts: an immunochemical and transcriptomics approach
2253. Floquet higher-order topological insulators and superconductors with space-time symmetries
2254. Ru Nanospheres in Water Drops for Enhanced Catalytic Performances in Selective Hydrogenation
2255. A smart conductivity sensor with temperature and water tide level compensation capabilities
2256. Fluorescent Detection of Merlin-deficient Schwann Cells and Primary Human Vestibular Schwannoma Cells Using Sodium Fluorescein
2257. Effects of a mindfulness-based intervention on psychological distress, well-being, and maternal self-efficacy in breast-feeding mothers: Results of a pilot study
2258. A proposed set of criteria for supply chain strategy evaluation
2259. An accurate analysis of scalar quantization-based data hiding
2260. Vascular catheter colonization: surveillance based on culture of needleless connectors
2261. Validation of Mazhari's Equivalent Circuit Model for Perovskites Solar Cells With S-Shaped J-V Curves
2262. An enhanced psychological mindset intervention to promote adolescent wellbeing within educational settings: A feasibility randomized controlled trial
2263. [Multislice computed tomography to detect coronary stenosis among asymptomatic patients with cardiovascular risk factors and equivocal prior stress test: preliminary study]
2264. Multislice computed tomography to detect coronary stenoses among asymptomatic patients with cardiovascular risk factors and equivocal prior stress test: Preliminary study
2265. Supply chain dynamics in the SCOR model - A simulation modeling approach
2266. Uncovering tomato candidate genes associated with drought tolerance using Solanum pennellii introgression lines
2267. Pathway-specific reporter genes to study stem cell biology
2268. Learned resourcefulness, danger in intimate partner relationships, and mental health symptoms of depression and PTSD in abused women
2269. Voltage regulation in a smart distribution system incorporating variable renewable generation
2270. Adaptation and preliminary validation evidences of the School Climate Questionnaire - Revised, Elementary and Middle School Version (SCS-MS)
2271. Invasive therapy options for chronic pain - An option after comprehensive interdisciplary assessment
2272. Fears of Compassion Scales: Cross-Cultural Adaptation and Validity Evidence for Use in Brazil
2273. The relationship between self-compassion and the risk for substance use disorder
2274. An integrated geoinformatics technology with hydrological model to impact assessment of land use change on runoff in the upper Lam ta Khong watershed
2275. Self-compassion mindsets: The components of the self-compassion scale operate as a balanced system within individuals
2276. Self-compassion: a resource for positive aging
2277. Career success in university graduates: Evidence from an Ecuadorian study in los rÃ­os province
2278. Evaluating the effectiveness of enhancing resilience in human service professionals using a retreat-based mindfulness with metta training program: A randomised control trial
2279. Integrated prediction model of patient factors, resectability scores and surgical complexity to predict cytoreductive outcome and guide treatment plan in advanced ovarian cancer
2280. Single cell RNA sequencing analysis of mouse cochlear supporting cell transcriptomes with activated ERBB2 receptor indicates a cell-specific response that promotes CD44 activation
2281. Single cell RNA sequencing analysis of mouse cochlear supporting cell transcriptomes with activated ERBB2 receptor indicates a cell-specific response that promotes CD44 activation
2282. Identification and quantification of homologous series of compound in complex mixtures: autocovariance study of GC/MS chromatograms
2283. Polska adaptacja skali konstruktÃ³w ja = Polish adaptation of the Self-Construal Scale
2284. Composite Score Is a Better Reflection of Patient Response to Chronic Pain Therapy Compared With Pain Intensity Alone
2285. Psychometric properties of the PACT Scale and relations with symptoms of PTSD
2286. Self-compassion, resilience and psychological well-being among Malaysian counsellors
2287. StemMapper: a curated gene expression database for stem cell lineage analysis
2288. Experiential avoidance in clinical and non-clinical samples: AAQ-II Portuguese version
2289. Highly accurate CCSD(T) and DFT-SAPT stabilization energies of H-bonded and stacked structures of the uracil dimer
2290. Pain medicine fellow neuromodulation surgical skill assessment tool: a pilot
2291. Pain medicine fellow neuromodulation surgical skill assessment tool: a pilot
2292. The benevolent leadership of wonder woman: Leading through love of self and humanity
2293. A systematic review of the survival and complication rates of zirconia-ceramic and metal-ceramic single crowns
2294. The Santa Clara Ethics Scale
2295. Overview and Feasibility of a Novel Transdisciplinary Integrative Approach to High Impact Chronic Pain in Vermont
2296. Quality of Life in Platinum-Sensitive Recurrent Ovarian Cancer: Chemotherapy Versus Surgery Plus Chemotherapy
2297. Displaced rotations of coherent states
2298. Validation and Psychometric Properties of the Italian Version of the Unconditional Self-Kindness Scale (USKS)
2299. Validation of the Italian version of the Neuroception of Psychological Safety Scale (NPSS)
2300. Characterisation of fibre strength distributions in matrix coated monofilament silicon carbide fibres
2301. A reduced-order model to estimate first wall particle and heat fluxes for systems codes
2302. The Compassion Scale
2303. The Development and Validation of the Compassion Scale
2304. Creating a Rainfall-Runoff Model for Measuring Streamflow from First-Order and Ungauged Headwaters of the Tropically Medium-Sized Watersheds in Northern Thailand
2305. Andreev spectroscopy of FeSe: Evidence for two-gap superconductivity
2306. Application of high-performance liquid chromatography with charged aerosol detection (LCâ€“CAD) for unified quantification of synthetic cannabinoids in herbal blends and comparison with quantitative NMR results
2307. [CRITERIA FOR SHORT AND LONG-TERM PROGNOSIS IN HOSPITALIZED PATIENTS BASED ON THE ECG SIGNAL CHARACTERISTICS AT ADMISSION. RATIONALE AND STUDY DESIGN]
2308. Axonal Growth Arrests after an Increased Accumulation of Schwann Cells Expressing Senescence Markers and Stromal Cells in Acellular Nerve Allografts
2309. Responsiveness of the International Spinal Cord Injury Quality of Life Basic Data Set V2.0: An international longitudinal study
2310. Impact of Urbanization on Runoff and Infiltration in Walnut Gulch Experimental Watershed
2311. Statistical analysis and Monte-Carlo simulation of printed supercapacitors for energy storage systems
2312. Self-compassion letter tool for healthcare worker well-being: a qualitative descriptive analysis
2313. On the use of Principal Component Analysis for reducing the number of environmental objectives in multi-objective optimization: Application to the design of chemical supply chains
2314. Event-based rainfall-runoff simulation using different precipitation loss methods: case study in tropical monsoon catchment
2315. Activity in the human superior colliculus associated with reaching for tactile targets
2316. A Compact Semi-circular Slot MIMO Antenna with Enhanced Isolation for Sub-6 GHz 5G WLAN Applications
2317. Functional P2X7 Receptors in the Auditory Nerve of Hearing Rodents Localize Exclusively to Peripheral Glia
2318. Enhancing supplier capability through Six Sigma enablers
2319. Supercapacitor Parameter Identification Using Grey Wolf Optimization and Its Comparison to Conventional Trust Region Reflection Optimization
2320. Temporal Variation of Percolation and Evapotranspiration Components in the Water Cycle for the Ropar District, Punjab, India, Using SWAT
2321. Flood hydrograph simulation to estimate peak discharge in Ciliwung river basin
2322. Interpersonal mindfulness: Scale development and initial construct validation
2323. Genetic response for milk production traits, somatic cell score, acidity and coagulation properties in Italian Holstein-Friesian population under current and alternative selection indices and breeding objectives
2324. A randomised study to evaluate the potential added value of shared meditation involving people with cancer, healthÂ professionals and third persons compared to meditation conducted with patients only: design of the Implic-2Â protocol
2325. An exploration of self-compassion in incarcerated women
2326. Computed tomography segmental calcium score (SCS) to predict stenosis severity of calcified coronary lesions
2327. Automated ISH for Validated Histological Mapping of Lowly Expressed Genes
2328. Deviations from the balanced time perspective, cognitive fusion, and self-compassion in individuals with or without a depression diagnosis: different mean profiles but common links to depressive symptoms
2329. Psychological flexibility and self-compassion as predictors of well-being: Mediating role of a balanced time perspective
2330. Urbanization impacts on flooding in the Kansas River basin and evaluation of wetlands as a mitigation measure
2331. e-Spring: Circular arch mechanism for large and linear tunable stiffness control based on tuning deformation mode contributions
2332. Crushing analysis and multiobjective crashworthiness optimization of tapered square tubes under oblique impact loading
2333. Establishment and validation of nomogram models for overall survival and cancer-specific survival in spindle cell sarcoma patients
2334. A review of inter-firm relationship quality in supply chains
2335. Safe Reinforcement Learning via Probabilistic Timed Computation Tree Logic
2336. Eulerian and lagrangian statistics in the south China sea as deduced from surface drifters
2337. Coordinated voltage/var control in a hybrid AC/DC distribution network
2338. Bi-level multi time-scale voltage/var optimization and control in a hybrid distribution network
2339. Meta-PPISP: A meta web server for protein-protein interaction site prediction
2340. Deep structural research of the South China Sea: Progresses and directions
2341. Cucurbit[6]uril-Derived Sub-4 nm Pores-Dominated Hierarchical Porous Carbon for Supercapacitors: Operating Voltage Expansion and Pore Size Matching
2342. Doubly robust proximal synthetic controls
2343. SWAT-based runoff and sediment simulation in a small watershed, the loessial hilly-gullied region of China: Capabilities and challenges
2344. Experimental study on shaped steel shear connectors used in large-scale composite structures
2345. Uncovering the prominent role of satellite cells in paravertebral muscle development and aging by single-nucleus RNA sequencing
2346. Improved optoelectronic performance from the internal secondary excitation of MAPbCl3-MAPbBr3 single crystal photodetectors
2347. Rapid environmental assessment in the South China Sea: Improved inversion of sound speed profile using remote sensing data
2348. Ecological Risk Assessment of Debris Flow Disaster in Yanbian Region, China
2349. Photovoltaic Synchronous Generator: Architecture and Control Strategy for a Grid-Forming PV Energy System
2350. Rainfall-Runoff Modeling Using the SCS-CN Method and Geographic Information Systems in the Huallaga River Basin, Peru
2351. Translation and Adaptation of the Social Connectedness Scale to Portuguese
2352. Mindfulness-based cognitive therapy for young people and their carers: A mixed-method feasibility study
2353. Effect of passaging on the stemness of infrapatellar fat pad-derived stem cells and potential role of nucleostemin as a prognostic marker of impaired stemness
2354. The Limits of Populism: SBY and Jokowiâ€™s Strategies on the South China Sea Issue
2355. Walking the talk: A randomized trial exploring the role of Mindfulness Booster Sessions on skill acquisition following workshop attendance
2356. Construction and factorial validation of a short form of the Self-Compassion Scale
2357. Assessment of Spatial and Temporal Variations in Runoff Potential under Changing Climatic Scenarios in Northern Part of Karnataka in India Using Geospatial Techniques
2358. Comparison of machine learning and process-based SWAT model in simulating streamflow in the Upper Indus Basin
2359. CD26 expression on circulating CD34+/CD38- progenitor population is a specific and reliable tool for the rapid flow cytometric diagnosis of chronic myeloid leukemia-A single-center validation study
2360. Assessing the Influence of Land Cover and Climate Change Impacts on Runoff Patterns Using CA-ANN Model and CMIP6 Data
2361. Psychometric evaluation of the bangla version of the self-compassion scale-short form (scs-sf)
2362. Will self-compassion relieve distress?: A correlational study among indonesian undergraduate students
2363. Psychometric Properties of the Iranian Version of the Invalidating Childhood Environment Scale
2364. Internal validation and comparison of the prognostic performance of models based on six emergency scoring systems to predict in-hospital mortality in the emergency department
2365. An artificial neural network and SCSâ€“CN-based model for runoff estimation: a case study of the Peddavagu watershed
2366. The extracellular matrix fibulin 7 maintains epidermal stem cell heterogeneity during skin aging
2367. Improving soil moisture accounting and streamflow prediction in SWAT by incorporating a modified time-dependent Curve Number method
2368. One-step synthesis of nanostructured Ag2Mo2O7 with enhanced efficiency for supercapacitors
2369. Deriving location-specific synthetic seasonal hyetographs using GPM records and comparing with SCS curves
2370. A methodology for alleviating water scarcity of Malwa region, madhya pradesh using geospatial and geophysical techniques: Inferences from Salari watershed
2371. The Impact of Spinal Cord Stimulation on Sleep Patterns
2372. Self-compassion in overcontrolled, undercontrolled, and resilient personality types
2373. An analysis of cross-cultural equivalence of self-construal scale in Malaysia
2374. Canâ€™t we make it any shorter? The limits of personality assessment and ways to overcome them
2375. The mediating effect of mindfulness and self-compassion on leaders' communication competence and job satisfaction
2376. Self-compassion, but not compassion toward others, is associated with better physical health: A cross-sectional study
2377. Design of PV, Battery, and Supercapacitor-Based Bidirectional DC-DC Converter Using Fuzzy Logic Controller for HESS in DC Microgrid
2378. Improved rate capability and energy density of high-mass hybrid supercapacitor realized through long-term cycling stability testing and selective electrode design
2379. Is cyberbullying a stand alone construct? Using quantitative analysis to evaluate a 21st century social question
2380. The role of positive self-compassion, distress tolerance, and social problem-solving in the relationship between perfectionism and disordered eating among racially and ethnically diverse college students
2381. MicroRNA expression profile of primary prostate cancer stem cells as a source of biomarkers and therapeutic targets
2382. Construction of therapeutically relevant human prostate epithelial fate map by utilising miRNA and mRNA microarray expression data
2383. Experimental validation of solar chimney performance models and operational characteristics for small scale remote applications
2384. Finding Optimal Neuromodulation for Chronic Pain: Waves, Bursts, and beyond
2385. 'Can I please postpone my dentist appointment?' - Exploring a new area of procrastination
2386. Attachment and mental and physical health: Self-compassion and mattering as mediators
2387. The relationship among infertility, self-compassion, and well-being for women with primary or secondary infertility
2388. Spinal cord stimulation and cauda equina syndrome: Could it be a valid option? A report of two cases
2389. Spinal cord stimulation and cauda equina syndrome: Could it be a valid option? A report of two cases
2390. Topological data analysis as image steganalysis technique
2391. Effect of molecular weight on the EUV-printability of main chain scission type polymers
2392. Children's dispositional self-control - Evaluation of a parent-rating version of the SCS-K-D
2393. Dispositionelle selbstkontrollkapazitÃ¤t bei kindern: Erprobung einer fremdbeurteilerversion der SCS-K-D = Childrenâ€™s dispositional self-control: Evaluation of a parent-rating version of the SCS-K-D
2394. Comparative evaluation of pathways and gene expression profile similarity in differentiated stem cells versus normal adult cells in seven human tissues
2395. Nanobiocomposite of poly(lactide-co-glycolide)/chitosan electrospun scaffold can promote proliferation and transdifferentiation of Schwann-like cells from human adipose-derived stem cells
2396. A two-phase sequential approach to design bioenergy supply chains under uncertainty and social concerns
2397. A knowledge-based algorithm for supply chain conict detection based on OTSM-TRIZ problem flow network approach
2398. AnCaraS: A new webometrics web-spider: G-DEVS-based validation of concepts
2399. System dynamics modeling for sustainable supply chain management: A literature review and systems thinking approach
2400. Short-term effects of a multidimensional stress prevention program on quality of life, well-being and psychological resources A randomized controlled trial
2401. Longitudinal Evaluation of Language Impairment in Youth With Perinatally Acquired Human Immunodeficiency Virus (HIV) and Youth With Perinatal HIV Exposure
2402. The assessment of the spiritual competency of marriage and family therapy students: A partial replication study of the Spiritual Competency Scale
2403. Transcriptome Response of Differentiating Muscle Satellite Cells to Thermal Challenge in Commercial Turkey
2404. Comparative Analysis of CC-CV/CC Charging and Charge Redistribution in Supercapacitors
2405. The importance of body acceptance in exploring the relationships of mindfulness-based constructs to eating behaviours among gay and bisexual men
2406. Supervisory control synthesis for a waterway lock
2407. Synthesized fault-tolerant supervisory controllers, with an application to a rotating bridge
2408. Forecasting the Ambient Solar Wind with Numerical Models. I. on the Implementation of an Operational Framework
2409. Potentials of blockchain technologies for supply chain collaboration: a conceptual framework
2410. Numerical simulation and validation of impact response of axially-restrained steel-concrete-steel sandwich panels
2411. The response of axially restrained non-composite steel-concrete-steel sandwich panels due to large impact loading
2412. Nonclassical properties of the squeezing and rotating coherent state
2413. Power distribution optimization of a fully active hybrid energy storage system configuration for vehicular applications
2414. Decentralized Blockchain-Based and Trust-Aware Task Offloading Strategy for Healthcare IoT
2415. Preliminary validation of SMOS sea surface salinity measurements in the South China Sea
2416. Curiosity about people: the development of a social curiosity measure in adults
2417. Social Conduct Scale (SCS): A psychometric investigation
2418. Modeling Daily Streamflow from Idamalayar Catchment Using SWAT
2419. Spinal cord stimulation (SCS) in conjunction with peripheral nerve field stimulation (PNfS) for the treatment of complex pain in failed back surgery syndrome (FBSS)
2420. Development of a conceptual model for lean supply chain planning in industry 4.0: multidimensional analysis for operations management
2421. S66: A Well-balanced Database of Benchmark Interaction Energies Relevant to Biomolecular Structures
2422. Evaluation of the time of concentration estimation methods for small rural watersheds
2423. Propiedades psicomÃ©tricas de la escala de autocompasiÃ³n en estudiantes de medicina de lima
2424. Researched topics, patterns, barriers and enablers of artificial intelligence implementation in supply chain: a Latent-Dirichlet-allocation-based topic-modelling and expert validation
2425. Parenting stress, social support, self-compassion, and parenting practices among mothers of children with ASD and ADHD
2426. Deois flexuosa (Hemiptera: Cercopidae) in Perennial Forage Species (Poaceae): Quali-quantitative Impacts and Tolerance Expression
2427. Determination of Ethyl Carbamate in Sugar Cane Spirit by Direct Injection Electrospray Ionization Tandem Mass Spectrometry Using 18-Crown-6/Trifluoroacetic Acid Spiking Additives
2428. Bone marrow-derived fibroblast growth factor-2 induces glial cell proliferation in the regenerating peripheral nervous system
2429. Aberrant salience in cannabis-induced psychosis: a comparative study
2430. Therapeutic drug monitoring-guided continuous infusion of piperacillin/tazobactam significantly improves pharmacokinetic target attainment in critically ill patients: a retrospective analysis of four years of clinical experience
2431. Development of a multidimensional, multi-informant measure of teacher mindfulness as experienced and expressed in the middle school classroom
2432. Flexible truck modeling and investigation of coupling between rigid and flexible dynamics
2433. Mastitis detection from milk mid-infrared (MIR) spectroscopy in dairy cows
2434. Use of differential somatic cell count, somatic cell score, and milk mid-infrared spectral analysis for monitoring mastitis in dairy cows during routine milk recording
2435. Prediction of Acute and Chronic Mastitis in Dairy Cows Based on Somatic Cell Score and Mid-Infrared Spectroscopy of Milk
2436. Impact of demographic factors on recognition of persons with depression and anxiety in primary care in Slovenia
2437. Back pain: a real target for spinal cord stimulation?
2438. Combining Awake Anesthesia with Minimal Invasive Surgery Optimizes Intraoperative Surgical Spinal Cord Stimulation Lead Placement
2439. Should we Oppose or Combine Waveforms for Spinal Cord Stimulation in PSPS-T2 Patients? A Prospective Randomized Crossover Trial (MULTIWAVE Study)
2440. Stress-induced cognition among radiologic technologists in COVID-19 quarantine centres in Palestine
2441. A comparison between counselors who practice meditation and those who do not on compassion fatigue, compassion satisfaction, burnout and self-compassion
2442. Return of the cold: How hypothermic oxygenated machine perfusion is changing liver transplantation
2443. A program evaluation of the institute for meditation and psychotherapy's certificate program
2444. Excited states using the simplified Tamm-Dancoff-Approach for range-separated hybrid density functionals: development and application
2445. Thermo-mechanical aspects of adiabatic shear failure of AM50 and Ti6Al4V alloys
2446. Thermo-mechanical aspects of adiabatic shear failure of AM50 and Ti6A14V alloys
2447. Calibration and Validation of CN Values for Watershed Hydrological Response
2448. Measurement of succinyl-carnitine and methylmalonyl-carnitine on dried blood spot by liquid chromatography-tandem mass spectrometry
2449. Power flow control strategy for electric vehicles with renewable energy sources
2450. The Spiritual Competency Scale: A comparison to the aservic spiritual competencies
2451. DNA Methylation Analysis Reveals Distinct Patterns in Satellite Cellâ€“Derived Myogenic Progenitor Cells of Subjects with Spastic Cerebral Palsy
2452. Technical note: Evaluation of a commercial on-farm milk leukocyte differential tester to identify subclinical mastitis cases in dairy cows
2453. Does mindfulness change the mind? A novel psychonectome perspective based on Network Analysis
2454. The impact of compassion meditation training on psychological variables: A network perspective
2455. Single-Cell RNA Sequencing of Lymph Node Stromal Cells Reveals Niche-Associated Heterogeneity
2456. Shortâ€term and longâ€term effects of training in EFT: A multinational study in Spanishâ€speaking countries
2457. Mindfulness Integrative Model (MIM): Cultivating positive states of mind towards oneself and the others through mindfulness and self-compassion
2458. Rapid effects of inhaled corticosteroids in acute asthma: an evidence-based evaluation
2459. Muscle Satellite Cell Heterogeneity: Does Embryonic Origin Matter?
2460. A systematic review of the roles of body image flexibility as correlate, moderator, mediator, and in intervention science (2011â€“2018)
2461. Differential disclosure of suicidal intent to clinicians versus researchers: Associations with concurrent suicide crisis syndrome and prospective suicidal ideation and attempts
2462. An overview and comparison of two proposed suicide-specific diagnoses: Acute suicidal affective disturbance and suicide crisis syndrome
2463. Cross-national presence and sociodemographic correlates of the suicide crisis syndrome
2464. Organizational culture and supply chain strategy: A framework for effective information flows
2465. Disentangling components of flexibility via the hexaflex model: Development and validation of the Multidimensional Psychological Flexibility Inventory (MPFI)
2466. Construct, measurement and assessment of social competence in early adolescence
2467. Curve number values for olive orchards under different soil management
2468. Assessment of coal supply chain under carbon trade policy by extended exergy accounting method
2469. Assessment of coal supply chain under carbon trade policy by extended exergy accounting method
2470. The Social Self-Compassion Scale (SSCS): Development, validity, and associations with indices of well-being, distress, and social anxiety
2471. Thermoreversi ble hyaluronan-hydrogel and autologous nucleus pulposus cell deli very regenerates human i ntervertebral di scs i n an ex vi vo, physi ologi cal organ culture model
2472. Flexural performance of steel-concrete-steel sandwich beams with lightweight fiber-reinforced concrete and corrugated-strip connectors: Experimental tests and numerical modeling
2473. Unbundling subjective career success: a sequential mediation analysis
2474. A Prospective Study of the Intra- and Postoperative Efficacy of Intraoperative Neuromonitoring in Spinal Cord Stimulation
2475. Universal worth: Construct and scale development
2476. Evaluating health worker performance in Benin using the simulated client method with real children
2477. Redesigning traditional linear supply chains into circular supply chainsâ€“A study into its challenges
2478. Sustainability performance approach in Malaysia's SMEs for Improving Green Supply Chain Management (GSCM); An Application of Quality Function Deployment (QFD)
2479. Differential predictability of four dimensions of affect intensity
2480. Adaptive spectroscopic visible-light optical coherence tomography for clinical retinal oximetry
2481. Putative stem cells in mucosas of the esophago-gastrointestinal tract
2482. A brief scale to measure caring for bliss: Conceptualization, initial development, and validation
2483. The Brief Suicide Cognitions Scale: Development and Clinical Application
2484. A Wireless EV Charging Topology with Integrated Energy Storage
2485. A wireless power transfer system based on a modified full bridge for dynamic EV charging
2486. Ceremonial Ayahuasca in Amazonian Retreats-Mental Health and Epigenetic Outcomes From a Six-Month Naturalistic Study
2487. Corrigendum: Ceremonial ayahuasca in amazonian retreatsâ€”Mental health and epigenetic outcomes from a six-month naturalistic study
2488. Single-cell stabilization method identifies gonadotrope transcriptional dynamics and pituitary cell type heterogeneity
2489. Assessing collaborative interactions in a teacher professional community: The validity and reliability of a survey instrument (Connecticut)
2490. The conspiracy of silence in families of the terminal oncological patients
2491. A proposed model to account human factors in safety-critical Systems
2492. A COMPARISON BETWEEN CIVILIAN AND MILITARY PHYSICAL THERAPISTS' KNOWLEDGE IN MANAGING MUSCULOSKELETAL CONDITIONS: A DESCRIPTIVE STUDY
2493. A Novel Composite Metric for Predicting Patient Satisfaction With Spinal Cord Stimulation
2494. Leveraging multi-sensor hydrologic data for model validation
2495. â€˜I want to scream it from the rooftops now [â€¦] there are other pathwaysâ€™: What young people learned from a co-designed post-secondary transition programme
2496. Surgical Cholecystectomy Score (SCS) for grading the difficulty of laparoscopic cholecystectomy
2497. Validation and psychometric characteristics of the hungarian version of the contingent self-esteem scale
2498. Relationship between self-compassion, self-esteem and mental health
2499. A feltÃ©teles Ã¶nbecsÃ¼lÃ©s skÃ¡la (CSES-H) Magyar vÃ¡ltozatÃ¡nak validÃ¡lÃ¡sa Ã©s pszichometriai jellemzÅ‘i = Validation and psychometric characteristics of the Hungarian version of the Contingent Self-Esteem Scale
2500. Moisture Sorption in Moulded Fibre Trays and Effect on Static Compression Strength
2501. Applying data-flow analysis to models a novel approach for model analysis
2502. Conjunctive Use Modeling Using SWAT and GMS for Sustainable Irrigation in Khatav, India
2503. An investigation of the associations between contingent self-worth and aspirations among Iranian university students
2504. Experimental investigation and theoretical modeling of textured silicon solar cells with rear metallization
2505. A mindfulness and compassion-based program applied to pregnant women and their partners to decrease depression symptoms during pregnancy and postpartum: Study protocol for a randomized controlled trial
2506. A bias of self-reports among repressors: Examining the evidence for the validity of self-relevant and health-relevant personal reports
2507. Psychometric properties of the Persian spiritual coping strategies scale in hemodialysis patients
2508. Polyoxometalate-Induced Nano-Engineered Composite Materials for Energy Storage Supercapacitor Applications
2509. Finite Element Modeling of Steelâ€“Concreteâ€“Steel Sandwich Beams with Bolt Connectors Under Drop Weight Impact
2510. Generalized Self-discharge Model for a Series of Supercapacitors
2511. Simulation of runoff in baitarani basin using composite and distributed curve number approaches in HEC-HMS model
2512. A proposal for a comprehensive risk scoring system for predicting postoperative complications in octogenarian patients with medically operable lung cancer: JACS1303
2513. Flood Prediction for the Bang Pakong River by RUBICON Mathematical Model
2514. Mesenchymal stem cell response to growth factor treatment and low oxygen tension in 3-dimensional construct environment
2515. Analyzing supply chain uncertainty to deliver sustainable operational performance: Symmetrical and asymmetrical modeling approaches
2516. Thoracic spinal cord neuromodulation obtunds dorsal root ganglion afferent neuronal transduction of the ischemic ventricle
2517. Self-emulsifying drug delivery systems containing sulfate-based surfactants: Are they responsive to alkaline phosphatase?
2518. Comparative electrochemical properties of MO (ZrO2, V2O5) based polyaniline nanocomposites for high-performance supercapacitor applications
2519. Runoff Estimation for the Central Region of the Lesser Zab River Watershed Using the SCS-Curve Number Method and GIS
2520. Development and Testing of the Psychodiagnostic Method "Scale of Coping with Infertility"
2521. High-frequency spinal cord stimulation at 10 kHz for widespread pain: a retrospective survey of outcomes from combined cervical and thoracic electrode placements
2522. Initial validation of the Brazilian Mindful Eating Scale among overweight and low-income women
2523. Self-compassion as a moderator of the relationship between rumination, self-reflection and stress
2524. Predicting the relationships between virtual enterprises and agility in supply chains
2525. A structural equation model for predicting virtual enterprise and agile supply chain relation
2526. Integration of fuel cells and supercapacitors in electrical microgrids: Analysis, modelling and experimental validation
2527. Targeting the Bacillus subtilis genome: an efficient and clean method for gene disruption
2528. Coupling of classical and renewable energy sources: Modeling and power flow management
2529. Medical Device Related Pressure Injury in the Treatment of Chronic Pain: An Early Sign of Explantation in Suspected Infection
2530. Switched-Capacitor-Based Quadruple-Boost Nine-Level Inverter
2531. Mimo_ofdm efficient channel estimation systems in mobility sensing
2532. Cultivating emotional balance in professional caregivers: A pilot intervention
2533. The measurement of compassionate leadership: Adaptation and Spanish validation of the Compassionate Leadership Selfâ€Reported Scale
2534. Attachment-Based Compassion Therapy for Reducing Anxiety and Depression in Fibromyalgia
2535. EstÃ¡gios de mudanÃ§a: CorrelaÃ§Ã£o entre duas formas de avaliaÃ§Ã£o = Stages of change: Correlation between two forms of evaluation
2536. Study and application of photo-modified cassava starch with lactic acid and UV-C irradiation
2537. Integrating parallel DEVS and equation-based object-oriented modeling
2538. Assessment and evaluation of groundwater vulnerability index maps of Upper Palar River Basin, Tamilnadu, India
2539. Flood Inundation Modeling Using Nakagami-m Distribution Based GIUH for a Partially Gauged Catchment
2540. Development of a Secondary Crash Identification Algorithm and occurrence pattern determination in large scale multi-facility transportation network
2541. Assessment of Soil Quality based on Relationship of Soil Carbon Stocks with Different Soil Parameters in Lakkampura Mini-watershed in Davanagere under Semi-arid Alfisols in India
2542. A new representation learning based maximum power operation towards improved energy management integration with DG controllers for photovoltaic generators using online deep exponentially expanded RVFLN algorithm
2543. Design and Validation of an Automated Process for the Expansion of Peripheral Blood-Derived CD34+ Cells for Clinical Use After Myocardial Infarction
2544. The complementing role of sustainability standards in managing international and multi-tiered mineral supply chains
2545. Sustainable supply chain management for minerals
2546. A study on blockchain-based marketplace governance platform adoption: a multi-industry perspective
2547. Validity and reliability of the smartphone craving scale: Modifying and adaptation of the penn alcohol craving scale to the Turkish culture as smartphone craving scale
2548. AkÄ±llÄ± Telefon AÅŸerme Ã–lÃ§eÄŸinin geÃ§erliliÄŸi ve gÃ¼venilirliÄŸi: Penn Alkol AÅŸerme Ã–lÃ§eÄŸinin TÃ¼rk kÃ¼ltÃ¼rÃ¼ne AkÄ±llÄ± Telefon AÅŸerme Ã–lÃ§eÄŸi olarak deÄŸiÅŸtirilmesi ve uyarlamasÄ± = Validity and reliability of the Smartphone Craving Scale: Modifying and adaptation of the Penn Alcohol Craving Scale to the Turkish culture as Smartphone Craving Scale
2549. Nurses in the triage of the emergency department: self-compassion and empathy
2550. Deficits in domains of social cognition in schizophrenia: a meta-analysis of the empirical evidence
2551. Executive functions in schizophrenia: Defining and refining the constructs
2552. Reconstructing Neural Activity and Kinematics Using a Systems-Level Model of Sensorimotor Control
2553. Reconstructing Neural Activity and Kinematics Using a Systems-Level Model of Sensorimotor Control
2554. Performance Limitations in Sensorimotor Control: Trade-Offs Between Neural Computation and Accuracy in Tracking Fast Movements
2555. Retrospective Analysis of Real-World Outcomes of 10 kHz SCS in Patients with Upper Limb and Neck Pain
2556. Simultaneous Distribution Network Reconfiguration and Optimal Allocation of Renewable-Based Distributed Generators and Shunt Capacitors under Uncertain Conditions
2557. Sexual Compulsivity Scale, Compulsive Sexual Behavior Inventory, and Hypersexual Disorder Screening Inventory: Translation, Adaptation, and Validation for Use in Brazil
2558. Body image-related cognitive fusion and disordered eating: The role of self-compassion and sad mood
2559. Development and preliminary validation of the Asthma Intensity Manifestations Score (AIMS) derived from Asthma Control Test, FEV(1), fractional exhaled nitric oxide, and step therapy assessments
2560. Solving constraint systems from traffic scenarios for the validation of autonomous driving
2561. Character tests in dog breed clubs in Switzerland. Results of a questionnaire survey
2562. Sustainable value creation through information technology-enabled supply chains in emerging markets
2563. Mobile financial service-enabled micro-businesses driving sustainable value creation in emerging markets
2564. Intraoperative Neurophysiological Monitoring During Spinal Cord Stimulation Surgery: A Systematic Review
2565. Functional characterization of quiescent keratinocyte stem cells and their progeny reveals a hierarchical organization in human skin epidermis
2566. Psychometric- and quality-of-life assessment in long-term gliblastoma survivors
2567. Psychometric- and quality-of-life assessment in long-term glioblastoma survivors
2568. Development of a mapping framework for ecosystem services: The case of sediment control supply at a watershed scale in Newfoundland, Canada
2569. Power Distribution Systems Optimal Outage Restoration with Miscoordination Detection
2570. Incorporating variable source area hydrology into a curve-number-based watershed model
2571. Self-compassion and intuitive eating in college women: Examining the contributions of distress tolerance and body image acceptance and action
2572. GATA4 Regulates Blood-Testis Barrier Function and Lactate Metabolism in Mouse Sertoli Cells
2573. Private and public self in context of age, gender and five-factor model of personality
2574. SÃºkromnÃ© a verejne self v kontextÃ© veku, rodu a pÃ¤ftfaktorovÃ©ho modelu osobnosti = Private and public self in context of age, gender and five-factor model of personality
2575. Electrochemical profiling and liquid chromatography-mass spectrometry characterization of synthetic cathinones: From methodology to detection in forensic samples
2576. The Self-Invalidation Due to Emotion Scale: Development and psychometric properties
2577. The senior concerns survey: An exploratory factor analysis with college seniors and nontraditional students
2578. Self-identities and BMI of Minnesotan soy consumers and non-consumers
2579. Time-Dependent Double-Hybrid Density Functionals with Spin-Component and Spin-Opposite Scaling
2580. Assessment of flash floods in a small Mediterranean catchment using terrain analysis and remotely sensed data: A case study in the Torrente Teiro, Liguria, Italy
2581. Investigation of Schwann cell behaviour on RGD-functionalised bioabsorbable nanocomposite for peripheral nerve regeneration
2582. Artificial neural network-based secured communication strategy for vehicular ad hoc network
2583. Relations among self-compassion, PTSD symptoms, and psychological health in a trauma-exposed sample
2584. The association between communication behavior and psychological distress among couples coping with cancer: Actor-partner effects of disclosure and concealment
2585. Assessment of the effectiveness of green practices in the management of two supply chains
2586. Self-compassion, health behaviors, self-regulation, and affective states among individuals at risk of or diagnosed with a chronic disease: A scoping review
2587. Catchy catchments: Estimating outflow of the Iponan watershed using Anylogic
2588. A mediation model for ideas of reference: The role of the gray model, self-consciousness, and emotional symptoms
2589. Optimal Design of Supercapacitor Stacks for Size-critical Applications
2590. Publicly verifiable secure cloud storage for dynamic data using secure network coding
2591. A Mediation Model for Ideas of Reference
2592. Supply chain risk management to achieve healthcare supply chain operational excellence: a fsQCA and PLS-SEM approach
2593. The role of self-compassion and emotional approach coping in the relationship between maladaptive perfectionism and psychological distress among East Asian international students
2594. How women varsity athletes high in self-compassion experience unexpected stressors
2595. How women varsity athletes high in self-compassion experience unexpected stressors
2596. A Novel Low-Profile Deployable and Retrievable Epidural Lead Array System
2597. POLCA system for supply chain management: simulation in the automotive industry
2598. How Self-Efficacy toward, Perceived Importance of, and Beliefs about Smoking Cessation Support Impact-Related Behaviors in Japanese Nursing Professionals
2599. Long-term follow-up of patients with cardiac syndrome X treated by spinal cord stimulation
2600. A breakthrough in circular economy: Using a closed-loop framework to assess the circularity of supply chains
2601. The impact of the national standardization system on ranking the supply chain stages improvement
2602. Academic resilience, moral perfectionism, and self-compassion among undergraduate nursing students: A cross-sectional, multi-center study
2603. A tuned gelatin methacryloyl (GelMA) hydrogel facilitates myelination of dorsal root ganglia neurons in vitro
2604. Empowering Supply chains Resilience: LLMs-Powered BN for Proactive Supply Chain Risk Identification
2605. Optimization of virtual closed-loop supply chain under uncertainty: application of IoT
2606. Evaluation of QoS-compliant overlays under Denial of Service Attacks
2607. Mechanosensitive channel of large conductance enhances the mechanical stretching-induced upregulation of glycolysis and oxidative metabolism in Schwann cells
2608. Nano-ZnO impairs anti-predation capacity of marine mussels under seawater acidification
2609. A Mesh-Structured Switched-Capacitor Equalizer for Lithium-Ion Battery Strings of Electric Vehicles
2610. Perceived clinician competence to work with spiritual issues in supervision
2611. Evidence-based consensus guidelines on patient selection and trial stimulation for spinal cord stimulation therapy for chronic non-cancer pain
2612. Statistical Prediction of the South China Sea Surface Height Anomaly
2613. Binary nanosheet frameworks of graphene/polyaniline composite for high-areal flexible supercapacitors
2614. Multifunctional power unit by hybridizing contact-separate triboelectric nanogenerator, electromagnetic generator and solar cell for harvesting blue energy
2615. Security of self-certified signatures
2616. Assessment of spatio-temporal variation of water balance components by simulating the hydrological processes of a large complex watershed
2617. Investigating an empirical approach to predict sediment yield for a design storm: a multi-site multi-variable study
2618. A modified NRCS-CN method for eliminating abrupt runoff changes induced by the categorical antecedent moisture conditions
2619. Can smart supply chain bring agility and resilience for enhanced sustainable business performance?
2620. Diagnostic accuracy of pan-TRK immunohistochemistry in differentiating secretory carcinoma from acinic cell carcinoma of salivary gland-A systematic review
2621. Effect of local field correction in screening dependence of superconducting state parameters for Be90Al10 and Be 70Al30 metallic glasses
2622. Fuzzy logic-based robot navigation in static environment with dead cycle obstacles
2623. Detecting Regional Fibrosis in Hypertrophic Cardiomyopathy: The Utility of Myocardial Strain Based on Cardiac Magnetic Resonance
2624. The failure mechanisms of micro-scale cantilevers in shock and vibration stimuli
2625. Correlations Between Family History of Psychiatric Illnesses and Outcomes of Spinal Cord Stimulation
2626. Novel formula design of alkali-activated slag solidified construction spoil considering flowability and compressive strength
2627. A proteome map of primary cultured rat Schwann cells
2628. A proteomic view on the differential phenotype of Schwann cells derived from mouse sensory and motor nerves
2629. A Time-Bound and Hierarchical Key Management Scheme for Secure Multicast Systems
2630. Current Reconstruction of Three-Phase Voltage Source Inverters Considering Current Ripple
2631. Negative school gossip and prosocial behavior among high school students: Mediated by basic psychological needs satisfaction and moderated by self-compassion
2632. Heat Shock Proteins and their Protective Roles in Stem Cell Biology
2633. Estimation of mode I static fracture toughness of carbonate rock using small rock fragments
2634. Imaging of the Upper Mantle Beneath Southeast Asia: Constrained by teleseismic P-wave tomography
2635. Downlink analysis of hetnets with millimeter wave small cells
2636. An improved MUSLE model incorporating the estimated runoff and peak discharge predicted sediment yield at the watershed scale on the Chinese Loess Plateau
2637. Storm-based CSLE that incorporates the estimated runoff for soil loss prediction on the Chinese Loess Plateau
2638. A Modified SCS-CN Method Incorporating Storm Duration and Antecedent Soil Moisture Estimation for Runoff Prediction
2639. An improved method that incorporates the estimated runoff for peak discharge prediction on the Chinese Loess Plateau
2640. An improved SCS-CN method incorporating slope, soil moisture, and storm duration factors for runoff prediction
2641. Improved SMA-based SCS-CN method incorporating storm duration for runoff prediction on the Loess Plateau, China
2642. Revised runoff curve number for runoff prediction in the Loess Plateau of China
2643. Modelling hydrology and water quality processes in the Pengxi River basin of the Three Gorges Reservoir using the soil and water assessment tool
2644. Construction of binder-free 3-D porous NiMn LDH electrode materials for high performance asymmetric supercapacitors using dynamic hydrogen bubbles as template
2645. Simultaneous screening of 239 synthetic cannabinoids and metabolites in blood and urine samples using liquid chromatography-high resolution mass spectrometry
2646. Asymmetric supercapacitors with high energy densities
2647. Exploring the relationship between mental well-being, exercise routines, and the intake of image and performance enhancing drugs during the coronavirus disease 2019 pandemic: A comparison across sport disciplines
2648. Associations among dispositional mindfulness, self-compassion, and executive function proficiency in early adolescents
2649. Investigation of cathinone analogs targeting human dopamine transporter using molecular modeling
2650. Mapping of the Spinal Sensorimotor Network by Transvertebral and Transcutaneous Spinal Cord Stimulation
2651. Augmenting the technology acceptance model with trust model for the initial adoption of a blockchain-based system
2652. Structure and validity of the Clinical Perfectionism Questionnaire in female adolescents
2653. Scale-factor analysis of a geometrically compensated (100) single-crystal silicon vibratory ring gyroscope
2654. Meridional overturning circulation in the South China Sea envisioned from the high-resolution global reanalysis data GLBa0.08
2655. Untangling the complexity generating material delivery â€œschedule instabilityâ€: insights from automotive OEMs
2656. Development and testing of high concentration flat-plate Fresnel lenses
2657. Advancement in Supercapacitors for IoT Applications by Using Machine Learning: Current Trends and Future Technology
2658. Mindfulness for children with ADHD and Mindful Parenting (MindChamp): Protocol of a randomised controlled trial comparing a family Mindfulness-Based Intervention as an add-on to care-as-usual with care-as-usual only
2659. Psychometric properties and validation of the Phenomenological Body Shame Scaleâ€”Revised (PBSS-R)
2660. Multi-level nondestructive analysis of joint-debond effects in sandwich composite structure
2661. GWAS and gene networks for milk-related traits from test-day multiple lactations in Portuguese Holstein cattle
2662. Boosting Empathy and Compassion Through Mindfulness-Based and Socioemotional Dyadic Practice: Randomized Controlled Trial With App-Delivered Trainings
2663. Single haplotype admixture models using large scale HLA genotype frequencies to reproduce human admixture
2664. Symptom Clusters in Patients With Advanced Cancer: A Prospective Longitudinal Cohort Study to Examine Their Stability and Prognostic Significance
2665. Tools for strengths-based assessment and evaluation
2666. The relationship of hygiene, motivator, and professional strategic capabilities to the performance of Australian music festival event management organizations
2667. Epidural unilateral stimulation with "adaptive stim" option in treatment of type II CRPS
2668. The Incidence and Management of Postdural Puncture Headache in Patients Undergoing Percutaneous Lead Placement for Spinal Cord Stimulation
2669. Can Self-Compassion Promote Healthcare Provider Well-Being and Compassionate Care to Others? Results of a Systematic Review
2670. Reversible Thermochromism in All-Inorganic Lead-Free Cs3Sb2I9 Perovskite Single Crystals
2671. Phonon-Assisted Reversible Thermochromism in a Lead-Free Antimony-Based Cs3Sb2Br9Perovskite
2672. Multiport Multilevel Inverter for High-Frequency AC Applications
2673. A Novel Common-Ground Multisource Inverter
2674. A 13-Level Switched-Capacitor-Based Common-Ground Boosting Inverter
2675. 3D printing of scaffold for cells delivery: Advances in skin tissue engineering
2676. Expansion of Empirical-Statistical Based Topographic Correction Algorithm for Reflectance Modeling on Himalayan Terrain using AWiFS and MODIS Sensor
2677. Energy management and control for direct current microgrid with composite energy storage system using combined cuckoo search algorithm and neural network
2678. Variable structure control for dynamic power-sharing and voltage regulation of DC microgrid with a hybrid energy storage system
2679. Small-signal modeling and stability analysis of autonomous direct current microgrid with distributed energy storage system
2680. Improved encryption and obfuscation process of lightweight secured auditable cloud storage with data dynamics
2681. Test Norms and a Psychometric Examination of the German Adaption of the Brief Version of the Self-Control Scale (SCS-K-D)
2682. Normierung und testtheoretische ÃœberprÃ¼fung der deutschen Adaptation der Kurzform der Self-Control Scale (SCS-K-D) = Test norms and a psychometric examination of the German adaption of the Brief version of the Self-Control Scale (SCS-K-D)
2683. Robustness of topological superconductivity in proximity-coupled topological insulator nanoribbons
2684. Hydrological modelling of Wadi ressoul watershed, Algeria, by HEC-HMS model
2685. An examination of the factor structure of the Effects of Meditation scale
2686. A simple and robust serum-free media for the proliferation of muscle cells
2687. Supercapacitor Modeling: A System Identification Approach
2688. Validation of the pediatric stroke outcome measure for classifying overall neurological deficit
2689. Self-consciousness and similar personality constructs
2690. Development and validation of a measure of self-critical rumination
2691. Successful use of high-frequency spinal cord stimulation following traditional treatment failure
2692. Deriving biomedical diagnostics from NMR spectroscopic data
2693. Development and assessment of rules to parameterise the ACRU model for design flood estimation
2694. Experimental spinal cord stimulation and neuropathic pain: mechanism of action, technical aspects, and effectiveness
2695. Informing measurement of gender differences in suicide risk and resilience: A national study of United States military veterans
2696. Preliminary validation of the Cognitive Affective Mindfulness Scaleâ€“Revised in cancer populations
2697. Psychometric Properties of Social Connectedness Scale
2698. A fuzzy maturity-based method for lean supply chain management assessment
2699. Biomechanical comparison of different volar fracture fixation plates for distal radius fractures
2700. SCS+C: A modified sun-canopy-sensor topographic correction in forested terrain
2701. Topographic correction in forested terrain using sun-canopy-sensor geometry and canopy reflectance models
2702. Estimating aboveground forest biomass from canopy reflectance model inversion in mountainous terrain
2703. Evaluation of Kostiakov's infiltration equation in furrow irrigation design according to FAO method
2704. Effects of prefrontal 40 hz-centered EEG band neurofeedback on emotional state and cognitive functions in adolescents
2705. A formal approach to protocol offload for web servers applied to a TCP offload engine and web traffic
2706. Estimation of peak discharge and flood volume in ungauged basins using HydroCAD software
2707. Sampling Design and Spatial Modeling of Available Phosphorus in a Complex Agricultural Area in Southern Brazil
2708. Physician exhaustion and work engagement during the COVID-19 pandemic: A longitudinal survey into the role of resources and support interventions
2709. The Sexual Communication Scale: a measure of frequency of sexual communication between parents and adolescents
2710. Investigations into the stability of tethered palladium(II) pincer complexes during heck catalysis
2711. Development and validation of the forms of Self-Criticizing/Attacking and Self-Reassuring Scaleâ€”Short Form
2712. Development and psychometric testing of the self-care scale for patients with chronic atrial fibrillation (AF-SCS-10)
2713. Innovative lightweight deep learning architecture for enhanced rice pest identification
2714. On the Spatial and Temporal Variations of Primary Production in the South China Sea
2715. Time Series Analysis-Based Long-Term Onboard Radiometric Calibration Coefficient Correction and Validation for the HY-1C Satellite Calibration Spectrometer
2716. Overvoltage Protection Performance of Series Compensation in Transmission System
2717. Parameter Identification and Maximum Power Estimation of Battery/Supercapacitor Hybrid Energy Storage System Based on Cramer-Rao Bound Analysis
2718. The battery-supercapacitor hybrid energy storage system in electric vehicle applications: A case study
2719. Construction of a High Spatiotemporal Resolution Dataset of Satellite-Derived pCO2and Air-Sea CO2Flux in the South China Sea (2003-2019)
2720. Evaluating reliability and validity of lean, agile and leagile supply chain constructs in Indian manufacturing industry
2721. Failure analysis of MMC single lap bolted joint
2722. CAD/CAM cobalt-chromium alloy single crowns in posterior regions: 4-year prospective clinical study
2723. The relationship between acceptance, self-compassion and hope in infertile women: a structural equation analysis
2724. Investigation of the direct runoff generation mechanism for the analysis of the SCS-CN method applicability to a partial area experimental watershed
2725. Measuring compassion toward others: Dimensionality of the compassion scale in community adolescents and in adolescents with behavioral disorders
2726. Discrepancy Between Reported and Calculated Pain Reduction in Patients With Spinal Cord Stimulation Therapy and Lack of Agreement Between Patient Satisfaction and Degree of Pain Relief
2727. Characterization of the prohormone complement in cattle using genomic libraries and cleavage prediction approaches
2728. Development, characterization and optimization of a new bone cement based on calciumâ€“strontium aluminates and chitosan-glycerin solution
2729. Mid-infrared prediction of lactoferrin content in bovine milk: Potential indicator of mastitis
2730. Psychometric properties of the German version of the suicide cognitions scale in two clinical samples
2731. Supervised construct scoring to reduce personality assessment length: A field study and introduction to the Short 10
2732. Prosthetic protocols in implant-based oral rehabilitations: A systematic review on the clinical outcome of monolithic all-ceramic single- and multi-unit prostheses
2733. Clinical outcomes of all-ceramic single crowns and fixed dental prostheses supported by ceramic implants: A systematic review and meta-analyses
2734. Composite material based on an ablative phenolic resin and carbon fibers
2735. Does the Superior Colliculus Control Perceptual Sensitivity or Choice Bias during Attention? Evidence from a Multialternative Decision Framework
2736. Investigating impact of land-use and land cover changes on hydro-ecological balance using GIS: insights from IIT Bombay, India
2737. A New Nine-Level Highly Efficient Boost Inverter for Transformerless Grid-Connected PV Application
2738. Testing of coupled SCS curve number model for estimating runoff and sediment yield for eleven watersheds
2739. Liquid chromatography-tandem mass spectrometry screening method using information-dependent acquisition of enhanced product ion mass spectra for synthetic cannabinoids including metabolites in urine
2740. Can athletes be tough yet compassionate to themselves? Practical implications for NCAA mental health best practice no 4
2741. Emotional freedom techniques in the treatment of unhealthy eating behaviors and related psychological constructs in adolescents: A randomized controlled pilot trial
2742. Assessing moral injury and its clinical associations in a UK secure care population
2743. Becoming self-compassionate step by step â€” A field study on the effect of long-distance walking on self-compassion in hikers traveling the Camino FrancÃ©s
2744. Correction to: Becoming selfâ€‘compassionate step by stepâ€”A field study on the effect of longâ€‘distance walking on selfâ€‘compassion in hikers traveling the Camino FrancÃ©s
2745. Sarm1-Dependent Metabolic Reprogramming of Schwann Cells Following Nerve Injury
2746. The brain that longs to care for itself: The current neuroscience of self-compassion
2747. In search of clarity: self-esteem and domains of confidence and confusion
2748. Interleukin 8 haplotypes drive divergent responses in uterine endometrial cells and are associated with somatic cell score in Holstein-Friesian cattle
2749. A prospective examination of self-compassion as a predictor of depressive symptoms in children and adolescents
2750. A prospective examination of self-compassion as a predictor of depressive symptoms in children and adolescents
2751. External validation of the simple clinical score and the HOTEL score, two scores for predicting short-term mortality after admission to an acute medical unit
2752. A Comparative Assessment of Different Loss Methods Available Iin Mike Hydro River-uhm
2753. Call admission control in wireless networks: Probabilistic approach and efficiency evaluation
2754. Probabilistic call admission control in wireless multiservice networks
2755. A probabilistic approach for fair-efficient call admission control in wireless multiservice networks
2756. Towards a New Qualitative Screening Assay for Synthetic Cannabinoids Using Metabolomics and Machine Learning
2757. Measuring the effect of an compassionate intervention on a sample with anxiety symptoms
2758. The role of self-consciousness in trait forgiveness
2759. Detecting surface Kuroshio front in the Luzon Strait from multichannel satellite data using neural networks
2760. Levels, profiles and potential human health risks of brominated and parent polycyclic aromatic hydrocarbons in soils around three different types of industrial areas in China
2761. Engineered Schwann Cell-Based Therapies for Injury Peripheral Nerve Reconstruction
2762. Interoceptive Awareness, Decision-Making and Impulsiveness in Male Patients with Alcohol or Opioid Use Disorder
2763. Characteristics of Human Natal Stem Cells Cultured in Allogeneic Medium
2764. Quantitative proteomics of protein complexes and their implications for cell reprograming and pluripotency
2765. The 10-Item Version Singelisâ€™s Self-Construal Scale (SCS-Short Version) for Korean Adults: Psychometric Properties, Measurement Invariance with European Americans, and Associations with Social Anxiety
2766. Orchestrating product provenance story: When IOTA ecosystem meets electronics supply chain space
2767. Unequal burden of equal risk factors of diabetes between different gender in India: a cross-sectional analysis
2768. Csr maturity model for smart city assessment
2769. Health-related quality of life associated with systemic corticosteroids
2770. Spatiotemporal microRNA profile in peripheral nerve regeneration: MiR-138 targets vimentin and inhibits Schwann cell migration and proliferation
2771. Blockchain technology adoption inÂ Halal traceability scheme ofÂ theÂ food supply chain: evidenceÂ from Indonesian firms
2772. Psychological assessment of spinal cord stimulator candidates: Predicting long-term post-surgical pain
2773. Seasonal variability of the wind-generated near-inertial energy flux in the South China Sea
2774. Synergetics: The cooperative phenomenon in multi-compressions S-CO2 power cycles
2775. Estimating four-dimensional internal wave spectrum in the northern South China Sea
2776. Numerical simulations of typhoon hagupit (2008) using WRF
2777. Graphene oxide assisted triple network hydrogel electrolyte with high mechanical and temperature stability for self-healing supercapacitor
2778. Molecularly engineered metal-based bioactive soft materials - Neuroactive magnesium ion/polymer hybrids
2779. A Holistic Review of C = C Crosslinkable Conjugated Molecules in Solution-Processed Organic Electronics: Insights into Stability, Processibility, and Mechanical Properties
2780. A DC Bus Oscillation Suppression Strategy Based on Series Voltage Compensator for Diode Rectifier
2781. Analysis of Wave Fields under Tropical Cyclones in the South China Sea
2782. Short-term spinal cord stimulation is an effective therapeutic approach for herpetic-related neuralgia-A Chinese nationwide expert consensus
2783. Super-hydrophobic poly (lactic acid) by controlling the hierarchical structure and polymorphic transformation
2784. A Parallel Emission Regulatory Framework for Intelligent Transportation Systems and Smart Cities
2785. Liâ€“Mgâ€“Si bioceramics provide a dynamic immuno-modulatory and repair-supportive microenvironment for peripheral nerve regeneration
2786. Novel Insights Into the Zonal Flow and Transport in the Luzon Strait Based on Long-Term Mooring Observations
2787. Scalable Micro-fabrication of Flexible, Solid-State, Inexpensive, and High-Performance Planar Micro-supercapacitors through Inkjet Printing
2788. Robust initial wetness condition framework of an event-based rainfall-runoffmodel using remotely sensed soil moisture
2789. Predicting heterogeneity in clone-specific therapeutic vulnerabilities using single-cell transcriptomic signatures
2790. Simulating the daily discharge of the Mandovi river, west coast of India
2791. Psychosocial factors promoting resilience during the menopausal transition
2792. Role of Piezo2 in Schwann Cell Volume Regulation and Its Impact on Neurotrophic Release Regulation
2793. Evaluating the Reliability and Validity of the Self-Compassion Scale Short Form Adapted for Children Ages 8â€“12
2794. Effectiveness of Mindfulness Intervention in Reducing Stress and Burnout for Mental Health Professionals in Singapore
2795. 'Trait self-compassion reflects emotional flexibility through an association with high vagally mediated heart rate variability': Correction
2796. Trait Self-Compassion Reflects Emotional Flexibility Through an Association with High Vagally Mediated Heart Rate Variability
2797. Body acceptance by others: Refinement of the construct, and development and psychometric evaluation of a revised measureâ€”The Body Acceptance by Others Scale-2
2798. Development and initial validation of the Supervision Competencies Scale (SCS)
2799. Thermal Inactivation of Shiga Toxin-Producing Escherichia coli Cells within Cubed Beef Steaks following Cooking on a Griddle
2800. Psychometric properties of the Five Facets Mindfulness Questionnaire in moderate-to-severe, persistent depression
2801. A comparison of number-of-rain-days estimation techniques for continental hydrological modelling
2802. Suicide risk management in clinical practice. Current challenges and future perspectives
2803. Separate-component-stabilization system for protein and DNA production without the use of antibiotics
2804. 'Exploratory structural equation modeling analysis of the Self-Compassion Scale': Erratum
2805. Accuracy of prediction from multi-environment trials for new locations using pedigree information and environmental covariates: the case of sorghum (Sorghum bicolor (L.) Moench) breeding
2806. Evaluating evapotranspiration estimation methods in APEX model for dryland cropping systems in a semi-arid region
2807. Switching ability in bilinguals following multitasking: Impact of language distance
2808. Reliability, validity, and significance of assessment of sense of contribution in the workplace
2809. Self-consciousness and social anxiety in youth: the Revised Self-Consciousness Scales for Children
2810. Self-compassion: Evaluation of a psychoeducational website
2811. Comprehensive Review on Development of Smart Cities Using Industry 4.0 Technologies
2812. Self-Compassion and Anxiety in Adolescents with and without Anxiety Disorder
2813. Exploring the hope construct in psychotherapy
2814. A genome scan for quantitative trait loci affecting milk somatic cell score in Israeli and Italian Holstein cows by means of selective DNA pooling with single- and multiple-marker mapping
2815. Electrodes with Unique Graphite Sheet-Supported Zn-Co-S Nanoparticles for Hybrid Supercapacitors with High Performance
2816. Three-dimensional sea urchin-like MnCo2O4 nanoarchitectures on Ni foam towards high-performance asymmetric supercapacitors
2817. Revision of Self-Control Scale for Chinese college students
2818. Shunt capacitor renewal planning through cost leveling strategy using condition age model
2819. Main difficulties hindering supply chain performance: An exploratory analysis at Uruguayan SMEs
2820. Adapting and validating a measure of diabetes-specific self-compassion
2821. Diabetes-Specific Self-Compassion: A New Measure for Parents of Youth With Type 1 Diabetes
2822. [Determination of four amide synthetic cannabinoid isomers by ultra-high performance liquid chromatography-high resolution mass spectrometry]
2823. A 2-AXIS SI/AL Bimorph-Based Electrothermal Micromirror Integrated with Piezoresistors for High Resolution Position Sensing
2824. Local stress analysis of single crystalline silicon resonator using micro Raman spectroscopy
2825. Coordinated and optimized voltage control in active distribution based on two-stage programming algorithm
2826. Smart contract swarm and multi-branch structure for secure and efficient BIM versioning in blockchain-aided common data environment
2827. Spinal Cord Stimulation Attenuates Mechanical Allodynia and Increases Central Resolvin D1 Levels in Rats With Spared Nerve Injury
2828. Performance of a combined solar heating system for residential applications in Greece
2829. An approach to reduce the gap between conceptual and execution models in agent-directed simulations
2830. Is Paper Stationery or Agile? An Investigation of Dynamic Capabilities in the Printing Paper Supply Chain
2831. Regenerative medicine in Huntington's disease: Strengths and weaknesses of preclinical studies
2832. Application of HEC-HMS model for flow simulation in the lake Tana Basin: The case of Gilgel Abay Catchment, upper blue Nile Basin, Ethiopia
2833. Tool to address green roof widespread implementation effect in flood characteristics for water management planning
2834. Evaluation of salivary cortisol measurements for the diagnosis of subclinical Cushing's syndrome
2835. Glycome diagnosis of human induced pluripotent stem cells using lectin microarray
2836. Assessing oneâ€™s sense of normalcy: Psychometric properties of the Subjective Normalcy Inventory
2837. Lineage enforcement by inductive mesenchyme on adult epithelial stem cells across developmental germ layers
2838. Self-awareness and symptom evaluation: Observations from the Seattle Midlife Women's Health Study
2839. Rigorous formalism for unconventional symmetry breaking in Fermi liquid theory and its application to nematicity in FeSe
2840. Genomic predictions based on haplotypes fitted as pseudo-SNP for milk production and udder type traits and SCS in French dairy goats
2841. â€˜Sentence Crimesâ€™: Blurring the boundaries between the sentence-level accuracies and their meanings conveyed
2842. Self-supported transition metal oxide electrodes for electrochemical energy storage
2843. A linear algebraic approach to pitch-class set genera
2844. Competency development opportunities and organizational citizenship behaviors: theÂ mediating role of subjective career success
2845. Applicability of an empirical runoff estimation method in central Greece
2846. Effects of Different Retention Parameter Estimation Methods on the Prediction of Surface Runoff Using the SCS Curve Number Method
2847. Synthesis and use of bio-based dielectric substrate for implanted radio frequency antennas
2848. Neurosurgeons' Armamentarium for the Management of Refractory Postherpetic Neuralgia: A Systematic Literature Review
2849. Nonmonotonous Lattice Distortion Model for Gas Hydrates
2850. Spectral matching techniques to determine historical Land-use/Land-cover (LULC) and irrigated areas using time-series 0.1-degree AVHRR pathfinder datasets
2851. The phenomenology of selfâ€critical thinking in people with depression, eating disorders, and in healthy individuals
2852. Characterisation of the conveying effect of turned radial shaft seal counter-surfaces using aÂ simplified hydrodynamic simulation model
2853. Assessment of land use and land cover dynamics and its impact in direct runoff generation estimation using SCS CN method
2854. A Newly Synthesized Copper Redox Couple Electrolyte with Activated Carbon Electrode from Samanea saman Wood Tissue for Flexible Supercapacitor
2855. Optimization of electrocoagulation process to treat biologically pretreated bagasse effluent
2856. Child maltreatment, lifetime trauma, and mental health in Swiss older survivors of enforced child welfare practices: Investigating the mediating role of self-esteem and self-compassion
2857. Resilience and stress in later life: A network analysis approach depicting complex interactions of resilience resources and stress-related risk factors in older adults
2858. A multi-tier study on supply chain flexibility in the automotive industry
2859. A longitudinal analysis of symptom clusters in cancer patients and their sociodemographic predictors
2860. Emotional Intelligence and Academic Buoyancy in University Students: The Mediating Influence of Self-Compassion and Achievement Goals
2861. Slowing the flow in Pickering: Quantifying the effect of catchment woodland planting on flooding using the soil conservation service Curve Number method
2862. Mitigating the COVID-19 pandemic in India: an in-depth exploration of challenges and opportunities for three vulnerable population groups
2863. Childrenâ€™s social emotional competence in Pakistan and Sweden: Factor structure and measurement invariance of the Social Competence Scale (teacher edition)
2864. Children's social emotional competence in Pakistan and Sweden: Factor structure and measurement invariance of the Social Competence Scale (teacher edition)
2865. Impact of Mass Redistribution on Regional Sea Level Changes Over the South China Sea Shelves
2866. Expanding the efficacy of Project UPLIFT: Distance delivery of mindfulness-based depression prevention to people with epilepsy
2867. Patient selection for spinal cord stimulation: The importance of an integrated assessment of clinical and psychosocial factors
2868. Applicability and Validity of an e-Health Tool for the Appropriate Referral and Selection of Patients With Chronic Pain for Spinal Cord Stimulation: Results From a European Retrospective Study
2869. Self-compassion and subclinical cardiovascular disease among midlife women
2870. Timed communication buoy system: A subsurface mooring system for efficient sensor data recovery
2871. Alteration of synonymous codon usage bias accompanies polyploidization in wheat
2872. A novel discretization of the Yajima-Oikawa equation: Cauchy matrix approach
2873. Estimating montane forest above-ground biomass in the upper reaches of the Heihe River Basin using Landsat-TM data
2874. Assessment of non-point source pollution export from Zigui county in the three gorges reservoir area using the annAGNPS model
2875. High-Speed Maneuvering and Spread Target Detection in High-Resolution Radar
2876. Coherent Integration and Detection for Extended Targets in Traffic Scenes
2877. Reconfigurable Intelligent Surface-Assisted Secondary Communication System Coexisting With Multiple Primary Networks
2878. Sum-Product Network-Based Cardinality Estimation Research
2879. A novel approach to identify optimal metabotypes of elongase and desaturase activities in prevention of acute coronary syndrome
2880. An Electrical power output for n th of N-Al/Cu integrated photovoltaic thermal-module (PVT-M) collectors cum water storage system: An experimental validation
2881. Moderating Role of Supply Chain Re-engineering Capabilities on Artificial Intelligence-Based Risk Management and Supply Chain Agility: A Resource-Based View
2882. PI 2PE: Protein interface/interior prediction engine
2883. Validation of an HPLC-UV method for the determination of digoxin residues on the surface of manufacturing equipment
2884. Relational Needs Satisfaction Scale: reliability and validity study in Turkish
2885. Narcissism and concern for others: A contradiction in terms?
2886. Self-compassion: A protective factor for parents of children with autism spectrum disorder
2887. Improvement of dynamic modeling of supercapacitor by residual charge effect estimation
2888. Modelling of current and temperature effects on supercapacitors ageing. Part II: State-of-Health assessment
2889. Contributions of Industry 4.0 to supply chain resilience
2890. The continued exploration of self-acceptance: Creating a normative sample and examining the incremental validity of the FSA-SGI
2891. Modelling rainfall-discharge data at a mean annual scale in northern Algeria
2892. A multi-agent knowledge model for SMEs mechatronic supply chains
2893. Robotics Verification and Validation Strategies for Perseverance Rover Sampling and Caching
2894. Self-compassion and mindfulness: Modeling change processes associated with the reduction of perinatal depression
2895. Anxiety, depression, urinary continence, and sexuality in patients undergoing radical prostatectomy: preliminary findings
2896. Normalized Model Reference Adaptive Control Applied to High Starting Torque Scalar Control Scheme for Induction Motors
2897. Closed-Loop Adaptive High-Starting Torque Scalar Control Scheme for Induction Motor Variable Speed Drives
2898. Mesoporous Silica-Coated Gold Nanoparticles for Multimodal Imaging and Reactive Oxygen Species Sensing of Stem Cells
2899. Preliminary development and validation of a scale to measure universal love
2900. Confirmed effects of candidate variants for milk production, udder health, and udder morphology in dairy cattle
2901. Behavioural response to illness: Development and validation of a self-report measure of illness behaviour avoidance
2902. Further validation of the 18-item Portuguese CompACT scale using a multi-sample design: Confirmatory factor analysis and correlates of psychological flexibility
2903. Remote control of sea surface temperature on the variability of tropical cyclone activity affecting vietnamâ€™s coastline
2904. Main transcription factors involved in the functioning of stem cells. Ð¡haracteristics of their activation and expression in the pancreÐ°tic Î²-cells (part 2)
2905. Coupled energy management algorithm for MESS in urban EV
2906. Energy- and power-split management of dual energy storage system for a three-wheel electric vehicle
2907. Validity Beyond Measurement: Why Psychometric Validity Is Insufficient for Valid Psychotherapy Research
2908. Supply chain risk management: Manufacturing- and service-oriented firms
2909. Investigating Stable and Dynamic Aspects of the Vietnamese Self-Compassion Scale using Generalisability Theory
2910. The impact of different decision behavior models of emergency physicians on the performance of emergency departments
2911. The improved photoresponse of the substrate-free InGaN solar cells with a bottom reflector
2912. Invited paper: Beagle: A new framework for smart contracts taking account of law
2913. Embracing supply chain agility: an investigation in the electronics industry
2914. Anomalous hydrographic and biological conditions in the northern South China Sea during the 1997-1998 El NiÃ±o and comparisons with the equatorial Pacific
2915. Spinal cord injury reprograms muscle fibroadipogenic progenitors to form heterotopic bones within muscles
2916. ILLUDAS and PSRM-QUAL predictive ability in small urban areas and comparison with other models
2917. Towards the identification of important strategic priorities of the supply chain network: An empirical investigation
2918. Modelling and fault current characterization of superconducting cable with high temperature superconducting windings and copper stabilizer layer
2919. Time-Domain Protection of Superconducting Cables Based on Artificial Intelligence Classifiers
2920. Advanced fault location scheme for superconducting cables based on deep learning algorithms
2921. Understanding seasonal cycle of daily extreme temperatures based on generalized additive model for location, scale and shape with smoothing spline
2922. A novel approach in spinal cord stimulation for enhancing gastric motility: A preliminary study on canines
2923. Application of Tropical Rainfall Measuring Mission (TRMM) Data for Flood Estimation in Lack Data Catchment
2924. Using mindful self-compassion (MSC) as a strategy to reduce stress and develop self-compassion in nursing students
2925. Chinaâ€™s Sources of Power at the State Level: The Military, Economy, and National Performance
2926. Conclusion
2927. Theories of Chinese Assertiveness in the South China Sea
2928. Determining the potential link of self-compassion with eating pathology and body image among women: a longitudinal mediational study
2929. Determining the potential links of self-compassion with eating pathology and body image among women and men: A cross-sectional mediational study
2930. Determining the potential link of self-compassion with eating pathology and body image among women: A longitudinal mediational study
2931. A flow analysis system integrating an optoelectronic detector for the quantitative determination of active ingredients in pharmaceutical formulations
2932. Spinal cord stimulation: Stimulating questions
2933. Fabrication of ECM protein coated hollow collagen channels to study peripheral nerve regeneration
2934. Analysis and modeling of the seasonal South China Sea temperature cycle using remote sensing
2935. SCS-CN based time-distributed sediment yield model
2936. Perceiving beauty in all women: Psychometric evaluation of the Broad Conceptualization of Beauty Scale
2937. Assessing psychological flexibility in chronic illness
2938. Microsatellite and mitochondrial haplotype differentiation in blue mackerel (Scomber australasicus) from the western North Pacific
2939. Residual circulations and associated water mass transport in the South China sea analyzed with a coupled HYCOM-ROMS downscaling ocean model
2940. Permeance based model for the coupled-inductor utilized in the supercapacitor assisted surge absorber (SCASA) and its experimental validation
2941. Genetic aspects of immunoglobulins and cyclophilin A in milk as potential indicators of mastitis resistance in Holstein cows
2942. Resilience and agility in sustainable supply chains: A relational and dynamic capabilities view
2943. The use of latent profiles to explore the multi-dimensionality of self-compassion
2944. A negative body image contextualization of the self-compassion scale
2945. The revising of the Tangney Self-Control Scale for Chinese students
2946. Development and performance evaluation of SCS-CN based hybrid model
2947. Finite element modeling of stator winding faults in permanent magnet synchronous motor: Part II
2948. Reliability and Validity of the Turkish Version of the Acceptance and Action Questionnaire-Substance Abuse (AAQ-SA) on a Clinical Sample
2949. Cross-lagged analyses between life meaning, self-compassion, and subjective well-being among gifted adults
2950. Towards a unified framework for modelling the Canadian forest products value Chain
2951. Bootstrapping Trust inÂ Community Repository Projects
2952. SCARDEC: A new technique for the rapid determination of seismic moment magnitude, focal mechanism and source time functions for large earthquakes using body-wave deconvolution
2953. Genomics of the Effect of Spinal Cord Stimulation on an Animal Model of Neuropathic Pain
2954. Factors associated with smoking cessation in exclusive smokers and dual users of e-cigarette and conventional cigarettes from CDTnet registry
2955. Climate projections of precipitation and temperature in cities from ABC Paulista, in the Metropolitan Region of SÃ£o Pauloâ€”Brazil
2956. Urban climate assessment in the ABC Paulista Region of Sao Paulo, Brazil
2957. Twelve-week yoga vs Aerobic cycling initiation in sedentary healthy subjects: A behavioral and multiparametric interventional PET/MR study
2958. Self-compassion is a better predictor than mindfulness of symptom severity and quality of life in mixed anxiety and depression
2959. External validation of two prediction models of complete secondary cytoreductive surgery in patients with recurrent epithelial ovarian cancer
2960. Validation of scalp coverage scoring methods for scalp hair loss in male pattern hair loss (androgenetic alopecia)
2961. Validation and clinical relevance of a novel scalp coverage scoring method
2962. Effects of mindfulness training on perceived stress, self-compassion, and self-reflection of primary care physicians: a mixed-methods study
2963. Preventing eating disorders in young women: An RCT and mixed-methods evaluation of the peer-delivered Body Project
2964. Self-compassion, mindfulness and their relationship to depression and anxiety in individuals diagnosed with a psychotic disorder
2965. An MPI-based implementation of Intelligent Agents on clusters
2966. "Fluctuoscopy" of superconductors
2967. The novel paradigm of economics driven for local smart sustain cities modeling using exploratory factor analysis and planning technique using fuzzy evaluation decision making
2968. Genomic prediction using preselected DNA variants from a GWAS with whole-genome sequence data in Holstein-Friesian cattle
2969. A three level abstraction hierarchy to represent product structural information
2970. Attempt to rescue discarded human liver grafts by end ischemic hypothermic oxygenated machine perfusion
2971. Evaluating the effects of forest fire on water balance using fire susceptibility maps
2972. An item response theory analysis of the sexual compulsivity scale and its correspondence with the hypersexual disorder screening inventory among a sample of highly sexually active gay and bisexual men
2973. Additive Manufacturing for Localized Medical Parts Production: A Case Study
2974. How the mindfulness manifold relates to the five moral foundations, prejudice, and awareness of privilege
2975. Application of HEC-HMS for Hydrological Modeling of Upper Sabarmati River Basin, Gujarat, India
2976. A Study of Dependability Modeling Methodologies for Safety-Critical Systems
2977. Improved runoff curve numbers for a large number of watersheds of the USA
2978. SCS-CN methodology further modified
2979. Triguna (three qualities) personality model and two-factor conceptualization of self-compassion: A new insight to understand achievement goal orientations
2980. Efficiency of genomic prediction of non-assessed single crosses
2981. Analysis of genetic correlations between multivariate measures of lactation persistency and somatic cell score in Italian Simmental cattle
2982. Development of mindfulness in relational context: Construction and validation of relational mindfulness training (RMT)
2983. A relativistic DFT methodology for calculating the structures and NMR chemical shifts of octahedral platinum and iridium complexes
2984. Bypassing data issues of a supply chain simulation model in a big data context
2985. On the use of simulation as a Big Data semantic validator for supply chain management
2986. A centralized platform of open government data as support to applications in the smart cities context
2987. Percoll discontinuous density gradient centrifugation method for the fractionation of the subpopulations of Mycobacterium smegmatis and Mycobacterium tuberculosis from in vitro cultures
2988. Conversion of Solar Energy into Electrical Energy Storage: Supercapacitor as an Ultrafast Energy-Storage Device Made from Biodegradable Agar-Agar as a Novel and Low-Cost Carbon Precursor
2989. Safety Practices in Requirements Engineering: The Uni-REPM Safety Module
2990. Assessment of safety processes in requirements engineering
2991. Specifying Safety Requirements with GORE languages
2992. Sleep clinical record: an aid to rapid and accurate diagnosis of paediatric sleep disordered breathing
2993. Adequacy and Acceptability of the Self-Collected Anal Pap Smear in People Living With Human Immunodeficiency Virus in the Infectious Diseases Clinic
2994. [Validation of the 12 items self-compassion scale in Chile]
2995. Shape Constrained Splines with Discontinuities for Anomaly Detection in a Batch Process
2996. Genetic expression profile of olfactory ensheathing cells is distinct from that of Schwann cells and astrocytes
2997. The impact of group-based mindfulness training on self-reported mindfulness: A systematic review and meta-analysis
2998. Romanian River Basins Lag Time Analysis. The SCS-CN Versus RNS Comparative Approach Developed for Small Watersheds
2999. Eco-pickled surface: An environmentally advantageous alternative to conventional acid pickling
3000. Crisis Concept Re-loaded?-The Recently Described Suicide-Specific Syndromes May Help to Better Understand Suicidal Behavior and Assess Imminent Suicide Risk More Effectively
3001. Self-compassion and negative outgroup attitudes: The mediating role of compassion for others
3002. Cyberchondria and questionable health practices: The mediation role of conspiracy mentality
3003. Robust Co3O4|Î±-Al2O3|cordierite structured catalyst for N2O abatement â€“ Validation of the SCS method for active phase synthesis and deposition
3004. Application of modified SME-CN method for predicting event runoff and peak discharge from a drained forest watershed on the North Carolina Atlantic coastal plain
3005. Assessing runoff using modified SME-CN method for a drained forest watershed on North Carolina Atlantic Coastal Plain
3006. Transit amplifying cells coordinate mouse incisor mesenchymal stem cell activation
3007. Development and method validation of a sampling technique for a reproducible detection of synthetic cannabinoids in exhaled breath using an in vitro pig lung model
3008. Mitigating Spinal Cord Stimulator Lead Migration Complications in Minimally Invasive Spine Surgery: Technical Note
3009. Self-compassion, social rank, and psychological distress in athletes of varying competitive levels
3010. An Early Fault Diagnosis Method of Rolling Bearings on the Basis of Adaptive Frequency Window and Sparse Coding Shrinkage
3011. Game Theoretic-Based Distributed Charging Strategy for PEVs in a Smart Charging Station
3012. [Validation of the Chinese-version Self-Efficacy in Smoking Cessation Service Scale (SE-SCS) for clinical psychiatric professionals]
3013. A virtual peer mentoring intervention for baccalaureate nursing students: A mixed-methods study
3014. Adaptive Energy Estimation for Supercapacitor Based on a Real-Time Voltage State Observer in Electric Vehicle Applications
3015. Definition of South China Sea monsoon onset and commencement of the East Asian summer monsoon
3016. Validity and reliability of the Chinese Version of the Eating Attitudes Test in Chinese college students
3017. Equivalent circuits of MMC and VSC: Applications
3018. Ecological risk assessment of organochlorine pesticide mixture in South China Sea and East China Sea under the effects of seasonal changes and phase-partitioning
3019. Organic matter in surface sediments from the Gulf of Mexico and South China Sea: Compositions, distributions and sources
3020. Inverted design of oxygen vacancies modulated NiCo2O4 and Co3O4 microspheres with superior specific surface area as competitive bifunctional materials for supercapacitor and hydrogen evolution reaction
3021. A region-dependent seasonal forecasting framework for tropical cyclone genesis frequency in the western North Pacific
3022. Phase partitioning effects on seasonal compositions and distributions of terrigenous polycyclic aromatic hydrocarbons along the South China Sea and East China Sea
3023. The Construction and Analysis of lncRNA-miRNA-mRNA Competing Endogenous RNA Network of Schwann Cells in Diabetic Peripheral Neuropathy
3024. Beyond empathy decline: Do the barriers to compassion change across medical training?
3025. Advances of electrospun Mo-based nanocomposite fibers as anode materials for supercapacitors
3026. A one-parameter Budyko model for water balance captures emergent behavior in darwinian hydrologic models
3027. Validation of satellite-derived daily latent heat flux over the South China Sea, compared with observations and five products
3028. A thermodynamic interpretation of Budyko and L'vovich formulations of annual water balance: Proportionality Hypothesis and maximum entropy production
3029. Molecular Structure of the Long Periodicity Phase in the Stratum Corneum
3030. Sedimentary Budget and Controlling Factors of the Northwest and Southwest Sub-Basins, the South China Sea
3031. Feasibility of reconstructing the summer basin-scale sea surface partial pressure of carbon dioxide from sparse in situ observations over the South China Sea
3032. Modification of SCS-CN model for estimating event rainfall runoff for small watersheds in the Loess Plateau, China
3033. Modeling the impact of soil and water conservation on surface and ground water based on the SCS and visual modflow
3034. The Printed-Circuit-Board Electroplating Parallel-Tank Scheduling with Hoist and Group Constraints Using a Hybrid Guided Tabu Search Algorithm
3035. Validation of the multi-satellite merged sea surface salinity in the South China Sea
3036. Microarray analysis of the expression profile of lncRNAs reveals the key role of lncRNA BC088327 as an agonist to heregulin-1Î²-induced cell proliferation in peripheral nerve injury
3037. Microarray analysis of the expression profile of lncRNAs reveals the key role of lncRNA BC088327 as an agonist to heregulin-1beta-induced cell proliferation in peripheral nerve injury
3038. Improved Algorithm of SCS-CN Model Parameters in Typical Inland River Basin in Central Asia
3039. Calibration of SCS model parameters regarding snowmelt season in Xinjiang Kuitun River basin
3040. The development and structure of an oceanic squall line systems during the South China sea monsoon experiment
3041. Radar HRRP target recognition via statistics-based scattering centre set registration
3042. Numerical Simulation and Observational Data Analysis of Mesoscale Eddy Effects on Surface Waves in the South China Sea
3043. Efficacy of 93 cases of recurrent ovarian cancer
3044. Runoff simulation based on SCS mode in Bortala River Basin in Xinjiang
3045. Safety assessment of shipping routes in the South China Sea based on the fuzzy analytic hierarchy process
3046. Selecting optimal calibration samples using proximal sensing EM induction and Î³-ray spectrometry data: An application to managing lime and magnesium in sugarcane growing soil
3047. Selecting optimal calibration samples using proximal sensing EM induction and gamma-ray spectrometry data: An application to managing lime and magnesium in sugarcane growing soil
3048. A modulating model for the impacting factors in self-regulated learning of college students
3049. Ultraviolet B Irradiation Alters the Level and miR Contents of Exosomes Released by Keratinocytes in Diabetic Condition
3050. Evaluating Production of Cyclopentyl Tetraethers by Marine Group II Euryarchaeota in the Pearl River Estuary and Coastal South China Sea: Potential Impact on the TEX86 Paleothermometer
3051. A Novel Seamless Commutation Switch for Three-phase Unbalanced Current
3052. DNA methylation haplotype block signatures responding to Staphylococcus aureus subclinical mastitis and association with production and health traits
3053. Imaging of structure at and near the core mantle boundary using a generalized radon transform: 1. Construction of image gathers
3054. Optimum placement of distributed generation resources, capacitors and charging stations with a developed competitive algorithm
3055. Enhancement of voltage quality in isolated power systems
3056. Bioprinted constructs that simulate nerveâ€“bone crosstalk to improve microenvironment for bone repair
3057. Developments of steel-concrete-steel sandwich composite structures with novel EC connectors: Members
3058. Physical vulnerability curve construction and quantitative risk assessment of a typhoon-triggered debris flow via numerical simulation: A case study of Zhejiang Province, SE China
3059. Revisiting the Intraseasonal Variability of Chlorophyll-a in the Adjacent Luzon Strait with a New Gap-Filled Remote Sensing Data Set
3060. Submarine landslides on the north continental slope of the South China Sea
3061. New Three-Phase Current Reconstruction for PMSM Drive with Hybrid Space Vector Pulsewidth Modulation Technique
3062. Let-7a-5p regulated by lncRNA-MEG3 promotes functional differentiation to Schwann cells from adipose derived stem cells via directly inhibiting RBPJ-mediating Notch pathway
3063. The summer-fall anticyclonic eddy west of Luzon: Structure and evolution in 2012 and interannual variability
3064. Influences of potential evapotranspiration estimation methods on SWAT's hydrologic simulation in a northwestern Minnesota watershed
3065. Decomposing service conveniences in self-collection: An integrated application of the SERVCON and Kano models
3066. Applicability Analysis of Different Runoff Schemes of the Gulang River Basin in Arid Region of Gansu Province, China
3067. Validation and Evaluation of GRACE-FO Estimates with In Situ Bottom Pressure Array Measurements in the South China Sea
3068. Storm-event rainfall-runoff modelling approach for ungauged sites in Taiwan
3069. BUCKLING BEHAVIOUR OF THE STEEL PLATE IN STEEL â€“ CONCRETE â€“ STEEL SANDWICH COMPOSITE TOWER FOR WIND TURBINE
3070. Impact response of steel-PU foam-steel-concrete-steel panel: Experimental, numerical and analytical studies
3071. Decentralized Cross-Layer Optimization for Energy-Efficient Resource Allocation in HetNets
3072. Designing a new robust solid waste recycling network under uncertainty: A case study about circular economy transition
3073. Relationship of self-control and perception of social support with mental health status of university students during the normalization of COVID-19 prevention and control
3074. In-band scattering reduction of phased array by loading artificial electromagnetic materials
3075. Experimental and numerical studies on novel stiffener-enhanced steel-concrete-steel sandwich panels subjected to impact loading
3076. Large-scale seismic seafloor stability analysis in the South China Sea
3077. Binary doping of nitrogen and phosphorus into porous carbon: A novel di-functional material for enhancing CO2 capture and super-capacitance
3078. Development of dimensionless P-I diagram for curved SCS sandwich shell subjected to uniformly distributed blast pressure
3079. Symptom clusters and impact on quality of life in esophageal cancer patients
3080. Single-Step Preparation of Ultrasmall Iron Oxide-Embedded Carbon Nanotubes on Carbon Cloth with Excellent Superhydrophilicity and Enhanced Supercapacitor Performance
3081. Quantification of MDMB-4en-PINACA and ADB-BUTINACA in human hair by gas chromatography-tandem mass spectrometry
3082. A preliminary study of Self-constructionals Scales of Chinese-version
3083. Extreme wave climate variability in South China Sea
3084. An Improved Hybrid MMC with Thyristor-Switched Supercapacitors for Fault Ride-Through Support
3085. Spatial reconstruction of long-term (2003-2020) sea surface pCO2 in the South China Sea using a machine-learning-based regression method aided by empirical orthogonal function analysis
3086. Reconstruction of High-Resolution Sea Surface Salinity over 2003â€“2020 in the South China Sea Using the Machine Learning Algorithm LightGBM Model
3087. A multiple kernel ensemble approach for genomic prediction
3088. The influence of the types of cluster composite abrasives on the performance of fixed abrasive pads in processing quartz glass
3089. A liquid chromatography-tandem mass spectrometry (LC-MS/MS)-based assay to profile 20 plasma steroids in endocrine disorders
3090. Resection or degeneration of uncovertebral joints altered the segmental kinematics and load-sharing pattern of subaxial cervical spine: A biomechanical investigation using a C2-T1 finite element model
3091. Single-Cell Sequencing-Enabled Hexokinase 2 Assay for Noninvasive Bladder Cancer Diagnosis and Screening by Detecting Rare Malignant Cells in Urine
3092. Simulation of flood inundation of Guiyang city using remote sensing, GIS and hydrologic model
3093. Acceleration-based design auxiliary power source for Electric Vehicle applications
3094. Acceleration-based design of electric vehicle auxiliary energy source
3095. The relationships between addiction-related personality traits, self-compassion, and PTSD treatment target variables in a clinical sample
3096. Examining sense of community in sport: Developing the multidimensional 'SCS' scale
3097. Psychometric evaluation of the Five Facet Mindfulness Questionnaire in a clinical sample of African Americans
3098. Racismâ€™s (un)worthiness trap: The mediating roles of self-compassion and self-coldness in the link between racism and distress in African Americans
3099. Mindfulness-based compassion training for health professionals providing end-of-life care: Impact, feasibility, and acceptability
3100. The Quiet Ego Scale: Measuring the compassionate self-identity
3101. Development and validation of the Equanimity Barriers Scale [EBS]
3102. Exploring the relationship between fear of positive evaluation and social anxiety
3103. 3D boron/nitrogen dual doped layered carbon for 2 V aqueous symmetric supercapacitors
3104. Opposite variability of indonesian throughflow and south china sea throughflow in the Sulawesi Sea
3105. Attachment, selfâ€compassion, empathy, and subjective wellâ€being among college students and community adults
3106. The effect of self-compassion on social media addiction among college students - The mediating role of gratitude: An observational study
3107. An improved method for isolating Schwann cells from postnatal rat sciatic nerves
3108. Baseband cross-poarization interference cancellation based on scs algorithm
3109. Competitive-level differences on sport commitment among high school-and collegiate-level athletes
3110. A real-time non-intrusive load monitoring system
3111. Robust Non-Intrusive Load Monitoring (NILM) with unknown loads
3112. News Event Prediction using Causality Approach on South China Sea Conflict
3113. Mindfulness processes that mitigate COVID-related stressors in caregivers in the United States
3114. Energy flux variations and safety assessment of offshore wind and wave resources during typhoons in the northern South China Sea
3115. The relation between psychological flexibility and the Buddhist practices of meditation, nonattachment, and self-compassion
3116. Identification of seizure clusters using free text notes in an electronic seizure diary
3117. A new scrupulosity scale for the Dimensional Obsessive- Compulsive Scale (DOCS): Validation with clinical and nonclinical samples
3118. The development of the â€˜Forms of Responding to Self-Critical Thoughts Scaleâ€™ (FoReST)
3119. The relative benefits of nonattachment to self and selfâ€compassion for psychological distress and psychological wellâ€being for those with and without symptoms of depression
3120. A Factor Analysis of the Suicide Cognitions Scale in Veterans with Military Sexual Trauma-Related Posttraumatic Stress Disorder
3121. The impact of different peripheral suture techniques on the biomechanical stability in flexor tendon repair
3122. Incorporating surface storage and slope to estimate clark unit hydrographs for ungauged Indiana watersheds
3123. Validation of the Comprehensive Inventory of Mindfulness Experiences (CHIME) in English using Rasch methodology
3124. Prior depression affects the experience of the perimenopauseâ€”findings from the Swiss Perimenopause Study
3125. Verifying Mars 2020 Sampling and Caching Robotic Functions with Position Budgeting Process and Tool
3126. Staying well after depression: Trial design and protocol
3127. Spinal cord stimulation: "neural switch" in complex regional pain syndrome type I
3128. Characterisations of human prostate stem cells reveal deficiency in class I UGT enzymes as a novel mechanism for castration-resistant prostate cancer
3129. Vividness of positive mental imagery predicts positive emotional response to visually presented Project soothe pictures
3130. Using SWAT for sub-field identification of phosphorus critical source areas in a saturation excess runoff region
3131. Benefits of inferential statistical methods in radiation exposure studies: another look at percutaneous spinal cord stimulation mapping [trialing] procedures
3132. Is self-compassion negatively associated with alcohol and marijuana-related problems via coping motives?
3133. A comparative study of the dimensionality of the Self-Concealment Scale using principal components analysis and Mokken scale analysis
3134. Genetic evaluations for endangered dual-purpose German Black Pied cattle using 50K SNPs, a breed-specific 200K chip, and whole-genome sequencing
3135. How self-compassion moderates the relation between body surveillance and body shame among men and women
3136. Reliability, validity and effectiveness of strain counterstrain techniques
3137. Differentiating the role of three self-compassion components in buffering cognitive-personality vulnerability to depression among Chinese in Hong Kong
3138. Development and validation of the Social Ecological Resilience Scale (SERS) from a systems perspective for Hong Kong families
3139. Analysis of symptom clusters in patients with brain metastases with three different statistical methods
3140. Analysis of symptom clusters in patients with brain metastases
3141. Analysis of symptom clusters in patients with brain metastases with three different statistical methods
3142. Artificial intelligence-driven risk management for enhancing supply chain agility: A deep-learning-based dual-stage PLS-SEM-ANN analysis
3143. A selection of a fabric-cutting system configuration in different types of apparel manufacturing environments
3144. Comparing self-compassion, mindfulness, and psychological inflexibility as predictors of psychological health
3145. Reliability and validity of the Chinese version of the Social Connectedness Scale
3146. Heat-Resistant, Robust, and Hydrophilic Separators Based on Regenerated Cellulose for Advanced Supercapacitors
3147. Statistical compressed sensing based on Bayesian principal component analysis
3148. Applicability of swat for three coastal watersheds in Louisiana
3149. Evaluation of the applicability of the SWAT model for coastal watersheds in southeastern Louisiana
3150. Simultaneous detection of 87 synthetic cannabinoids and their metabolites in urine samples by UHPLC-MS/MS and application to 109 authentic forensic cases
3151. Feature tracking compared with tissue tagging measurements of segmental strain by cardiovascular magnetic resonance
3152. Predicting the retention time of Synthetic Cannabinoids using a combinatorial QSAR approach
3153. On modeling and simulation of game theory-based defense mechanisms against DoS and DDoS attacks
3154. Impact of Typhoon Kalmaegi (2014) on the South China Sea: Simulations using a fully coupled atmosphere-ocean-wave model
3155. Self-Driven Dual-Path Learning for Reference-Based Line Art Colorization under Limited Data
3156. Developing measures for assessing the causality of safety culture in a petrochemical industry
3157. Design and control of a combined BDC based energy storage system for regenerative applications
3158. Interdecadal variability of early summer monsoon rainfall over South China in association with the Pacific Decadal Oscillation
3159. Lymph Node Dissection Is a Risk Factor for Short-Term Cough after Pulmonary Resection
3160. Shunt active power filter synthesiszing resistive loads based on adaptive inverse control
3161. An Efficient Method on ISAR Image Reconstruction via Norm Regularization
3162. Modeling hydrological responses of karst spring to storm events: Example of the Shuifang spring (Jinfo Mt., Chongqing, China)
3163. Adaptive Authority Allocation of Human-Automation Shared Control for Autonomous Vehicle
3164. Shunt active power filter synthesizing resistive loads by means of adaptive inverse control
3165. Simulation of the storm surge in the South China Sea based on the coupled sea-air model
3166. A criterion based on computational singular perturbation for the construction of a reduced mechanism for dimethyl ether oxidation
3167. Application of SCS model on computing the surface runoff of different land use types-A case study on Yuyao, Zhejiang
3168. Unleashing the Potential of Siliciclastic Reservoirs in the Sepinggan Carbonate Sequences, A Discovery in the South Mahakam Field, Lower Kutai Basin
3169. Long-term and interannual variation of the steric sea level in the South China Sea and the connection with ENSO
3170. Distribution of 3-hydroxy fatty acids in South China Sea since the last deglaciation: Applicability of 3-hydroxy fatty acid-based palaeothermometry
3171. Mechanical stimulation of Schwann cells promote peripheral nerve regeneration via extracellular vesicle-mediated transfer of microRNA 23b-3p
3172. Crustal structure and features in the conjugate margins of South China Sea
3173. Synthesis and bioactivity of erythro-nordihydroguaiaretic acid, threo-(-)-saururenin and their analogues
3174. Analyzing Critical Factors for the Smart Construction Site Development: A DEMATEL-ISM Based Approach
3175. Effect of combination of acellular nerve grafts and stem cells for sciatic nerve regeneration: A meta-analysis
3176. Research on the High Frequency Oscillation of MMC-HVDC Integrated into Renewable Energy System
3177. Temporal and tight hepatitis C virus gene activation in cultured human hepatoma cells mediated by a cell-permeable Cre recombinase
3178. Parametric Scattering Center Modeling for a Conducting Deep Cavity
3179. Parametric Scattering Center Modeling for the Complex Conducting Cavity
3180. In-Band Scattering Reduction of Wideband Phased Antenna Arrays with Enhanced Coupling Based on Phase-Only Optimization Techniques
3181. Supercontinuum generation based on all-normal-dispersion Yb-doped fiber laser mode-locked by nonlinear polarization rotation: Influence of seed's output port
3182. Incorporating transportation safety into land use planning: Pre-assessment of land use conversion effects on severe crashes in urban China
3183. Reconstructing three-dimensional salinity field of the South China Sea from satellite observations
3184. An eddy resolving tidal-driven model of the South China Sea assimilating along-track SLA data using the EnOI
3185. Impact of assimilating altimeter data on eddy characteristics in the South China Sea
3186. Verification of a New Spatial Distribution Function of Soil Water Storage Capacity Using Conceptual and SWAT Models
3187. Coral skeletons reveal the impacts of oil pollution on seawater chemistry in the northern South China Sea
3188. Integrated Risk Assessment of Waterlogging in Guangzhou Based on Runoff Modeling, AHP, GIS and Scenario Analysis
3189. Peak-summer East Asian rainfall predictability and prediction part I: Southeast Asia
3190. A systematic approach for multi-objective lightweight and stiffness optimization of a car body
3191. TNF-Î±/ENO1 signaling facilitates testicular phagocytosis by directly activating Elmo1 gene expression in mouse Sertoli cells
3192. TNF-alpha/ENO1 signaling facilitates testicular phagocytosis by directly activating Elmo1 gene expression in mouse Sertoli cells
3193. MicroRNA-34b-5p targets PPP1R11 to inhibit proliferation and promote apoptosis in cattleyak Sertoli cells by regulating specific signaling pathways
3194. Research on the competition between two completely separated SCs and coordination within one SC with price dependent stochastic demand
3195. Research on supply chain versus supply chain competition with product quality and price dependent demand
3196. Suprachoroidal Delivery of Subretinal Gene and Cell Therapy
3197. Determination of 5 synthetic cannabinoids in hair by Segmental analysis using UHPLC-MS/MS and its application to eight polydrug abuse cases
3198. State analysis using the Local Ensemble Transform Kalman Filter (LETKF) and the three-layer circulation structure of the Luzon Strait and the South China Sea Topical Collection on the 5th International Workshop on Modelling the Ocean (IWMO) in Bergen, Norway 17-20 June 2013
3199. Research and development of SCS-500L power oscillation islanding control device
3200. On-Board Absolute Radiometric Calibration and Validation Based on Solar Diffuser of HY-1C SCS
3201. Onboard spectral calibration and validation of the satellite calibration spectrometer on HY-1C
3202. Onboard absolute radiometric calibration and validation of the satellite calibration spectrometer on HY-1C
3203. Flexible Carbon Cloth Supercapacitors with High Power and Energy Densities
3204. Achieving Ultrahigh Energy Densities of Supercapacitors with Porous Titanium Carbide/Boron-Doped Diamond Composite Electrodes
3205. Mechanistic Study of 1,2-Dichloroethane Hydrodechlorination on Cu-Rich Pt-Cu Alloys: Combining Reaction Kinetics Experiments with DFT Calculations and Microkinetic Modeling
3206. Addressing psychosomatic symptom distress with mindfulness-based cognitive therapy in somatic symptom disorder: mediating effects of self-compassion and alexithymia
3207. Single-Cell RNA-Seq Reveals Heterogeneity of Cell Communications between Schwann Cells and Fibroblasts within Vestibular Schwannoma Microenvironment
3208. Identification of symptom clusters and change trajectories in patients with acute exacerbation of chronic obstructive pulmonary disease
3209. Constructing trustworthy and safe communities on a blockchain-enabled social credits system
3210. Sensitivity of a carbon and productivity model to climatic, water, terrain, and biophysical parameters in a Rocky Mountain watershed
3211. Remaining useful life prediction based on spatiotemporal autoencoder
3212. Properties of Electrospun Aligned Poly(lactic acid)/Collagen Fibers with Nanoporous Surface for Peripheral Nerve Tissue Engineering
3213. Resource Allocation in Heterogeneous Cognitive Radio Network with Non-Orthogonal Multiple Access
3214. Flexible carbon membrane supercapacitor based on Î³-cyclodextrin-MOF
3215. Rainfall characteristics and convective properties of Mei-Yu precipitation systems over South China, Taiwan, and the South China sea. Part I: TRMM observations
3216. Atlas of metabolism reveals palmitic acid results in mitochondrial dysfunction and cell apoptosis by inhibiting fatty acid Î²-oxidation in Sertoli cells
3217. Optical Anapole Modes of the Hybrid Ring-Disk Nanoantenna for Electric Field Enhancement
3218. A Silk Fibroin/Collagen Nerve Scaffold Seeded with a Co-Culture of Schwann Cells and Adipose-Derived Stem Cells for Sciatic Nerve Regeneration
3219. Rapid Screening of 18 Synthetic Cannabinoids Using Atmospheric Pressure Solids Analysis Probe Coupled With Single-Quadrupole Mass Spectrometer
3220. Epidemiology of and prognostic factors for patients with sarcomatoid carcinoma: a large population-based study
3221. Skin derived precursors induced Schwann cells mediated tissue engineering-aided neuroregeneration across sciatic nerve defect
3222. Research on runoff sub-model of non-point source pollution model
3223. Vibrational Power Flow Analysis for the Sandwich Cylindrical Shell Structure with a Metalâ€“Rubber Core in the Thermal Environment
3224. Single-cell analyses reveal novel molecular signatures and pathogenesis in cutaneous T cell lymphoma
3225. Seasonal Variation of Terrigenous Polycyclic Aromatic Hydrocarbons along the Marginal Seas of China: Input, Phase Partitioning, and Ocean-Current Transport
3226. Corrigendum on: A validation study of the self-compassion scale-short form (SCS-SF) with ant colony optimization in a Turkish sample
3227. Transformative power of friendships: Examining the relationships among friendship quality, selfâ€change, and wellâ€being
3228. Generating artificial sensations with spinal cord stimulation in primates and rodents
3229. Efficacy of a Skin Condition-Adapted Solution for Xerosis and Itch Relief Associated With Aging
3230. Develop Computational Intelligence Model for Runoff Prediction in Remote Basins
3231. Dynamic simulation of battery/supercapacitor hybrid energy storage system for the electric vehicles
3232. Performance Analysis of Battery/Supercapacitor Hybrid Energy Source for the City Electric Buses and Electric Cars
3233. Laminin-332 regulates differentiation of human interfollicular epidermal stem cells
3234. A Synchronous Condenser System Capable of Eliminating Harmonic Distortion for Grid-Connected Inverters
3235. Construction of hierarchical porous derived from the cellulose nanofiber / graphene / Zn/Co ZIF 3D conductive carbon aerogels for high-performance supercapacitors
3236. Do sustainability concerns play a key role in influencing individualsâ€™ acceptance after experiencing shared autonomous vehicles? A field experiment in china
3237. Finite element analysis on steelâ€“concreteâ€“steel sandwich beams
3238. Experimental and numerical studies on ultimate strength behaviour of SCS sandwich beams with UHPFRC
3239. Behaviours of square UHPFRC-filled steel tubular stub columns under eccentric compression
3240. Finite element analysis for flexural behaviours of SCS sandwich beams with novel enhanced C-channel connectors
3241. Numerical and parametric studies on SCS sandwich walls subjected to in-plane shear
3242. Design and behavior of steel-concrete-steel sandwich plates subject to concentrated loads
3243. Ultimate strength behavior of steel-concrete-steel sandwich beams with ultra-lightweight cement composite, part 2: Finite element analysis
3244. Punching shear resistance of steel-concrete-steel sandwich composite shell structure
3245. Steel-concrete-steel sandwich system in Arctic offshore structure: Materials, experiments, and design
3246. Damage plasticity based numerical analysis on steel-concrete-steel sandwich shells used in the Arctic offshore structure
3247. Shear-tension interaction strength of j-hook connectors in steel-concrete-steel sandwich structure
3248. Punching shear behavior of steel-concrete-steel sandwich composite plate under patch loads
3249. Compressive behaviours of circular concrete-filled steel tubes exposed to low-temperature environment
3250. Compressive behaviour of normal weight concrete confined by the steel face plates in SCS sandwich wall
3251. Compressive behaviours of novel SCS sandwich composite walls with normal weight concrete
3252. Compressive behaviours of steel-concrete-steel sandwich walls with J-hooks at low temperatures
3253. Behaviour of steel-concrete-steel sandwich plates under different ice-contact pressure
3254. Numerical and parametric study of curved steel-concrete-steel sandwich composite beams under concentrated loading
3255. Cyclic tests on novel steel-concrete-steel sandwich shear walls with boundary CFST columns
3256. Seismic behaviours of SCS sandwich shear walls using J-hook connectors
3257. Numerical analysis on steel-concrete-steel sandwich plates by damage plasticity model: From materials to structures
3258. Numerical studies on shear resistance of headed stud connectors in different concretes under Arctic low temperature
3259. Interface monitoring of steel-concrete-steel sandwich structures using piezoelectric transducers
3260. The Novel-m0230-3p miRNA Modulates the CSF1/CSF1R/Ras Pathway to Regulate the Cell Tight Junctions and Blood-Testis Barrier in Yak
3261. Design and Implementation of a Lightweight Deep CNN-Based Plant Biometric Authentication System
3262. Global change of microRNA expression induced by vitamin C treatment on immature boar Sertoli cells
3263. Machine learning guided 3D printing of carbon microlattices with customized performance for supercapacitive energy storage
3264. Coordination Control for a PEMFC-Battery-Supercapacitor Hybrid Tramway
3265. Gastrodin modified polyurethane conduit promotes nerve repair via optimizing Schwann cells function
3266. Blockchain for supply chain performance and logistics management
3267. Ti3C2Tx/carbon nanotube/porous carbon film for flexible supercapacitor
3268. Toward a mesoscale hydrological and marine meteorological observation network in the South China Sea
3269. [Isolation, culture and validation of CD34+ vascular wall-resident stem cells from mice]
3270. Fabrication of three-dimensional hierarchical NiCo-LDH micro-flowers for enhanced charge storage in battery-type supercapacitors
3271. Rational intramolecular and interface design of cellulosic paper electrode via PEDOT with AQS as dopant and electrolyte additives
3272. Ecological risk assessment of the southern Fujian Golden Triangle in China based on regional transportation development
3273. Deep current evolution in the central part of the South China Sea since the Middle to Late Pleistocene
3274. Risk analysis for the modification in automatic train control systems
3275. Electronic states and quantum transport in bilayer graphene Sierpinski-carpet fractals
3276. Secure cloud storage based on rlwe problem
3277. An investigation of self-compassion and nonattachment to self in people with bipolar disorder
3278. Can self-compassion promote gratitude and prosocial behavior in adolescents? A 3-year longitudinal study from China
3279. A comprehensive LC-MS/MS method for simultaneous analysis of 65 synthetic cannabinoids in human hair samples and application to forensic investigations
3280. Biomimetic nerve guidance conduit containing engineered exosomes of adipose-derived stem cells promotes peripheral nerve regeneration
3281. Identification of Symptom Clusters and Their Influencing Factors in Subgroups of Chinese Patients With Acute Exacerbation of Chronic Obstructive Pulmonary Disease
3282. Scalable one-step synthesis of reduced graphene oxide: Towards flexible transparent conductive films and active supercapacitor electrodes
3283. Scheduling optimisation of co-operator selection and task allocation in mass customisation supply chain based on collaborative benefits and risks
3284. Influencing factors analysis of fear of progression in cervical cancer patients
3285. Predictive Validity of Proposed Diagnostic Criteria for the Suicide Crisis Syndrome: An Acute Presuicidal State
3286. Hybrid energy storage system control strategy to smooth power fluctuations in microgrids containing photovoltaics
3287. A Three-Component Model of phytoplankton size classes for the South China Sea
3288. Upwelling impact and lateral transport of dissolved PAHs in the Taiwan Strait and adjacent South China Sea
3289. New Spectrally Constrained Sequence Sets with Optimal Periodic Cross-Correlation
3290. Minimum clinical important difference for resilience scale specific to cancer: A prospective analysis
3291. Development of biomimetic micro-patterned device incorporated with neurotrophic gradient and supportive Schwann cells for the applications in neural tissue engineering
3292. Using Rasch KIDMAP to identify whether China dominates the research area of computer science (CS) based on the specialization index of article citations: Bibliometric analysis
3293. Consistency of symptom clusters among advanced cancer patients seen at an outpatient supportive care clinic in a tertiary cancer center
3294. The indian mackerel aggregation areas in relation to their oceanographic conditions
3295. The compassionate journey: An exploratory and controlled study assessing selfâ€compassionâ€focussed mindfulness in a nonclinical sample
3296. Redox electrolyte mediated performance enhancement in aqueous zinc ion hybrid supercapacitors composed of spinel BaFe2O4 and cubic Cu2O
3297. A review of niobium oxides based nanocomposites for lithium-ion batteries, sodium-ion batteries and supercapacitors
3298. Electrochemical activation and capacitance enhancement of expanded mesocarbon microbeads for high-voltage, symmetric supercapacitors
3299. 3D hierarchical Ti3C2Tx@NiCo2S4-RGO heterostructure hydrogels as free-standing electrodes for high-performance supercapacitors
3300. PLC: A simple and semi-physical topographic correction method for vegetation canopies based on path length correction
3301. A Triple-Classification Radiomics Model for the Differentiation of Primary Chordoma, Giant Cell Tumor, and Metastatic Tumor of Sacrum Based on T2-Weighted and Contrast-Enhanced T1-Weighted MRI
3302. SCbots: Stomatocyte-like colloidosomes as versatile microrobots fabricated by one-step self-assembly
3303. Prediction of runoff in ridge-furrow rainwater harvesting system based on SCS-CN model
3304. MiR-301b-3p/3584-5p enhances low-dose mono-n-butyl phthalate (MBP)â€“induced proliferation by targeting Rasd1 in Sertoli cells
3305. Multi-informant prediction of near-term suicidal behavior independent of suicidal ideation
3306. Spontaneous thermal Hall conductance in superconductors with broken time-reversal symmetry
3307. Validade da Escala de EstÃ¡gios de MudanÃ§a = Validity of the Stages of Change Scales
3308. Process and product characteristics for carbonization of liquidâ€“solid mixtures from coal liquefaction
3309. Finite element model for interlayer behavior of double skin steel-concrete-steel sandwich structure with corrugated-strip shear connectors
3310. Prediction of the Punching Load Strength of SCS Slabs with Stud-Bolt Shear Connectors Using Numerical Modeling and GEP Algorithm
3311. Experimental and numerical study of the flexural behavior of steelâ€“concrete-steel sandwich beams with corrugated-strip shear connectors
3312. Optimal Capacitor Allocation in Radial Distribution Networks Using a Combined Optimization Approach
3313. Validation of scs method for runoff estimation
3314. Gravity recovery from SWOT altimetry using geoid height and geoid gradient
3315. A microâ€“macro constitutive model for rock considering breakage effects
3316. Numerical Modelling of the Creep Subsidence of an Ocean Lighthouse Constructed on a Reclaimed Coral Reef Island
3317. Prediction of the long-term settlement of the structures built on a reclaimed coral reef island: an aircraft runway
3318. Depressive symptoms and life satisfaction in Asian American college students: Examining the roles of self-compassion and personal and relational meaning in life
3319. Sound scattering at fluid-fluid rough surface
3320. Development and implementation of a multidimensional narrative support system for emergency nurses: An action research
3321. Optimization of Impervious Surface Space Layout for Prevention of Urban Rainstorm Waterlogging: A Case Study of Guangzhou, China
3322. Advanced Asymmetric Supercapacitor Based on Graphene/Single-Walled Carbon Nanotube and Mesoporous Hollow NiCo2S4 Sub-microsphere Electrodes with High Energy Density
3323. Structural behavior of steel-concrete-steel and steel-ultra-high-performance-concrete-steel composite panels subjected to near-field blast load
3324. Design and optimization of biofuel supply chain network in UK
3325. Late quaternary planktic foraminifer fauna and monsoon upwelling records from the western South China Sea, near the Vietnam margin (IMAGES MD012394)
3326. Is neighbourhood social cohesion associated with subjective well-being for older Chinese people? The neighbourhood social cohesion study
3327. An ensemble learning approach for predicting phenotypes from genotypes
3328. Improvement of snowmelt implementation in the SWAT hydrologic model
3329. A New Method for Analysis of Sliding Cable Structures in Bridge Engineering
3330. Single-cell sequencing and establishment of an 8-gene prognostic model for pancreatic cancer patients
3331. The variability of chlorophyll-a and its relationship with dynamic factors in the basin of the South China Sea
3332. SCSsim: An integrated tool for simulating single-cell genome sequencing data
3333. On the anomalous structure of the Southeast Vietnam Offshore Current during 1994 to 2015
3334. Exactly solvable model of randomly coupled twisted superconducting bilayers
3335. Novel three-dimensional nerve tissue engineering scaffolds and its biocompatibility with Schwann cells
3336. Magnetic stratigraphic dating of marine hydrogenetic ferromanganese crusts
3337. Aggregate packing and interlock evaluation utilizing 2-dimensional synthetic asphalt concrete sections
3338. A framework of dam-break hazard risk mapping for a data-sparse region in Indonesia
3339. Hybrid Internal Combustion Engine based Auxiliary Power Unit
3340. Comparative study concerning the methods of calculation of the critical axial buckling load for stiffened cylindrical shells
3341. An information sharing theory perspective on willingness to share information in supply chains
3342. Estimation of debris flow using Curve Number analysis for The Mount Agung Volcanic event, Bali, Indonesia
3343. Factors associated with cognitive impairment in wellmotivated chronic non-malignant pain patients evaluated for spinal cord stimulation
3344. A lutetium PVC membrane sensor based on (2-oxo-1,2-diphenylethylidene)-N- phenylhydrazinecarbothioamide
3345. Genomic evaluation of Colombian Holstein cattle using imputed genotypes at medium density
3346. Secondary currents and very-large-scale motions in open-channel flow over streamwise ridges
3347. Squamous cell carcinoma antigen-IgM (SCCA-IgM) is associated with interstitial lung disease in systemic sclerosis
3348. Model-Based Optimization of Spinal Cord Stimulation for Inspiratory Muscle Activation
3349. Energy management of a fuel cell/supercapacitor/battery power source for electric vehicular applications
3350. Short form of the Changes in Outlook Questionnaire: translation and validation of the Chinese version
3351. PSSP with dynamic weighted kernel fusion based on SVM-PHGS
3352. Bioheat Model of Spinal Column Heating During High-Density Spinal Cord Stimulation
3353. Tissue Temperature Increases by a 10 kHz Spinal Cord Stimulation System: Phantom and Bioheat Model
3354. Coevolving solutions to the shortest common superstring problem
3355. Intuitive structures: Applications of dynamic simulations in early design stages
3356. Exploring parametric BIM as a conceptual tool for design and building technology teaching
3357. Psychometric testing of the children's resourcefulness scale
3358. Knowledge sharing and protection in data-centric collaborations: An exploratory study
3359. SCSPOD14, a South China Sea physical oceanographic dataset derived from in situ measurements during 1919-2014
3360. Transcriptomics Integrated with Metabolomics: Assessing the Central Metabolism of Different Cells after Cell Differentiation in Aureobasidium pullulans NG
3361. Muscle-derived stem cell exosomes with overexpressed miR-214 promote the regeneration and repair of rat sciatic nerve after crush injury to activate the JAK2/STAT3 pathway by targeting PTEN
3362. Organic molecular markers in PM2.5 on a tropical island in the South China Sea: Temporal variations, sources, and process implications
3363. Three-dimensional imaging of Miocene volcanic effusive and conduit facies: Implications for the magmatism and seafloor spreading of the South China Sea
3364. Moral injury and mental health outcomes among Israeli health and social care workers during the COVID-19 pandemic: A latent class analysis approach
3365. The expression of anger while driving â€“ The role of personality and self-consciousness in a sample of Chinese drivers
3366. Numerical and analytical study of curved steel-concrete-steel sandwich shells under concentrated load
3367. Mode-Matched Disk Resonator Gyroscope in (100) Single Crystal Silicon with Shock Resistance
3368. The analysis of China sea level change
3369. Smart classroom environments affect teacher-student interaction: Evidence from a behavioural sequence analysis
3370. Bridging between SeaWiFS and MODIS for continuity of chlorophyll-a concentration assessments off Southeastern China
3371. Separable compressive imaging method based on singular value decomposition
3372. Analysis of forward scattering properties of rectangular beams in metal space frame radomes
3373. Energy analysis of wind-induced wave and current during typhoon kalmaegi
3374. Fabrication of Multi-Layered Paper-Based Supercapacitor Anode by Growing Cu(OH)2 Nanorods on Oxygen Functional Groups-Rich Sponge-Like Carbon Fibers
3375. Pulsed suction control in a highly loaded compressor cascade with low suction flowrates
3376. Flow separation control using unsteady pulsed suction through endwall bleeding holes in a highly loaded compressor cascade
3377. A flake-like N, O co-doped hierarchical porous carbon derived from chitin with enhanced supercapacitance
3378. Synergistic Cascade Strategy Based on Modifying Tumor Microenvironment for Enhanced Breast Cancer Therapy
3379. 17Î²-estradiol rescues the damage of thiazolidinedione on chicken Sertoli cell proliferation via adiponectin
3380. The sub-fossil diatom distribution in the Beibu Gulf (northwest South China Sea) and related environmental interpretation
3381. A general framework to design secure cloud storage protocol using homomorphic encryption scheme
3382. 'I have high self-compassion': A face-valid single-item self-compassion scale for resource-limited research contexts
3383. Functional tissue-engineered microtissue formed by self-aggregation of cells for peripheral nerve regeneration
3384. Symptom clusters and nutritional status in primary liver cancer patients receiving transcatheter arterial chemoembolization
3385. 17beta-estradiol rescues the damage of thiazolidinedione on chicken Sertoli cell proliferation via adiponectin
3386. Identification of microRNAs for regulating adenosine monophosphate-activated protein kinase expression in immature boar Sertoli cells in vitro
3387. Development of a Modified Bayonet Forceps for Improving Steerability of Paddle Lead Electrodes During Spinal Cord Stimulator Surgery: A Technical Note
3388. A danger model based anomaly detection method for wireless sensor networks
3389. Combination of Schwann cells, small intestinal submucosa and growth factor sustained-release microspheres for repair of peripheral nerve defects
3390. Effect of basic fibroblast growth factor on adhesion and proliferation of Schwann cells on small intestinal submucosa scaffold
3391. Cost-Effective Defense Timing Selection forÂ Moving Target Defense inÂ Satellite Computing Systems
3392. Comparison of SWAT and DLBRM for hydrological modeling of a mountainous watershed in arid northwest China
3393. 16th Asia Simulation Conference on Simulation Multi-Conference, AsiaSim/SCS AutumnSim 2016
3394. Comparing paper level classifications across different methods and systems: an investigation of Nature publications
3395. SST Forecast Skills Based on Hybrid Deep Learning Models: With Applications to the South China Sea
3396. Controlling factor analysis of oceanic surface pCO2 in the South China Sea using a three-dimensional high-resolution biogeochemical model
3397. Using softened contact relationship describing compressible membrane in FEA of spiral case structure
3398. Sliding behaviour of steel liners on surrounding concrete in c-cross-sections of spiral case structures
3399. Ultrasonic Evaluation of Liver Fibrosis Coexisting with Hepatic Steatosis Using the Homodyned K Distribution Combined with Noise-modulated Empirical Mode Decomposition
3400. The Wind Effect on Interferometric Altimeter Validation Using Steric Method in South China Sea
3401. Identification of hub biomarkers of myocardial infarction by single-cell sequencing, bioinformatics, and machine learning
3402. CSCdroid: Accurately Detect Android Malware via Contribution-Level-Based System Call Categorization
3403. The Fears of Compassion in Sport Scale: A short, context-specific measure of fear of self-compassion and receiving compassion from others validated in UK athletes
3404. Statistical Analysis of Mesoscale Eddies Entering the Continental Shelf of the Northern South China Sea
3405. In situ ice template approach to fabricate Ag modified 3D Ti3C2Tx film electrode for supercapacitors
3406. Paper with Power: Engraving 2D Materials on 3D Structures for Printed, High-Performance, Binder-Free, and All-Solid-State Supercapacitors
3407. Estimation of heavy metal pollution loads from non-point sources based on GIS/RS
3408. Model-based optimization of spinal cord stimulation
3409. Modeling effects of spinal cord stimulation on wide-dynamic range dorsal horn neurons: influence of stimulation frequency and GABAergic inhibition
3410. Shear resistance behavior of partially composite Steel-Concrete-Steel sandwich beams considering bond-slip effect
3411. Numerical-based analytical model of double-layer steel-LHDCC sandwich composites under punching loads
3412. AMPK regulates immature boar Sertoli cell proliferation through affecting CDK4/Cyclin D3 pathway and mitochondrial function
3413. Model Predictive Control Based Dynamic Power Loss Prediction for Hybrid Energy Storage System in DC Microgrids
3414. ZnSO4 rescued vimentin from collapse in DBP-exposed Sertoli cells by attenuating ER stress and apoptosis
3415. Sensitivity analysis of potential evapotranspiration to key climatic factors in the shiyang river basin
3416. Lighting environmental assessment in enclosed spaces based on emotional model
3417. Approach for estimating the vertical distribution of the diffuse attenuation coefficient in the South China Sea
3418. Reliability and validity of the parent form of the social competence scale in Chinese preschoolers
3419. Synergistic effect of schwann cells and retinoic acid on differentiation and synaptogenesis of hippocampal neural stem cells in vitro
3420. Robust electrodes based on coaxial TiC/C-MnO2 core/shell nanofiber arrays with excellent cycling stability for high-performance supercapacitors
3421. Reliability and validity of the Social Curiosity Scale among Chinese university students
3422. Development of a hydrological response index to represent TOPMODEL parameters
3423. A power-speed hierarchical optimization framework of diesel/battery/supercapacitor vehicular hybrid propulsion systems
3424. Steering measurement decomposition for vehicle lane keeping â€“ A study of driver behaviour
3425. The causal role of South China Sea on the Pacificâ€“North American teleconnection pattern
3426. RetCom: Information Retrieval-Enhanced Automatic Source-Code Summarization
3427. The Illness Perceptions and Coping Experiences of Patients with Colorectal Cancer and Their Spousal Caregivers: A Qualitative Study
3428. Mechanisms of change underlying mindfulness-based practice among adolescents
3429. Reliability and validity of the Social Curiosity Scale among Chinese university students
3430. Platelet-rich plasma-derived exosomes enhance mesenchymal stem cell paracrine function and nerve regeneration potential
3431. Embedding cryptographic features in compressive sensing
3432. Microbial metabolic characteristics of carbon in the black soil parent material maturation process
3433. A generative method for steganography by cover synthesis with auxiliary semantics
3434. Using 1/2 Descending Time in CEUS to Identify Renal Allograft Rejection
3435. Path analysis of the association between self-compassion and depressive symptoms among nursing and medical students: a cross-sectional survey
3436. Estimation of primary production from the light absorption of phytoplankton and photosynthetically active radiation in the South China Sea
3437. Phytoplankton Increases Induced by Tropical Cyclones in the South China Sea During 1998â€“2015
3438. Using kin discrimination to construct synthetic microbial communities of Bacillus subtilis strains impacts the growth of black soldier fly larvae
3439. Exploratory factor analysis of the Cancer Problems in Living Scale: a report from the American Cancer Society's Studies of Cancer Survivors
3440. All-fiber structure covered with two-dimensional conductive MOF materials to construct a comfortable, breathable and high-quality self-powered wearable sensor system
3441. Internal wave refraction observed from sequential satellite images
3442. Fabrication of CoMo-layered double hydroxide@Co2MoO4 nanosheets for enhancing the performance of asymmetric capacitors
3443. Chitosan degradation products facilitate peripheral nerve regeneration by improving macrophage-constructed microenvironments
[truncated: 16,461 more chars]
